# Supplementary material for: Diaryl-Pyrano-Chromenes Atropisomers: Stereodynamics and Conformational Studies
Source: Molecules. 2023 Jun 22;28(13):4915. doi: 10.3390/molecules28134915 (PMC10343549; doi:10.3390/molecules28134915)
Supplement: Supplementary file 1 [file molecules-28-04915-s001.zip › molecules-2445498-supplementary.pdf]

Supporting Information

Diaryl-pyrano-chromenes atropisomers: stereodynamics and conformational studies

Alessia Ciogli [1,*], Andrea Fochetti [1], Andrea Sorato [1], Giancarlo Fabrizi [1], Nunzio Matera [2], Andrea Mazzanti [2] and Michele Mancinelli [2,*]

[1] Department of Chemistry and Drug Technologies, Sapienza University of Rome, Piazzale Aldo Moro 5, 00185 Roma, Italy; andrea.fochetti@uniroma1.it (A.F.); andrea.sorato@uniroma1.it (A.S.); giancarlo.fabrizi@uniroma1.it (G.F.)

[2] Department of Industrial Chemistry "Toso Montanari", University of Bologna, Viale del Risorgimento 4, 40136 Bologna, Italy; nunzio.matera2@unibo.it (N.M.); andrea.mazzanti@unibo.it (A.M.)

* Correspondence: alessia.ciogli@uniroma1.it (A.C.); michele.mancinelli@unibo.it (M.M.)

Table of content

Synthesis of compound 5 and its NMR Spectra	S2-S3
Separation of compounds 1a and 2a	S4
Full characterization compound 1a	S5-S7
Full characterization compound 2a	S8-S13
Synthesis of compound 1b and 2b	S14-S15
Full characterization compound 1b	S16-S26
Full characterization compound 2b	S27-S33
MOs for compounds 1b and 2b	S34-S36
DFT calculations	S37-S89

1. Synthesis of 1,3-bis((3-(2-methylnaphthalen-1-yl)prop-2-yn-1-yl)oxy)benzene (5)

In a 50 ml round botom flask equipped with a magnetic stirring bar, resorcinol (144.8 mg, 1.3 mmol., 1 equiv.) was dissolved in DMF (6 mL) at room temperature. Then K_2CO_3 (545 mg, 3.95 mmol., 3 equiv.) was added and after 15 minutes 1-(3-bromoprop-1-yn-1-yl)-2-methylnaphthalene (750 mg, 2.89 mmol., 2.2 equiv.) was added to the mixture. Reaction was monitored by TLC until disappearance of the starting material, then diluted with Et_2O and washed with $NaHSO_4$ (x2) and brine (x2). The organic extract was dried over Na_2SO_4 and concentrated under reduced pressure. The residue was purified by chromatography on SiO_2 (25-40 µm), eluting with a 98/2 (v/v) *n*-hexane/AcOEt mixture to obtain 589.0 mg (97% yield) of 1,3-bis((3-(2-methylnaphthalen-1-yl)prop-2-yn-1-yl)oxy)benzene **5**.

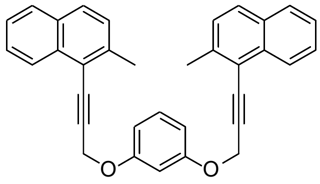

5

5: pale yellow solid; 97% yield; ^{1}H NMR (400 MHz) ($CDCl_3$) δ (ppm) 8.25 (d, *J* = 8.4 Hz, 2H), 7.79 (d, *J* = 8.1 Hz, 2H), 7.52 (t, *J* = 7.3 Hz, 2H), 7.42 (t, *J* = 7.5 Hz, 2H), 7.36 – 7.28 (m, 3H), 6.97 (m, 1H), 6.84 (dd, J_1 = 8.2 Hz, J_2 = 2.2 Hz, 2H), 5.17 (s, 4H), 2.60 8s, 6H); ^{13}C{^{1}H} NMR (100.6 MHz) ($CDCl_3$) δ (ppm) 159.0, 139.8, 133.7, 131.4, 130.0 , 128.5, 128.0, 127.9, 126.9, 125.8, 125.5, 118.3, 108.3, 103.2, 57.0, 21.3.

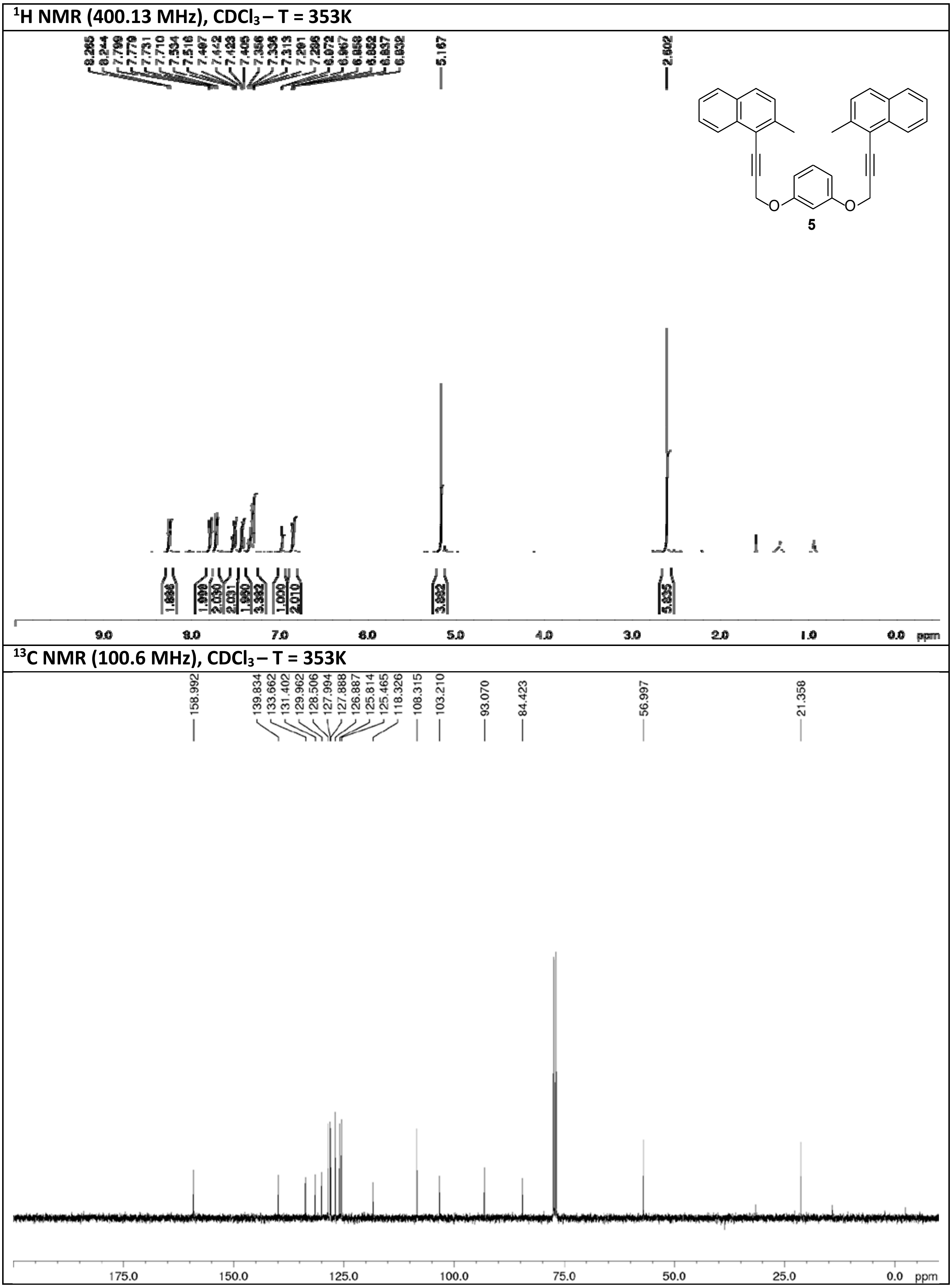

Figure S1. ^{1}H NMR and ^{13}C NMR spectra of compound **5** at +25 °C.

2. Separation of compounds 1a/2a

Linear and angulated **1a**/**2a** were separated by semipreparative HPLC on silica (a). Column: Silica Adamas (250*10 mm ID), eluent *n*-Hex/DCM 50/50, flow: 4.0 ml/min, Detector: UV 254 nm. Analytical controls of purified fraction are in Figure S2.

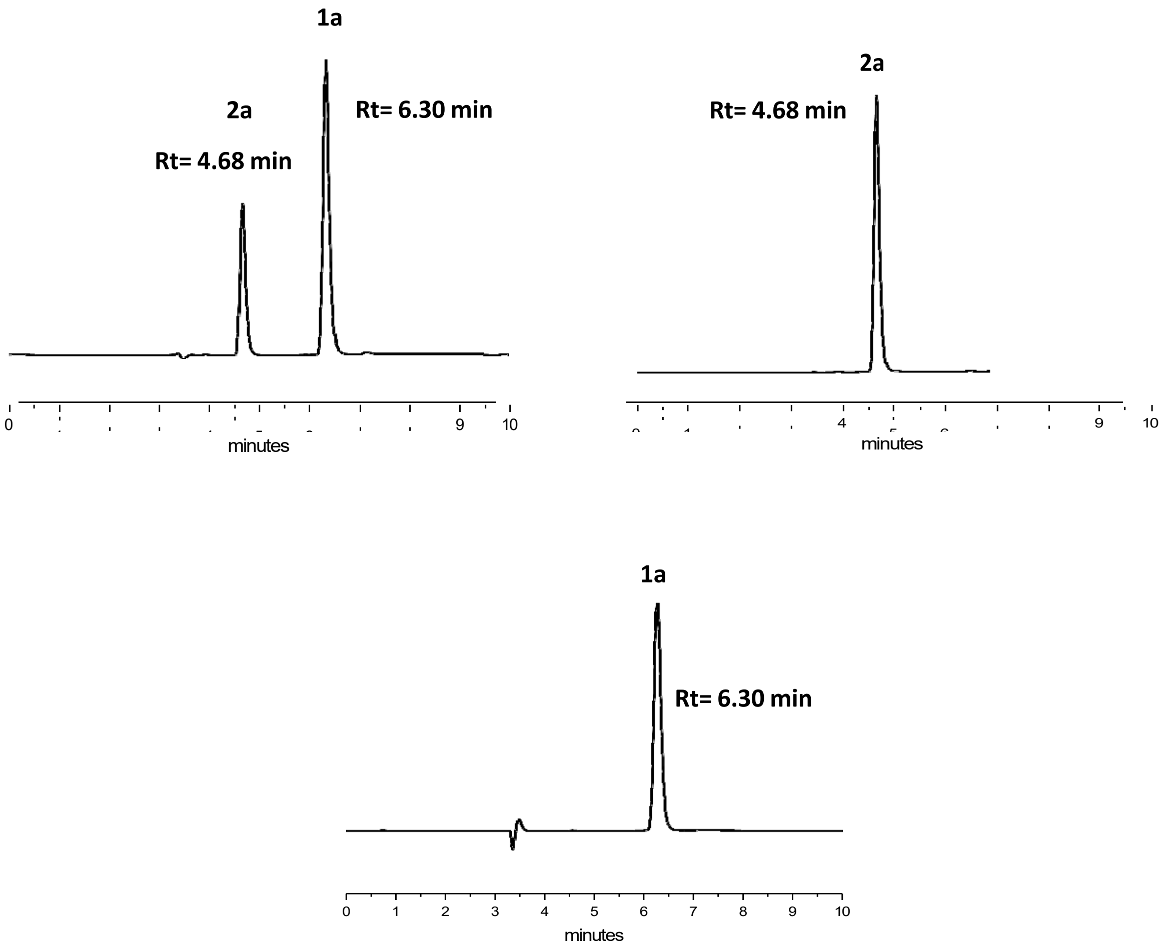

Figure S2. Column: Silica Adamas (250*4.6 mm ID), eluent *n*-Hex/DCM 50/50, flow: 1 ml/min, Detector: UV 254 nm. Isolated product purity: **1a** 99.9 % (c) and **2a** 99.9% (b).

3. Characterization of compound 1a

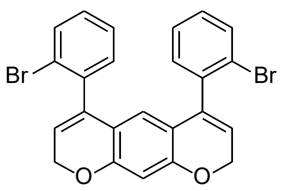

1a

1a (mixture of stereoisomers): white solid; mp = 159 - 161 °C; IR (neat): 2925, 2837, 1676, 1576, 1488, 1427 cm^{-1}; ^{1}H-NMR (400.13 MHz, $CDCl_3$, +25 °C): δ 7.45 (m, 2 H), 7.19 - 7.16 (m, 3H), 7.06 (dt, J_1 = 7.6 Hz, J_2 = 1.8 Hz, 3H), 6.42 (s, 1H), 5.80 - 5.74 (m, 1H), 5.55 (s, 2H), 4.95 - 4.88 (m, 4H); ^{13}C{^{1}H} NMR (100.6 MHz, DMSO-d6, +80°C): δ 155.4, 138.7, 136.0, 132.6, 131.2, 129.1, 127.2, 123.7, 123.2, 118.5, 116.4, 104.0, 65.7; HRMS: *m/z* [M + H]$^+$ calcd for $C_{24}H_{17}Br_2O_2$: 496.9569; found: 496.9565.

Additional ^{1}H-NMR e ^{13}C-NMR at 80 °C in DMSO-d6 were recorded to obtain mediated signals for a good quality of spectra considering the faster interconversion of *syn*/*anti* stereoisomers.

^{1}H NMR (400.13 MHz), $CDCl_3$ – T = 298K

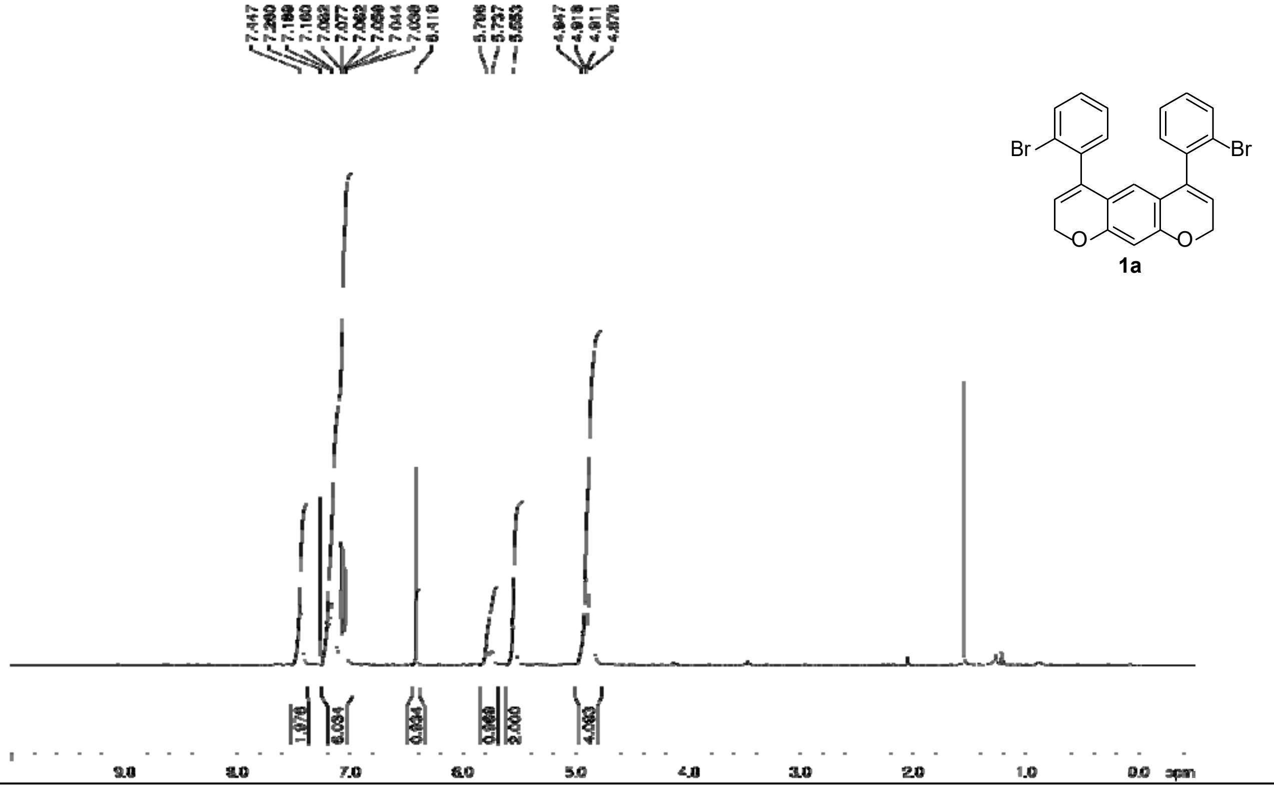

^{13}C NMR (100.6 MHz), $CDCl_3$ – T = 298K

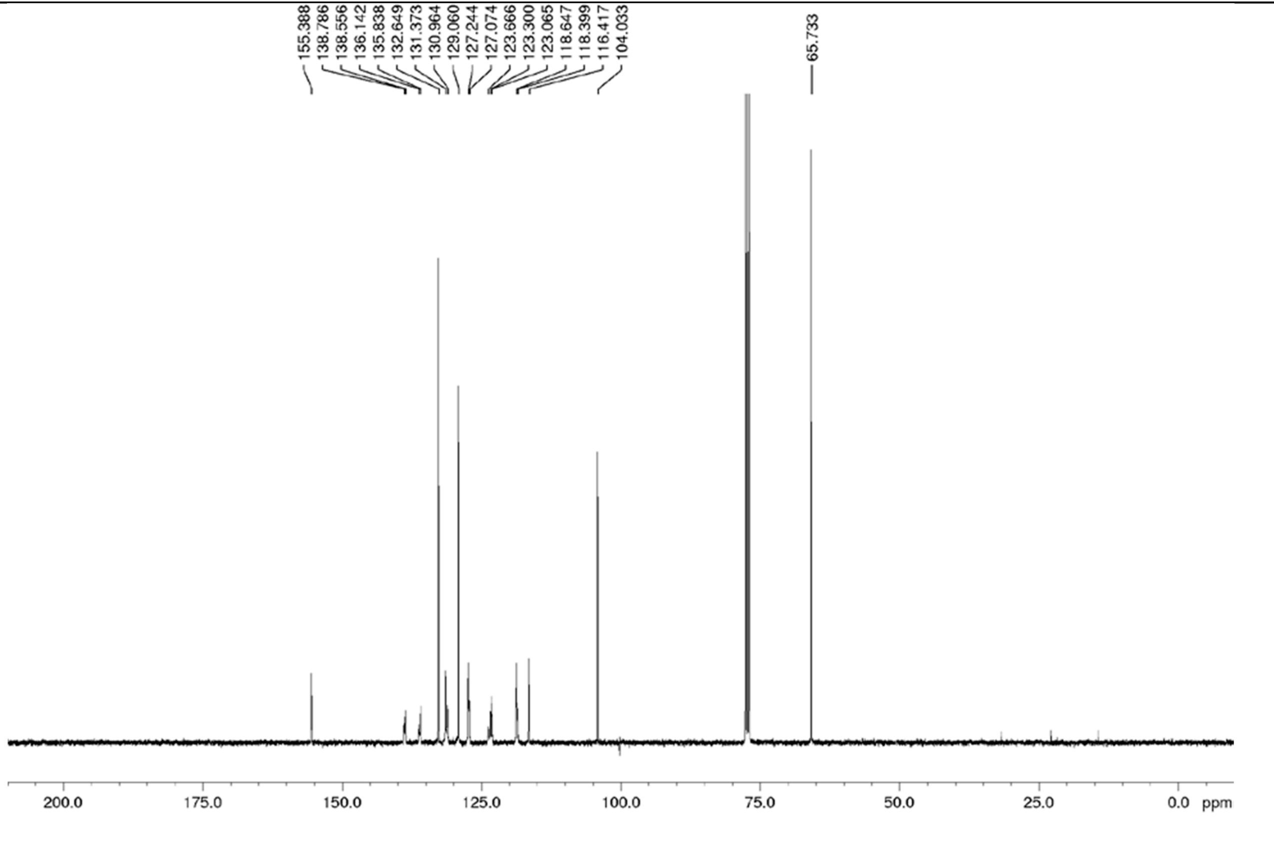

Figure S3. ^{1}H NMR and ^{13}C NMR spectra of compound **1a** at +25 °C.

^{1}H NMR (400.13 MHz), DMSO – T = 353K

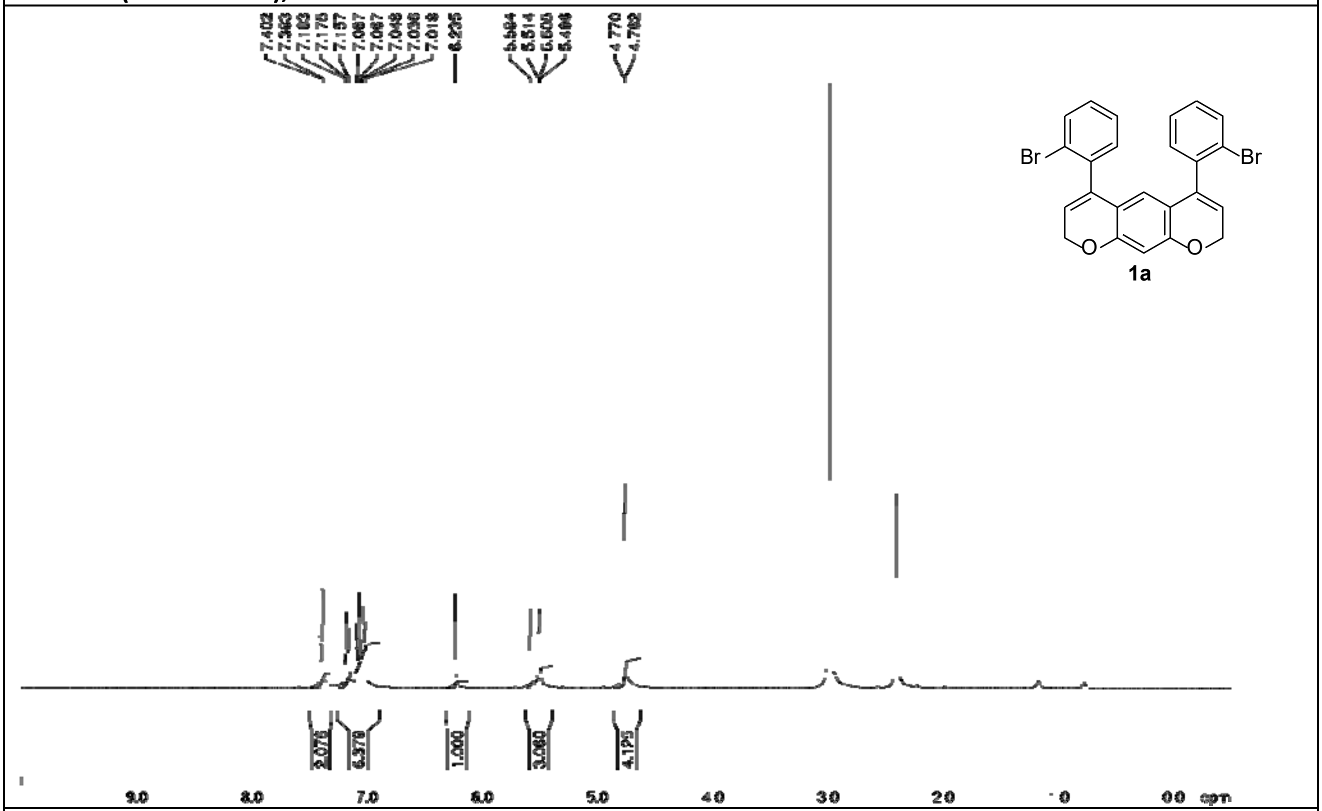

^{13}C NMR (100.6 MHz), DMSO – T = 353K

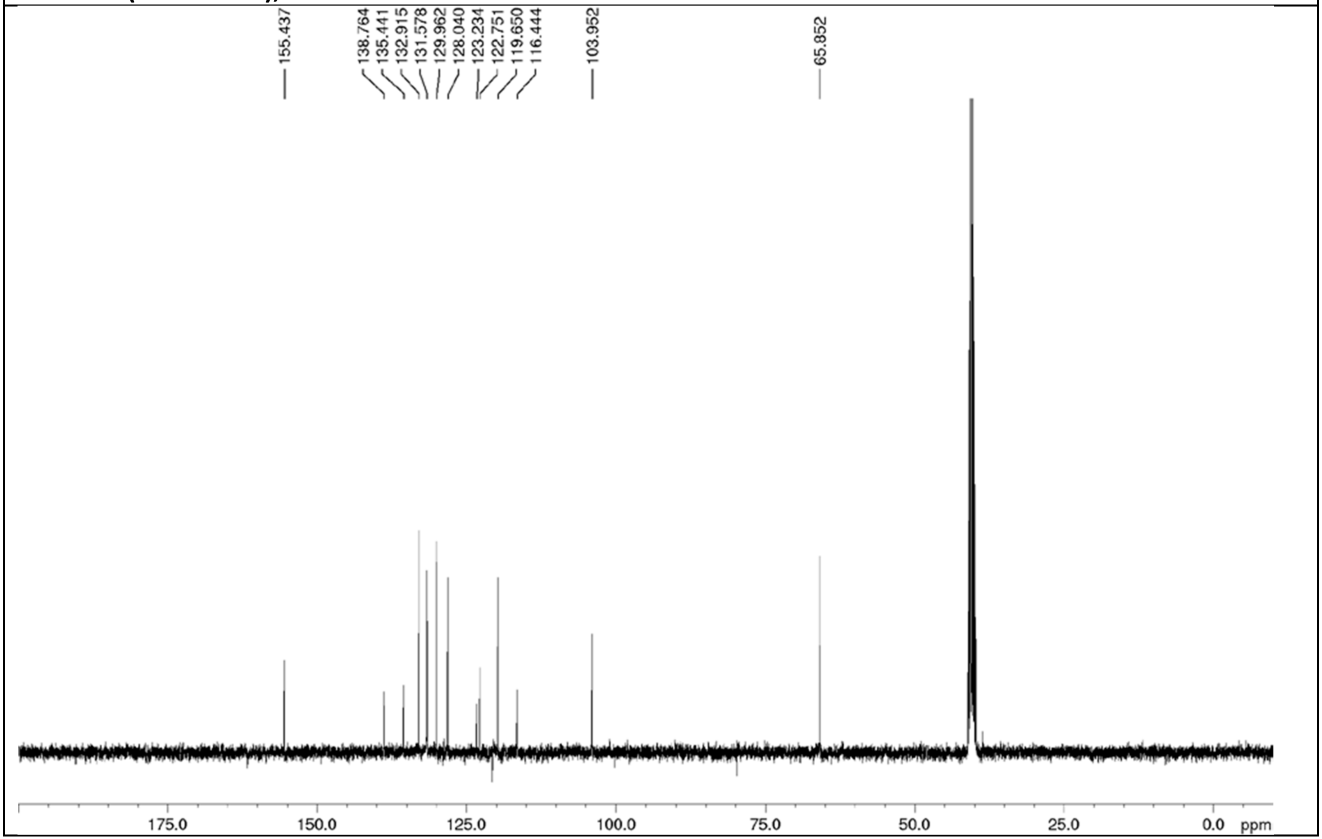

Figure S4. ^{1}H NMR and ^{13}C NMR spectra of compound **1a** at +80 °C.

4. Full characterization of compound 2a

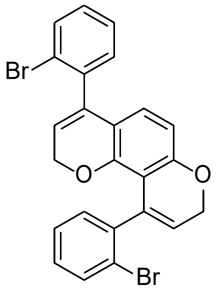

2a

2a (mixture of stereoisomers): yellow oil; IR (neat): 2930, 2835, 1676, 1575, 1490, 1427 cm^{-1}; ^{1}H NMR (400.13 MHz) ($CDCl_3$): 7.55-7.47 (m, 2H), 7.27-6.98 (m, 6H), 6.36 (s, 2H), 5.65 (bs, 1H), 5.39 (bs, 1H), 4.76 - 4.61 (m, 2H), 4.42 - 4.33 (m, 1H), 4.22 - 4.14 (m, 1H); $^{13}C\{^{1}H\}$ NMR (100.6 MHz) ($CDCl_3$): δ 155.7, 150.6, 142.6, 139.4, 136.5, 135.3, 133.0, 132.0, 131.4, 130.0, 129.3, 128.3, 127.5, 127.0, 126.3, 123.8, 122.6, 122.2, 118.9, 117.8, 112.4, 109.3, 65.0, 64.9; HRMS: *m/z* $[M + H]^+$ calcd for $C_{24}H_{17}Br_2O_2$: 496.9569; found: 496.9565.

^1H NMR (400.13 MHz), $CDCl_3$ – T = 298 K

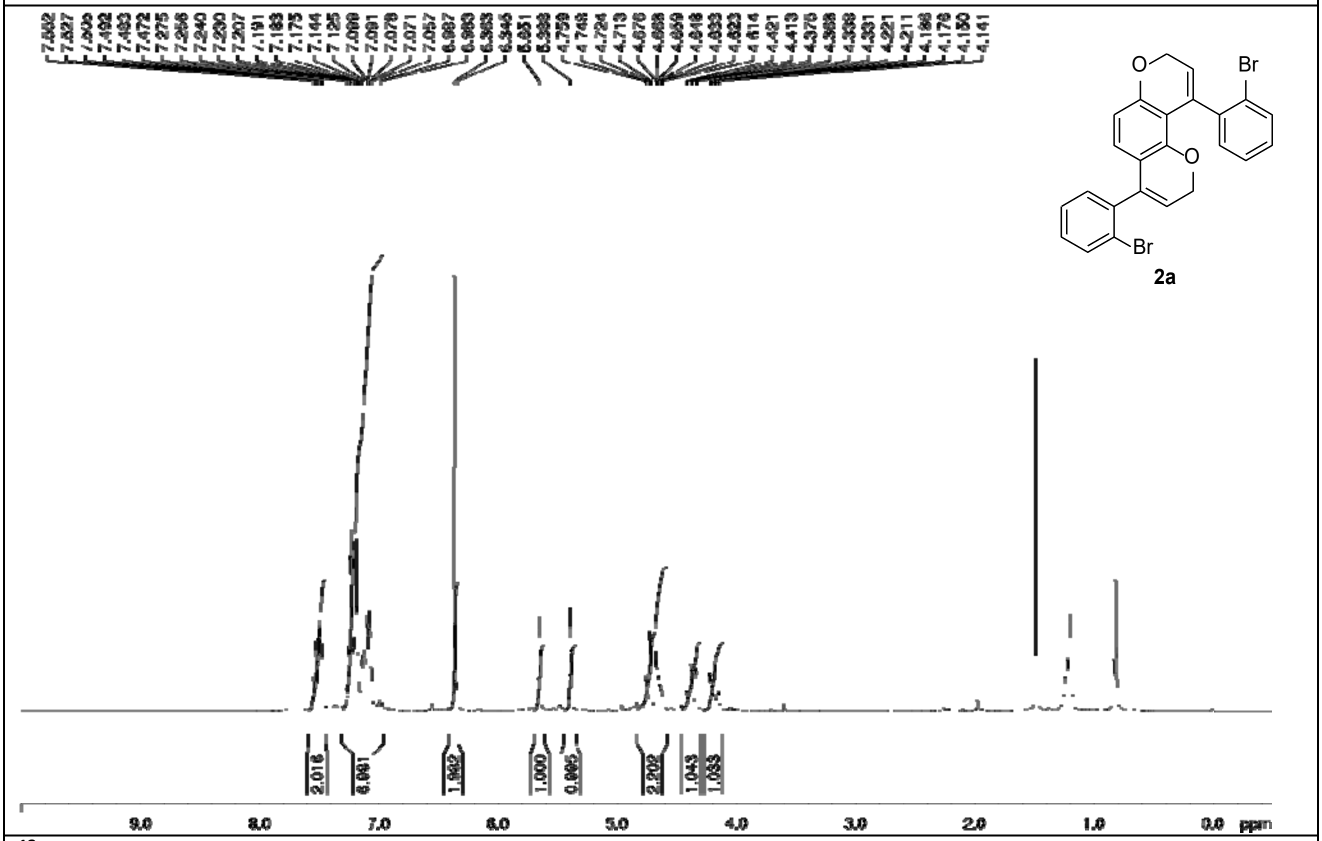

^{13}C NMR (100.6 MHz), $CDCl_3$ - T = 298K

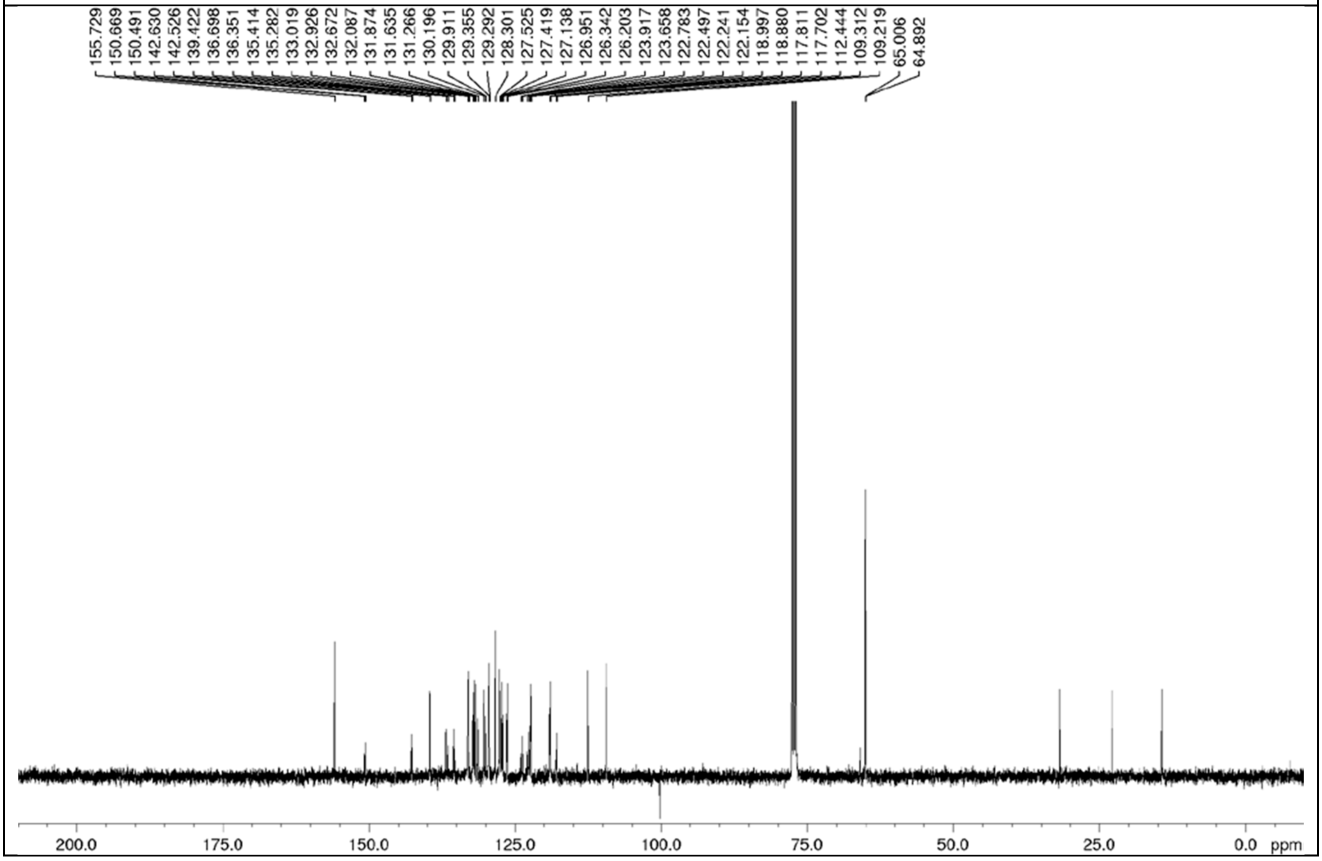

Figure S5. ^1H NMR and ^{13}C NMR spectra of compound **2a** at +25 °C.

4.1 DFT Calculations of *syn*/*an'ti* for compounds 2a

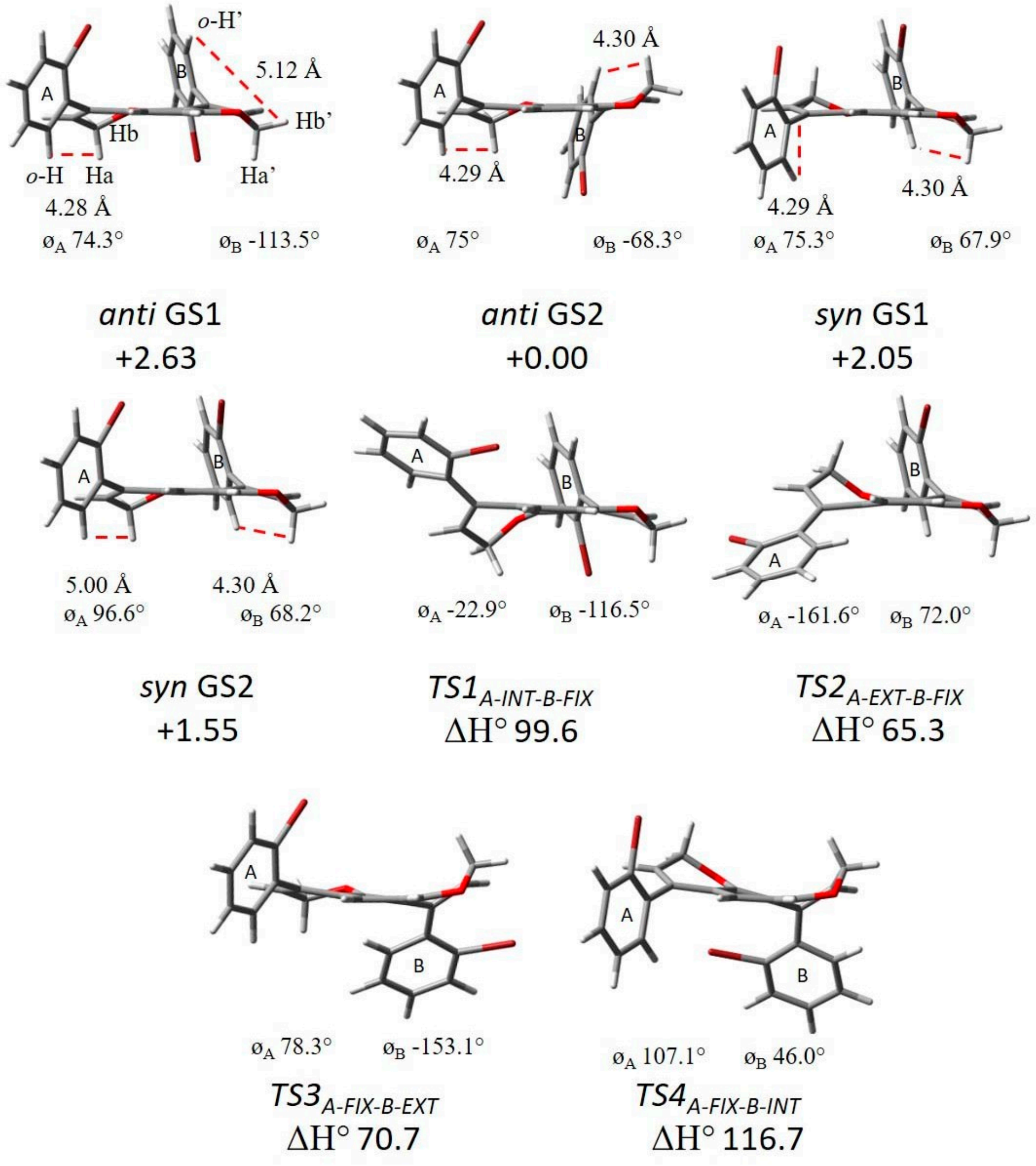

Figure S6. Predicted DFT (6-311++G(d,p), PCM=chloroform) conformations of *syn*/*anti* for compounds **2a** are shown. The relative Enthalpy energies are reported in kJ/mol.

Table S1. Descriptors are shown: r = atom distance; ø = dihedral angle

2a	r (H_a - *o*H)	r (H_b - *o*H)	r ($H_{a'}$ - *o*H')	r ($H_{b'}$ - *o*H')	$ø_A$	$ø_B$	% *pop*
GS1 *anti*	4.28	5.09	5.40	5.12	74.30	-113.50	15.74
GS2 *anti*	4.29	5.11	4.30	4.99	75.00	-68.30	43.71
GS1 *syn*	4.29	5.11	4.30	4.99	75.30	67.90	16.16
GS2 *syn*	5.41	5.00	4.30	4.99	96.60	68.20	24.37
avg. *anti vs syn*	4.29 vs 4.96	5.10 vs 5.04	4.59 vs 4.29	5.02 vs 4.99	74.8 vs 88.1	-80.3 vs 68.1	59.5/40.5

4.2 DNMR experiment of compound 2a.

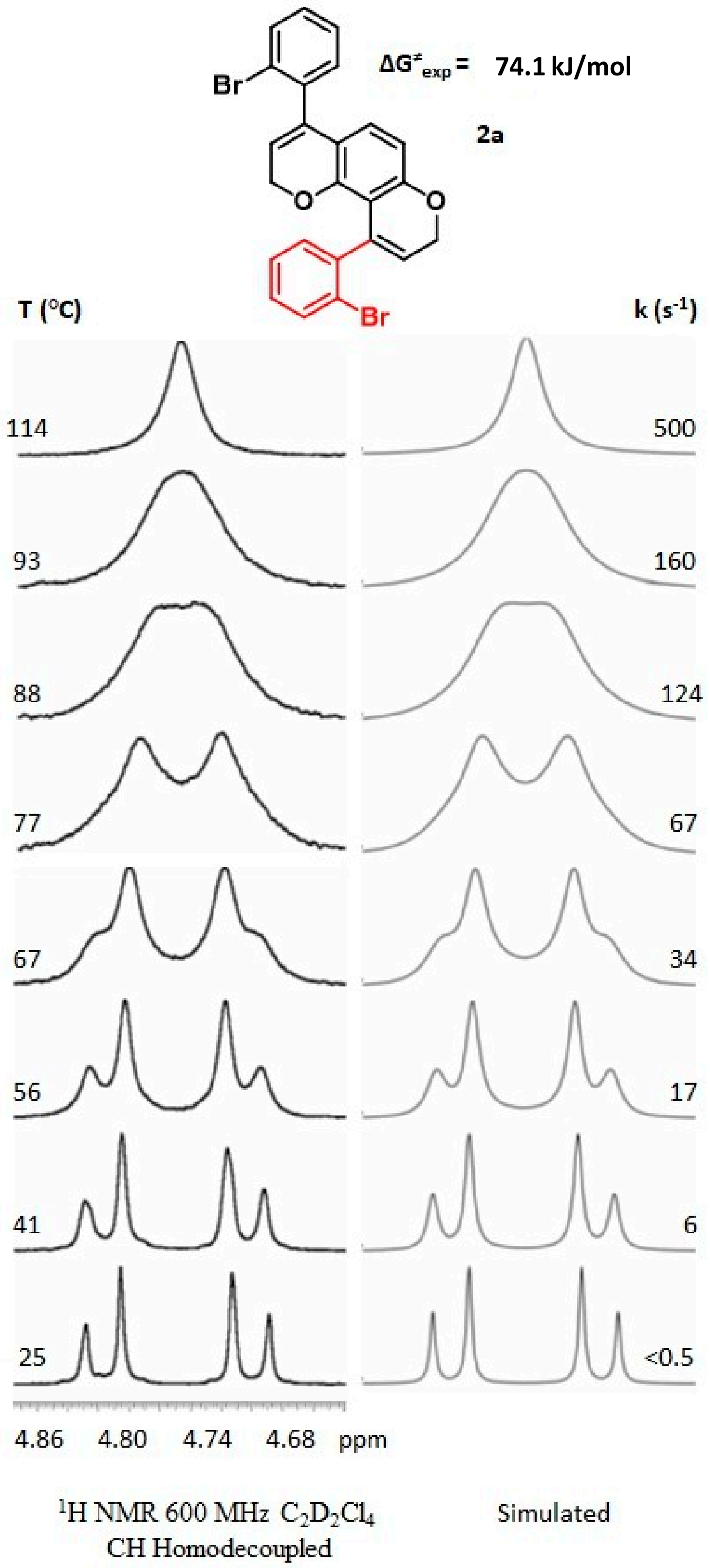

Figure S7. Simulated and experimental dynamic ^{1}H NMR (600 MHz) spectra in $C_2D_2Cl_4$ of compound **2a** for the enantiomerization process. For each temperature (left column) correspond a kinetic constant (right column). The vinylic ^{1}H NMR signal at 5.77 ppm was homo-decoupled to simplify the spin system of adjacent CH_2 from ABX to AB system.

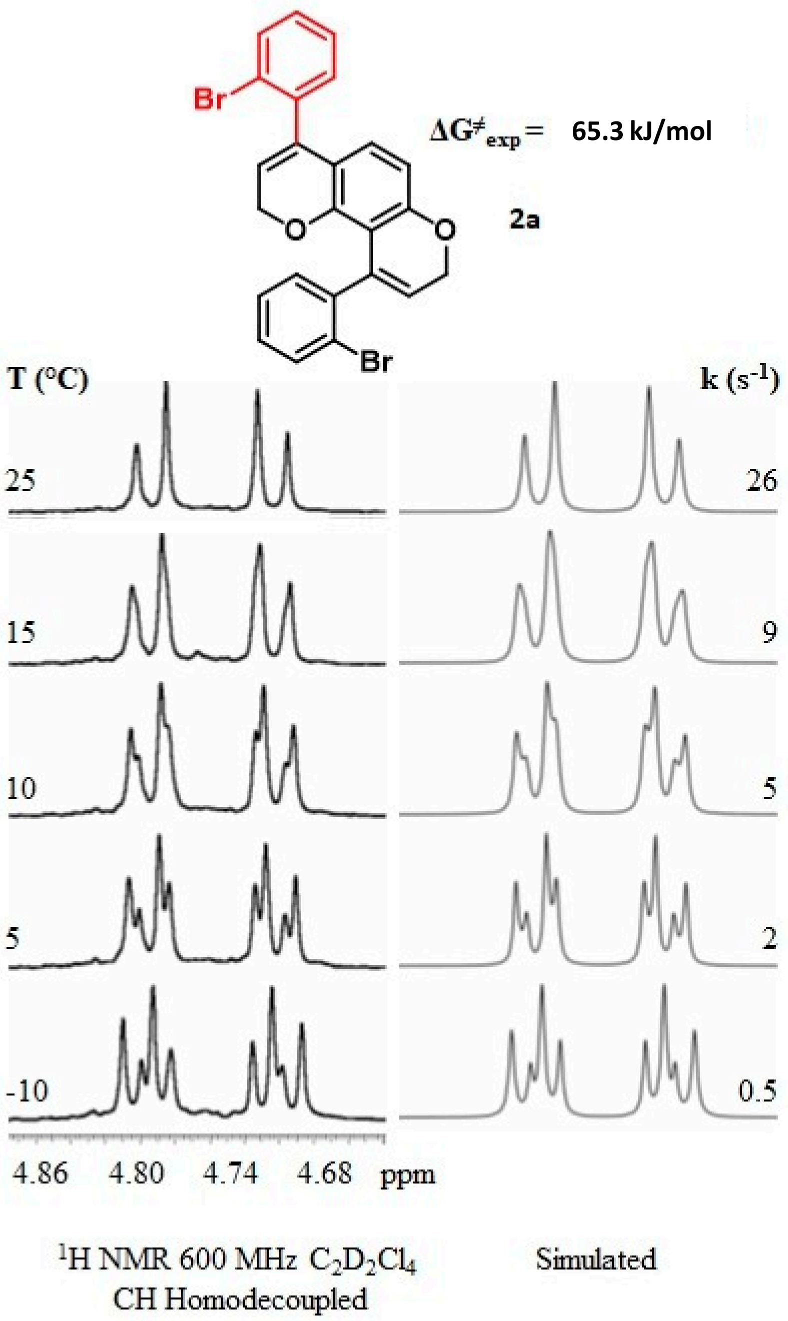

Figure S8. Simulated and experimental dynamic ^{1}H NMR (600 MHz) spectra in $C_2D_2Cl_4$ of compound **2a** for the diastereomerization process. For each temperature (left column) correspond a kinetic constant (right column). The vinylic ^{1}H NMR signal at 5.77 ppm was homo-decoupled to simplify the spin system of adjacent CH_2 from ABX to AB system.

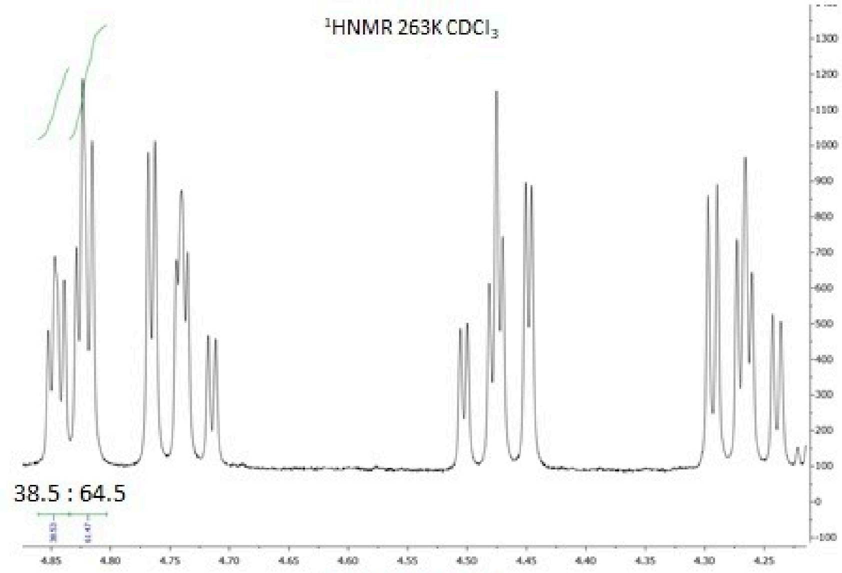

2a	anti/syn ratio
Computed chloroform 263K	61:39
¹HNMR $CDCl_3$ 263K	61.5:38.5*
Computed dipole moment $CHCl_3$	2.45:4.60 (Debye)

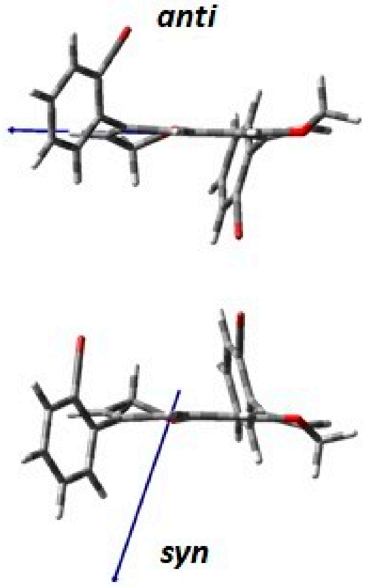

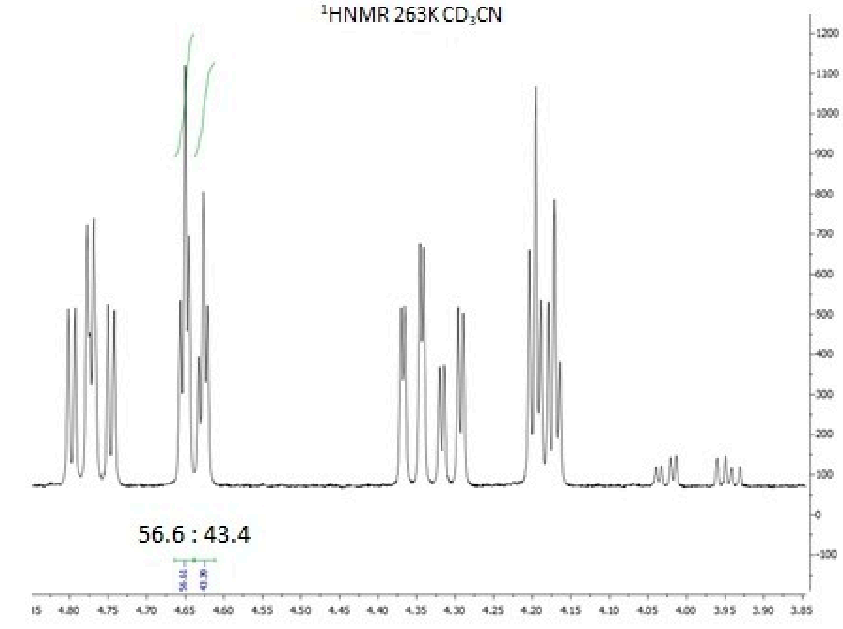

2a	anti/syn ratio
Computed acetonitrile 263K	56:44
¹HNMR CD_3CN 263K	56.6:43.4*
Computed dipole moment ACN	2.78:5.35 (Debye)

Figure S9. top: recorded ^{1}H NMR spectra of **2a** at -10 °C (263 K) in $CDCl_3$, CH_2 signals (ABX system region). bottom: recorded ^{1}H NMR spectra of **2a** at -10 °C (263 K) in CD_3CN, CH_2 signals (ABX system region).

* Computed dipole moments were averaged using the Boltzmann population of the single GSs.

5. Synthesis of 4,6-bis(2-methylnaphthalen-1-yl)-2*H*,8*H*-pyrano[3,2-g]chromene (1b) and 4,10-bis(2-methylnaphthalen-1-yl)-2*H*,8*H*-pyrano[2,3-f]chromene (2b):

In a 50 mL Carousel Tube Reactor (Radely Discovery Technology) containing a magnetic stirring bar 1,3-bis((3-(2-methylnaphthalen-1-yl)prop-2-yn-1-yl)oxy)benzene (121 mg, 0.26 mmol, 1 equiv.) was dissolved in CH_2Cl_2 (2 mL) at room temperature. Then [tris(2,4-di-*tert*-butylphenyl)phosphite]gold(I) chloride (9.1 mg, 0.01 mmol., 0.04 equiv.) was added followed by $AgSbF_6$ (3.6 mg, 0.01 mmol., 0.04 equiv.). The mixture was allowed to stir for an hour and then CH_2Cl_2 was evaporated under reduced pressure. The residue was purified by chromatography on SiO_2 (25-40 µm), eluting with a 97/3 (v/v) *n*-hexane/AcOEt mixture to obtain a mixture of 4,6-bis(2-methylnaphthalen-1-yl)-2*H*,8*H*-pyrano[3,2-g]chromene **1b** and 4,10-bis(2-methylnaphthalen-1-yl)-2*H*,8*H*-pyrano[2,3-f]chromene **2b** in a ratio of 87/13. For product characterization see following sections.

^{1}H NMR (400.13 MHz), $CDCl_3$ – T = 298K

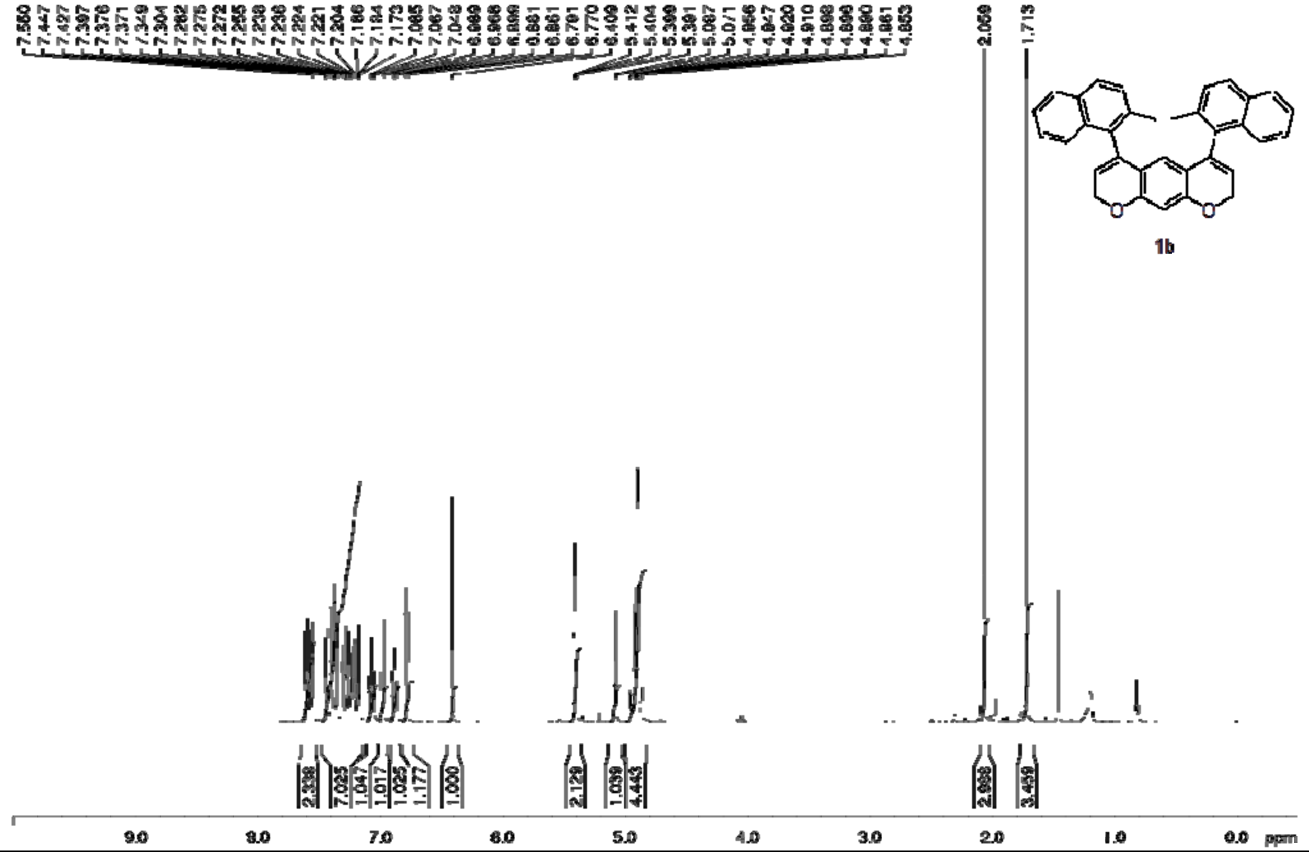

^{13}C NMR (100.6 MHz), $CDCl_3$ – T = 298K

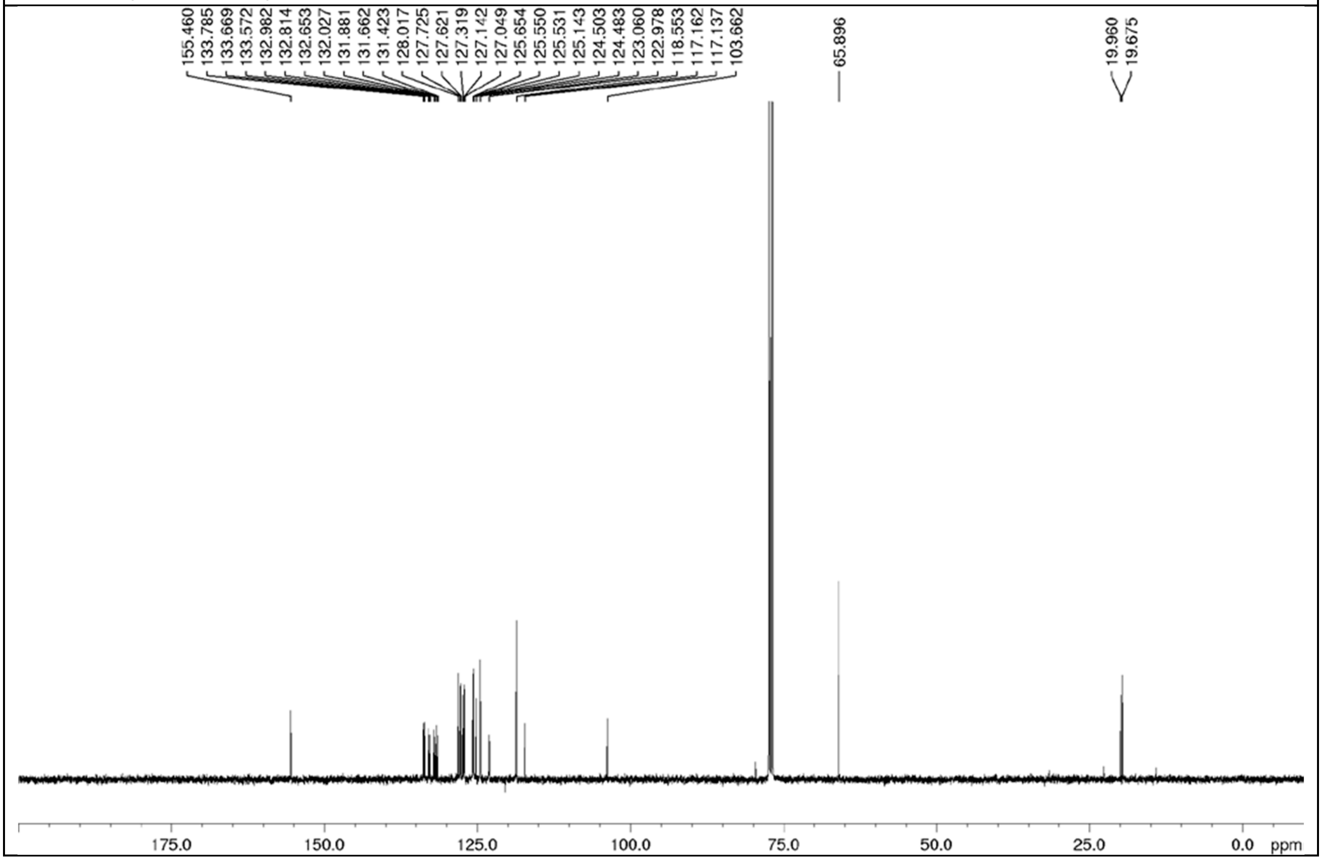

Figure S10. ^{1}H NMR and ^{13}C NMR spectra of compound **1b mixture of isomers** at +25 °C.

6. Full characterization of *syn/anti* 1b

Syn/Anti stereoisomers were separated by semipreparative HPLC on (*S,S*)-Whelk-O2 10 micron (250*10 mm L*ID) by using hexane/dichloromethane 95/5 + 0.1% ethanol at flow rate of 4.0 ml/min. Detector UV 254 nm. In the analytical version, geometry of column was 150*4.6 mm L*ID (chromatographic trace in **figure S11** (black trace). *Anti* and *syn* stereoisomers were assigned based on CD signal (see red trace on the same figure). After separation, ^{1}H-NMR spectra of two stereoisomer were acquired.

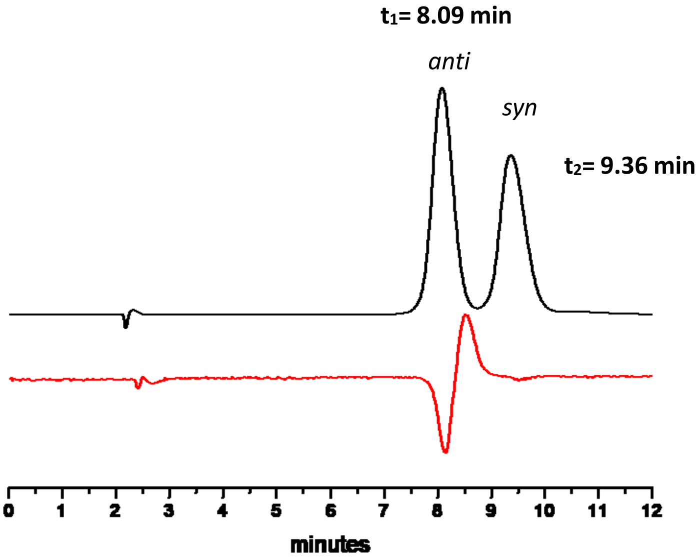

Figure S11. HPLC separation on (*S,S*)-Whelk-O2 10 micron (250*10 mm L*ID) by using *n*-hexane/dichloromethane 95/5 + 0.1% ethanol at flow rate of 4.0 ml/min. Detector UV 254 nm. In the analytical version, geometry of column was 150*4.6 mm L*ID (chromatographic black trace). *Anti* and *syn* stereoisomers were assigned based on CD signal (red trace).

1b (*syn*): 1H NMR (600 MHz) (CDCl3): d (ppm) 7.51 (d, J = 5.4 Hz, 2H), 7.44 (d, J = 5.5 Hz, 2H), 7.37 (d, J = 5.6 Hz, 2H), 7.16 – 7.13 (m, 2H), 7.06 (d, J = 5.6 Hz, 2H), 6.97 – 6.94 (m, 2H), 6.48 (s, 1H), 5.48 (t, J = 2.4 Hz, 2H), 5.16 (s, 1H), 5.00 – 4.94 (m, 4H), 2.13 (s, 6H); 13C{1H} NMR (150 MHz) (CDCl3): d (ppm) 155.4, 133.8, 133.0, 132.8, 131.9, 131.4, 127.7, 127.3, 127.1, 125.5, 125.1, 124.5, 123.0, 118.5, 117.1, 103.6, 65.9, 19.9.

1b (*anti*): 1H NMR (600 MHz) (CDCl3): d (ppm) 7.68 (d, J = 5.3 z, 2H), 7.63 (d, J = 5.5 Hz, 2H), 7.46 (d, J = 5.6 Hz, 2H), 7.35 – 7.27 (m, 4H), 6.86 (d, J = 5.6 Hz, 2H), 6.48 (s, 1H), 5.48 (t, J = 2.4 Hz, 2H), 5.14 (s, 1H), 5.03 – 4.94 (m, 4H), 1.79 (s, 6H); 13C{1H} NMR (150 MHz) (CDCl3): d (ppm) 155.5, 133.7, 133.6, 132.6, 132.0, 131.7, 128.0, 127.6, 127.0, 125.6, 125.5, 124.5, 123.0, 118.5, 117.1, 103.6, 65.9, 19.6.

^{1}H NMR (600 MHz), $CDCl_3$ – T = 298K

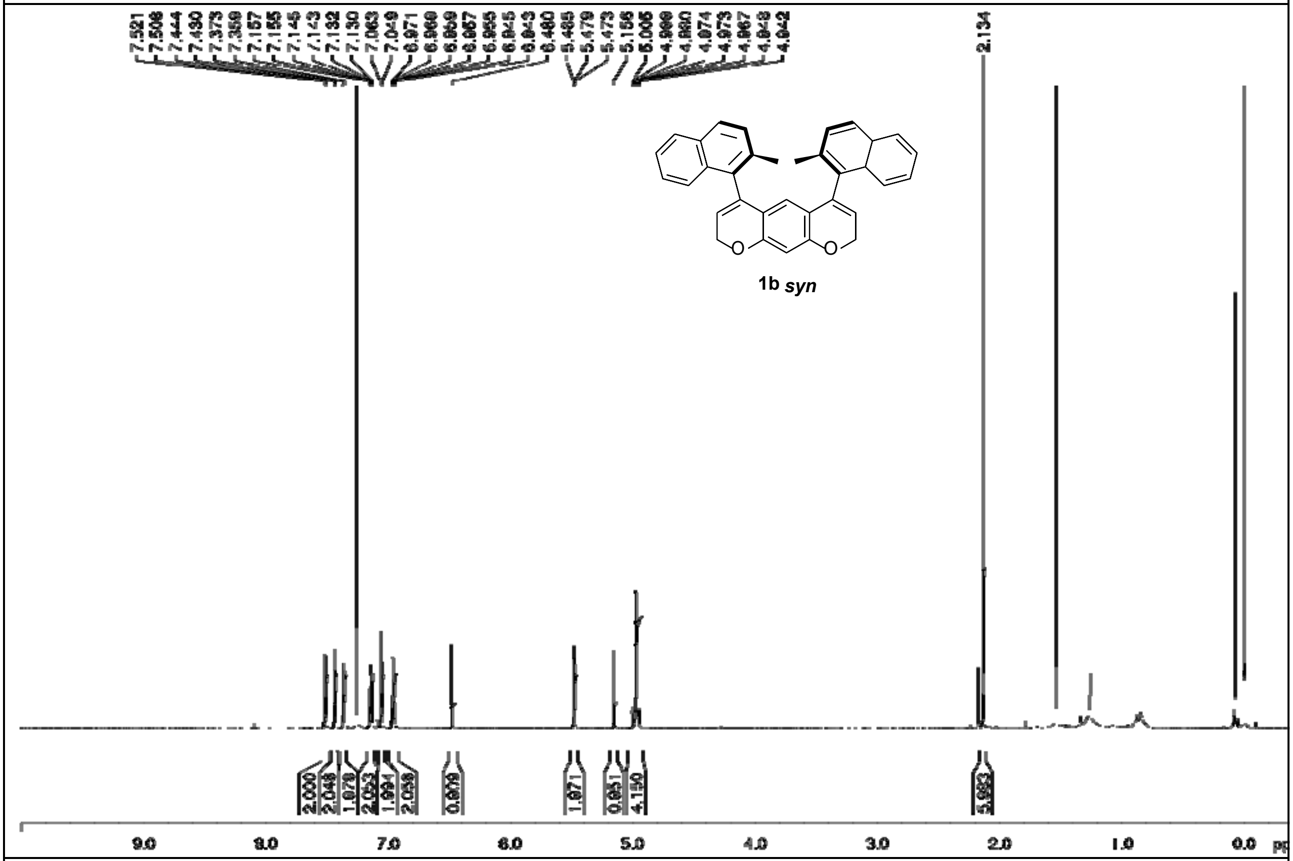

^{13}C NMR (150 MHz), $CDCl_3$ – T = 298K

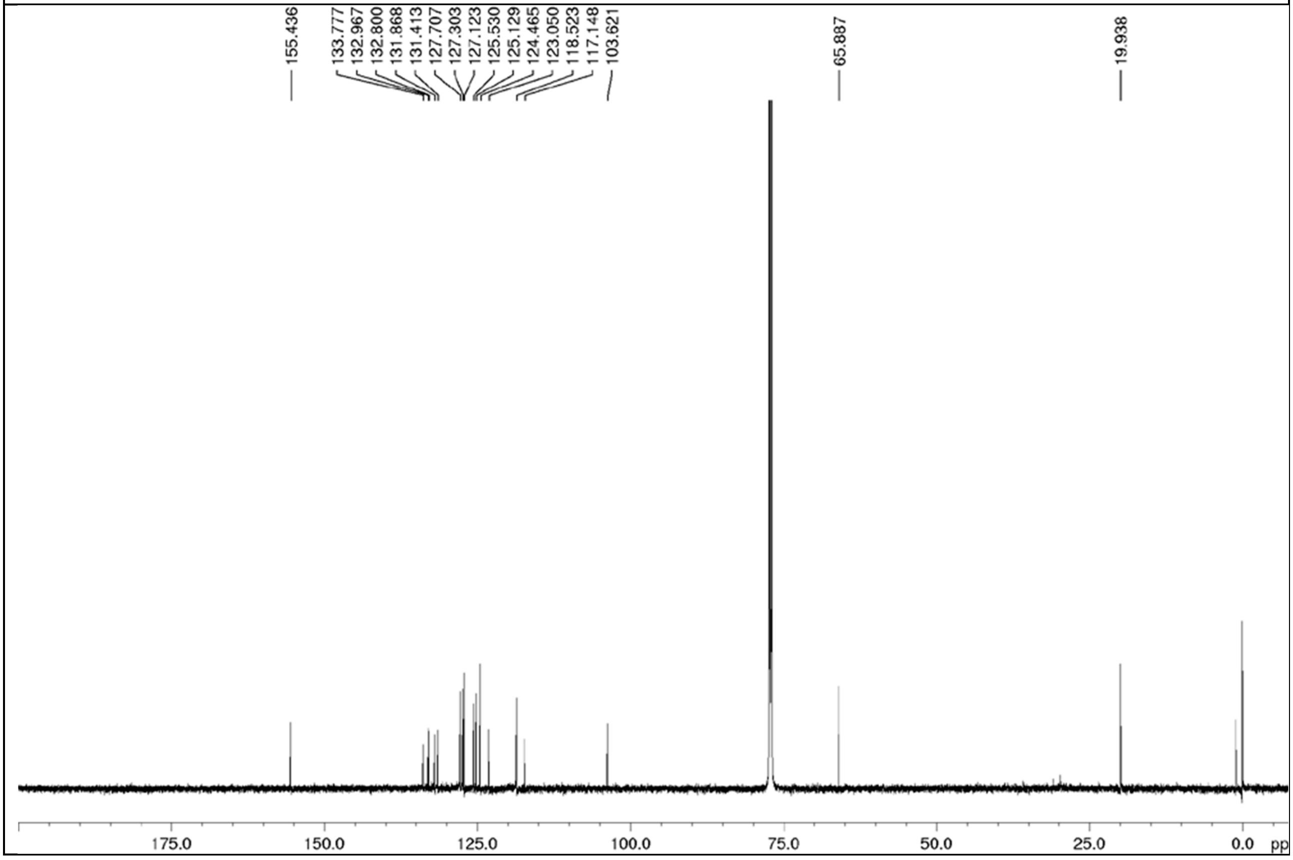

Figure S12. ^{1}H NMR and ^{13}C NMR spectra of compound **1b (*syn*)** at +25 °C.

^{1}H NMR (600 MHz), $CDCl_3$ – T = 298K

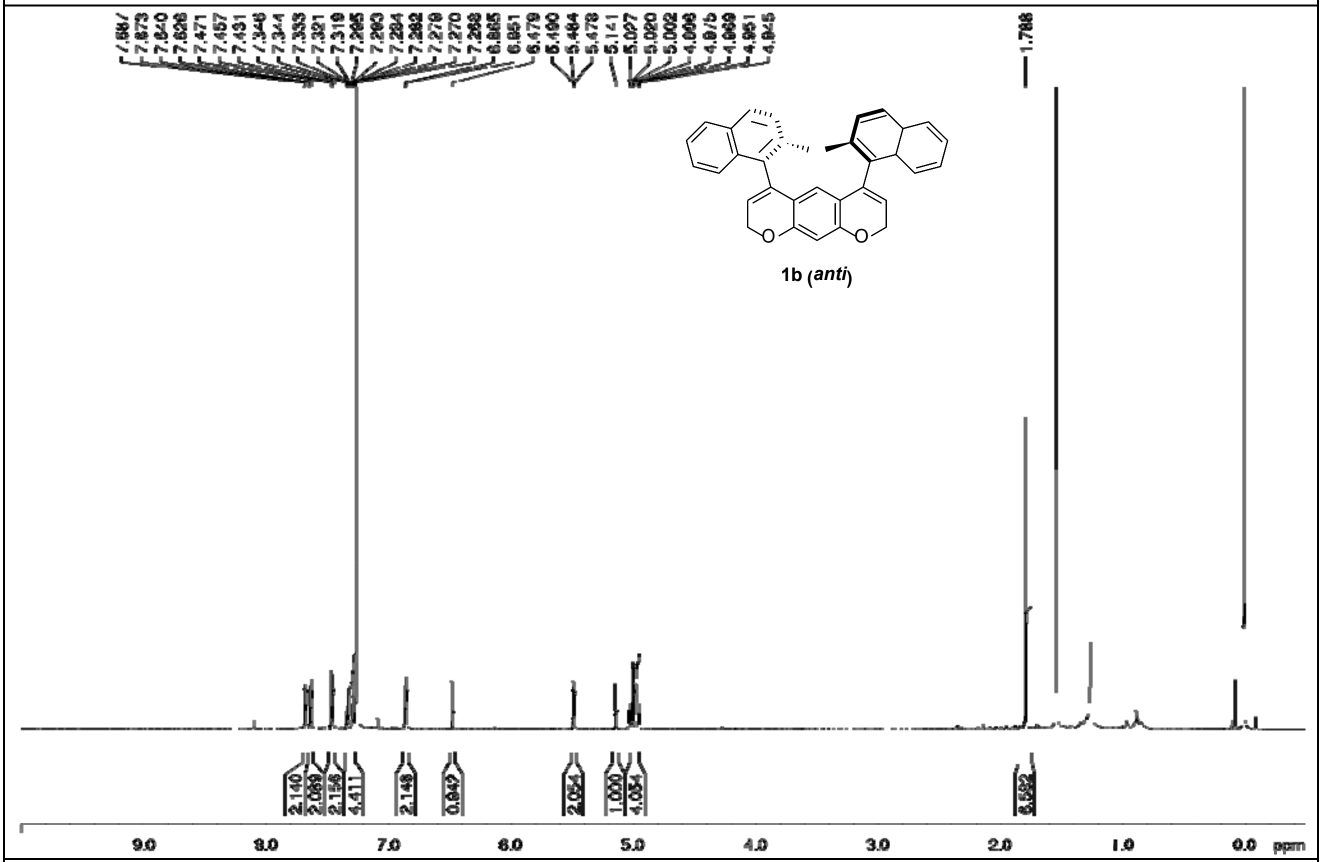

^{13}C NMR (150 MHz), $CDCl_3$ – T = 298K

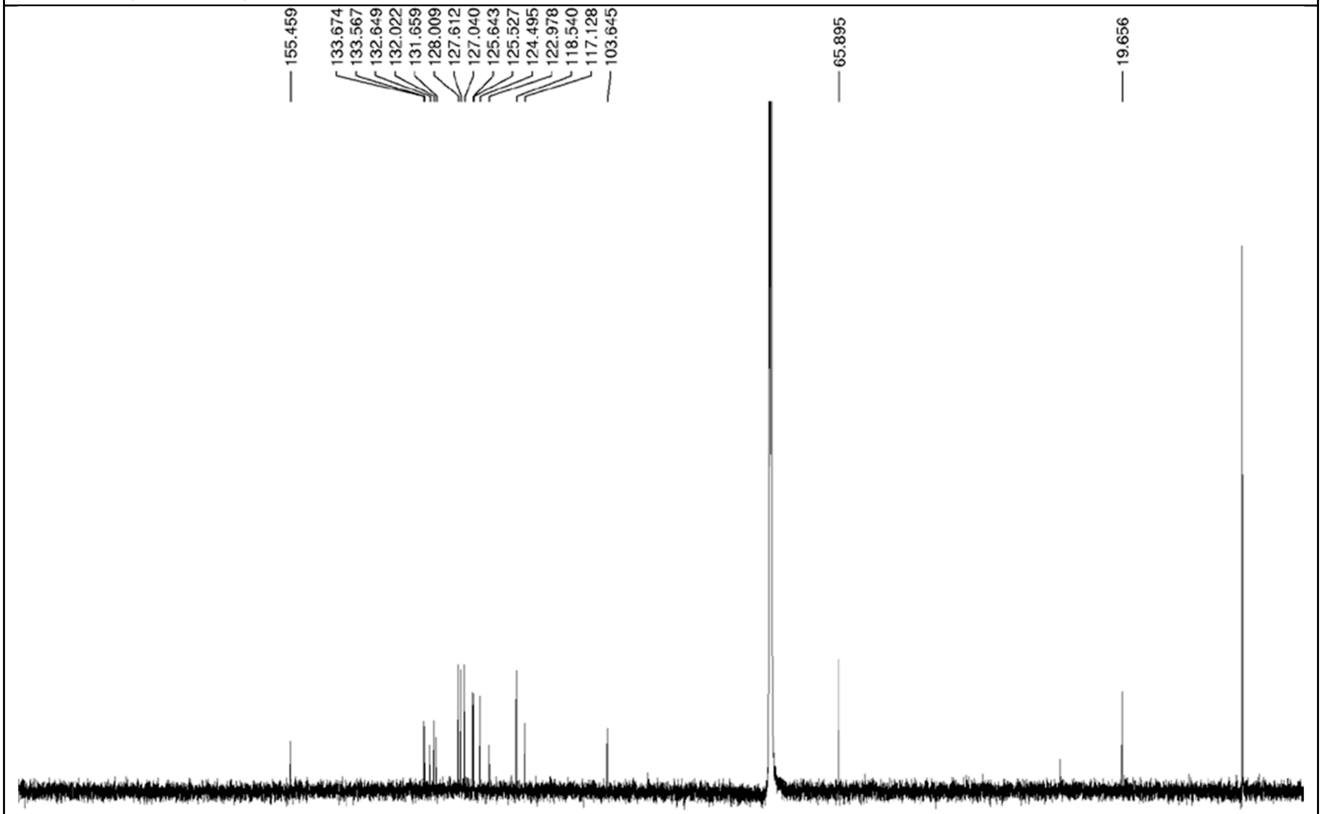

Figure S13. ^{1}H NMR and ^{13}C NMR spectra of compound **1b (*anti*)** at +25 °C.

6.1 DFT Calculations of *syn*/*anti* for compounds 1b

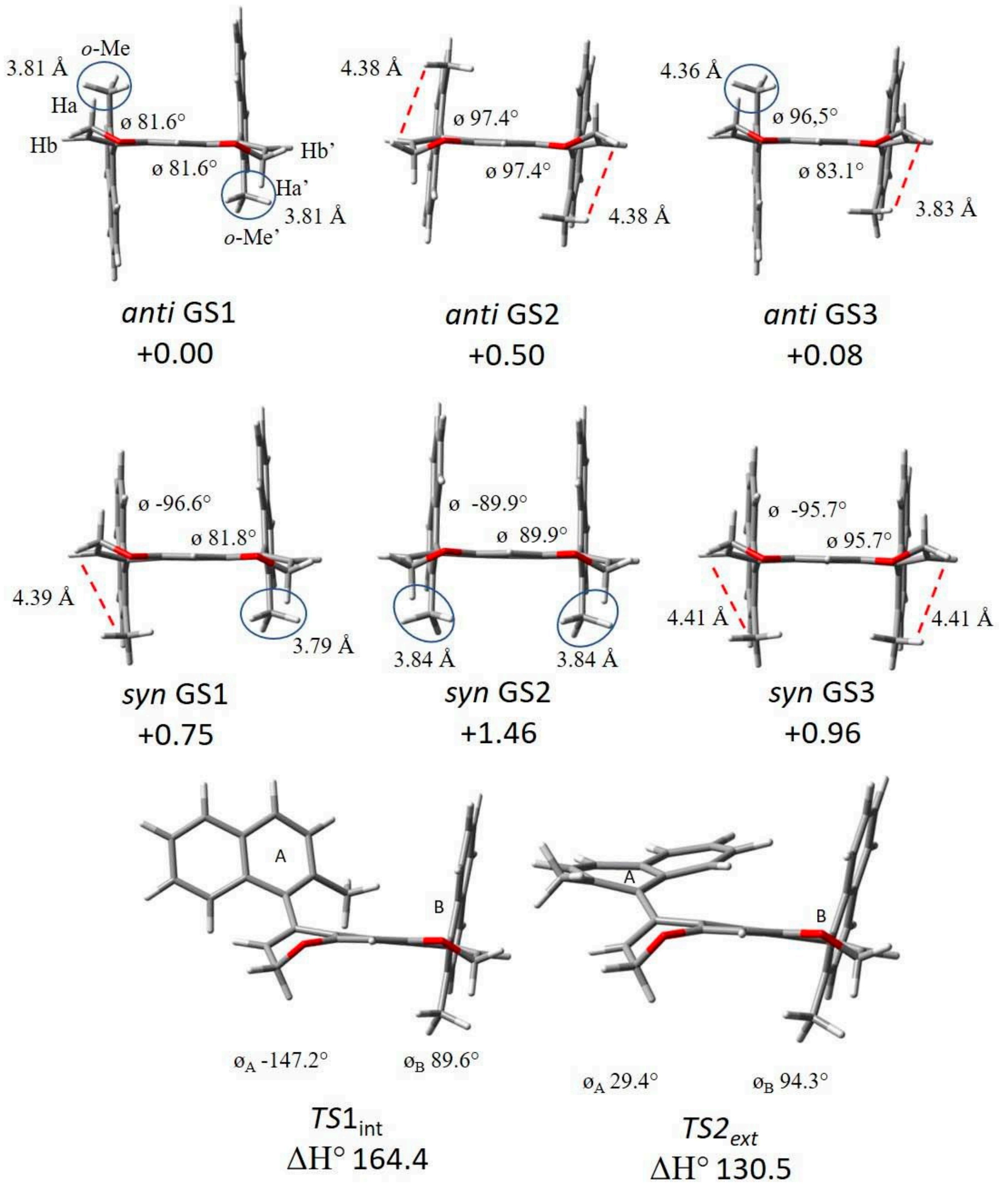

Figure S14. Predicted DFT (6-31+G(d,p), PCM=chloroform) conformations of *syn*/*anti* for compounds **1b** are shown. The relative Enthalpy energies are reported in kJ/mol.

Table S2. Descriptors are shown: r = atom distance; ø = dihedral angle

1b	r (H_a - *oMe*)	r (H_b - *oMe*)	r ($H_{a'}$ - *oMe'*)	r ($H_{b'}$ - *oMe'*)	$ø_A$	$ø_B$	% *pop*
GS1 *anti*	3.81	5.03	3.81	5.03	81.60	81.60	20.10
GS2 *anti*	5.17	4.38	5.17	4.38	97.40	97.40	17.02
GS3 *anti*	4.36	5.16	5.00	3.83	96.50	83.10	19.31
GS1 *syn*	5.19	4.39	3.79	5.01	-96.60	81.80	15.80
GS2 *syn*	3.84	5.00	3.84	5.00	-89.90	89.90	12.90
GS3 *syn*	5.22	4.41	5.22	4.41	-95.74	95.71	14.80
avg. *anti vs syn*	4.40 vs 4.80	4.87 vs 4.58	4.63 vs 4.29	4.42 vs 4.80	91.5 vs -94.3	86.9 vs 88.1	56/44

6.2 DFT Calculations of *syn*/*anti* for compounds 2b

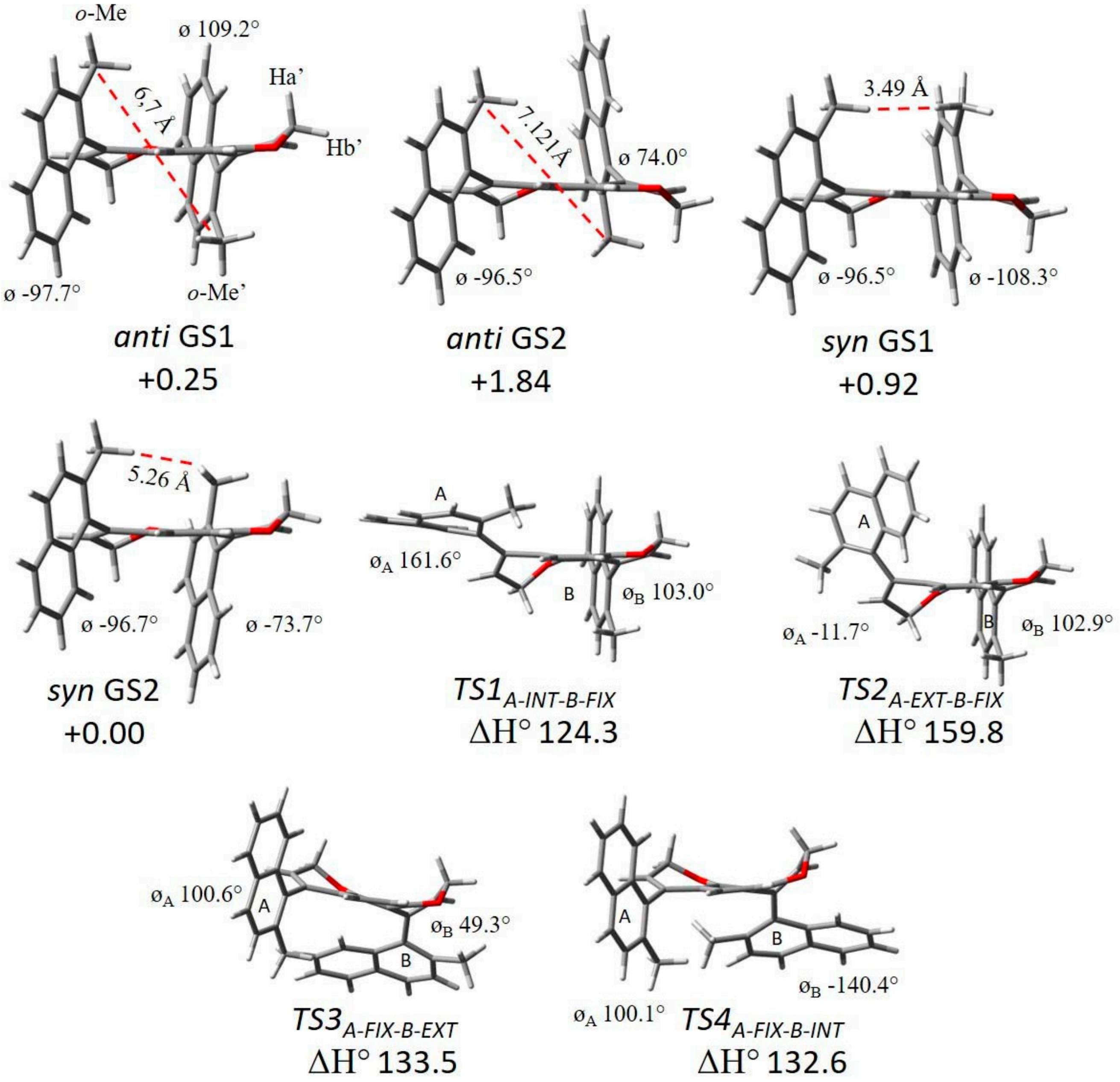

Figure S15. Predicted DFT (6-31+G(d,p), PCM=chloroform) conformations of *syn*/*anti* for compounds **2b** are shown. The relative Enthalpy energies are reported in kJ/mol.

Table S3. Descriptors are shown: r = atom distance; ø = dihedral angle

2b	r (oMe - *oMe′*)	$ø_A$	$ø_B$	% *pop*
GS1 *anti*	6.70	-97.70	109.20	28.96
GS2 *anti*	7.11	-96.50	74.00	14.65
GS1 *syn*	3.49	-96.50	-108.30	22.55
GS2 *syn*	5.26	-96.70	-73.70	33.83
avg. *anti vs syn*	7.17 vs 4.53	-96.8 vs -96.2	97.0 vs -87.8	44/56

6.3 Kinetic Study at 120 °C – ^{1}HNMR

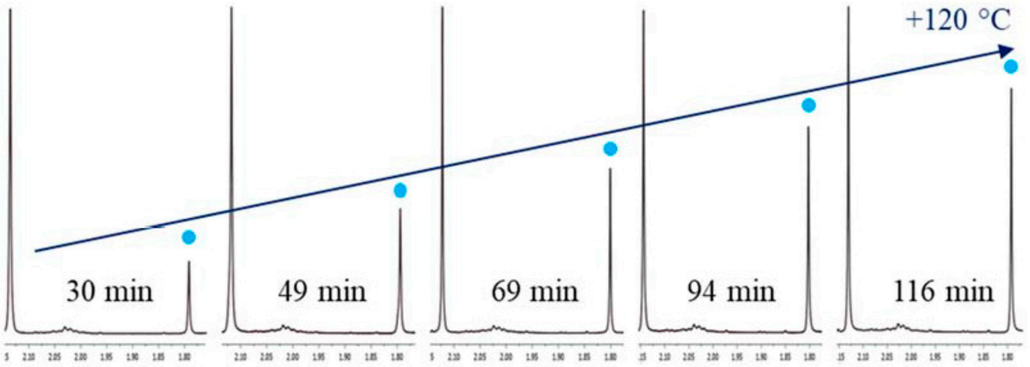

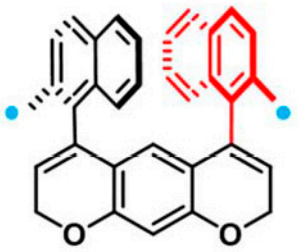

1b anti

120 °C

t (s)	ln (Cx - Cxeq)
0	-0.7985077
1800	-1.27296568
2940	-1.66073121
4140	-2.04022083
5640	-2.40794561
6960	-2.81341072

$\Delta G^{\ddagger}_{120°C}$ = 129.3 kJ/mol

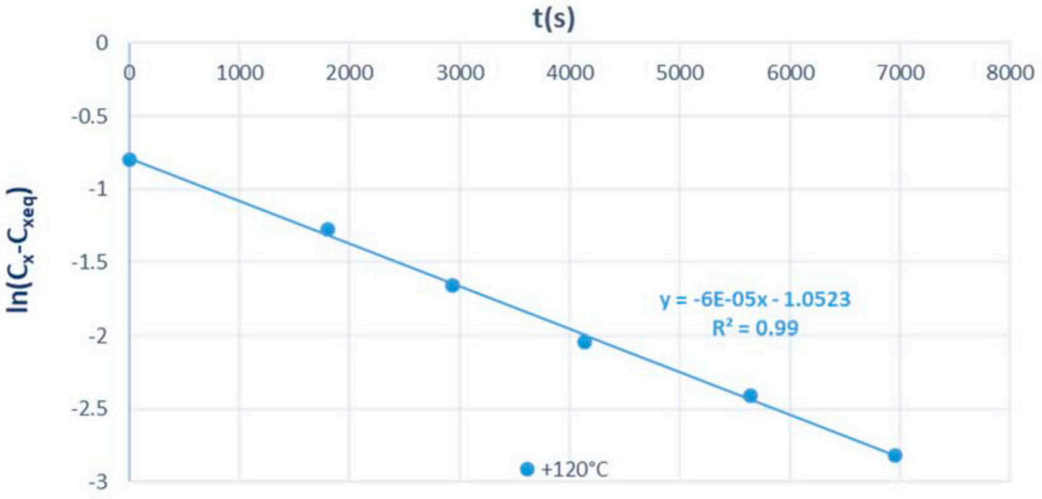

Figure S16. Kinetic studies of reversible first-order diastereomerization of **1b**. A sample of the first eluted atropisomer was heated in tube NMR at +120 °C (bath oil) constant temperature in 1,1,2,2-tetrachloroethane-d2. After cooling at room temperature, 1H NMR was acquired at different times and analyzed the integrals of methyl signals to measure the atropisomeric diastereomerization. Xa molar fraction of first eluted atropisomer. Xa_{eq} molar fraction at equilibrium.

6.4 Kinetic off-column of syn/anti interconversion of 1b

Analytical conditions: (*S,S*)-Whelk-O2 10 micron (250*4.6 mm L*ID) by using *n*-hexane/dichloromethane 95/5 + 0.1% ethanol at flow rate of 1.0 mL/min. Detector UV 268 nm.

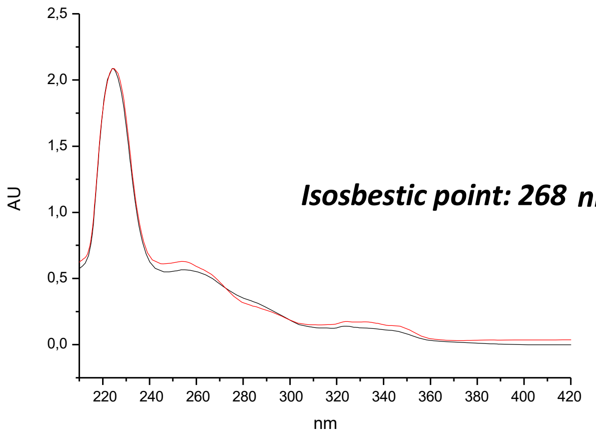

Figure S17. Overlapped UV spectra of two *syn/anti* isomers after separation at the same concentration.

Table S4. Data related to kinetic at different temperatures.

**T** (°C)	$\mathbf{K_{eq}}$	**k** (min^{-1})	$\mathbf{k_{-1}}$(min^{-1})	**ΔG** (Kcal/mol)	**ΔG** (KJ/mol)
110	0,996	0,00139	0,00140	30,74	128,61
120	0,996	0,00309	0,00310	30,94	129,45
130	0,996	0,00808	0,00811	30,98	129,61

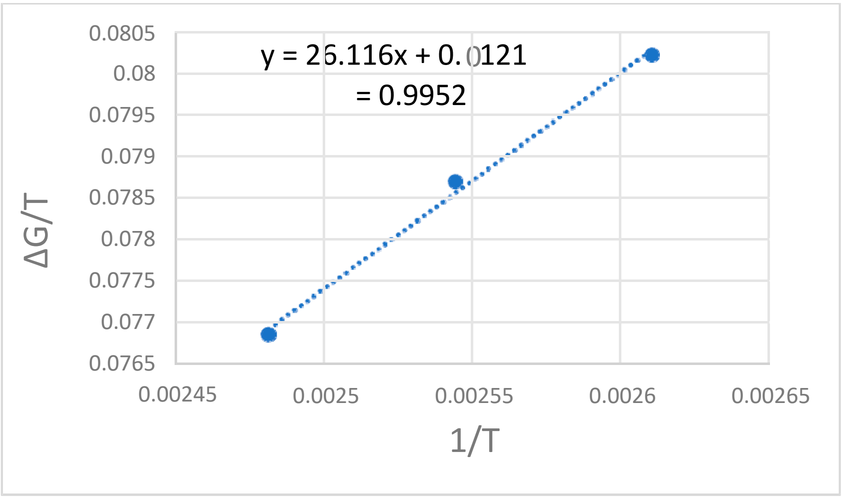

Figure S18: Eyring plot of Kinetic off-column of *syn/anti* interconversion of **1b**.

6.4.1 Kinetic study at 110 °C

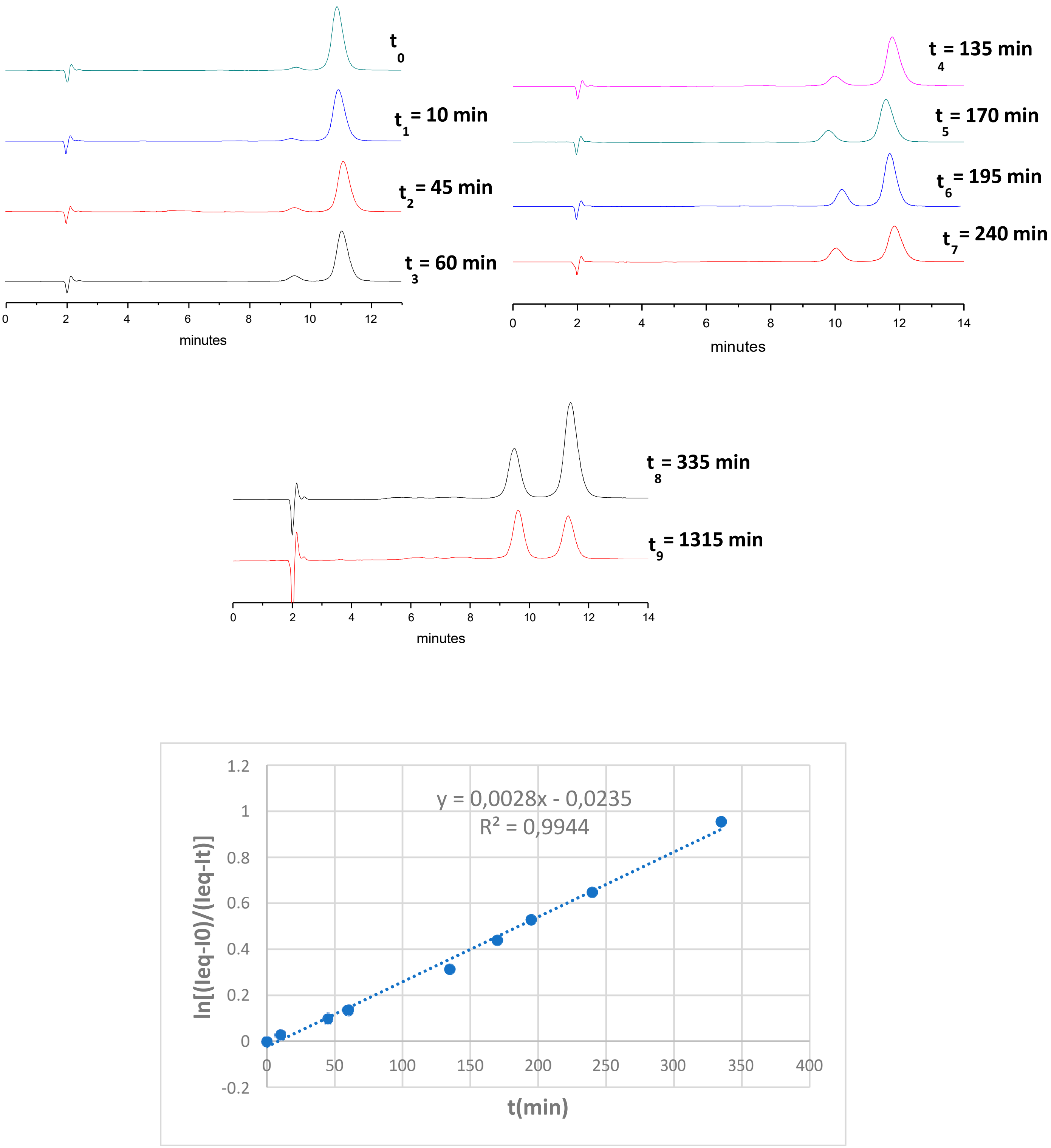

Figure S19. Kinetic studies of reversible first-order diastereomerization of **1b**. A sample of the second eluted atropisomer was heated in tube NMR at +110 °C (bath oil) constant temperature in cis/trans decaline. After the time reported the sample was cooled at room temperature and analyzed by HPLC.

Kinetic study at 120 °C

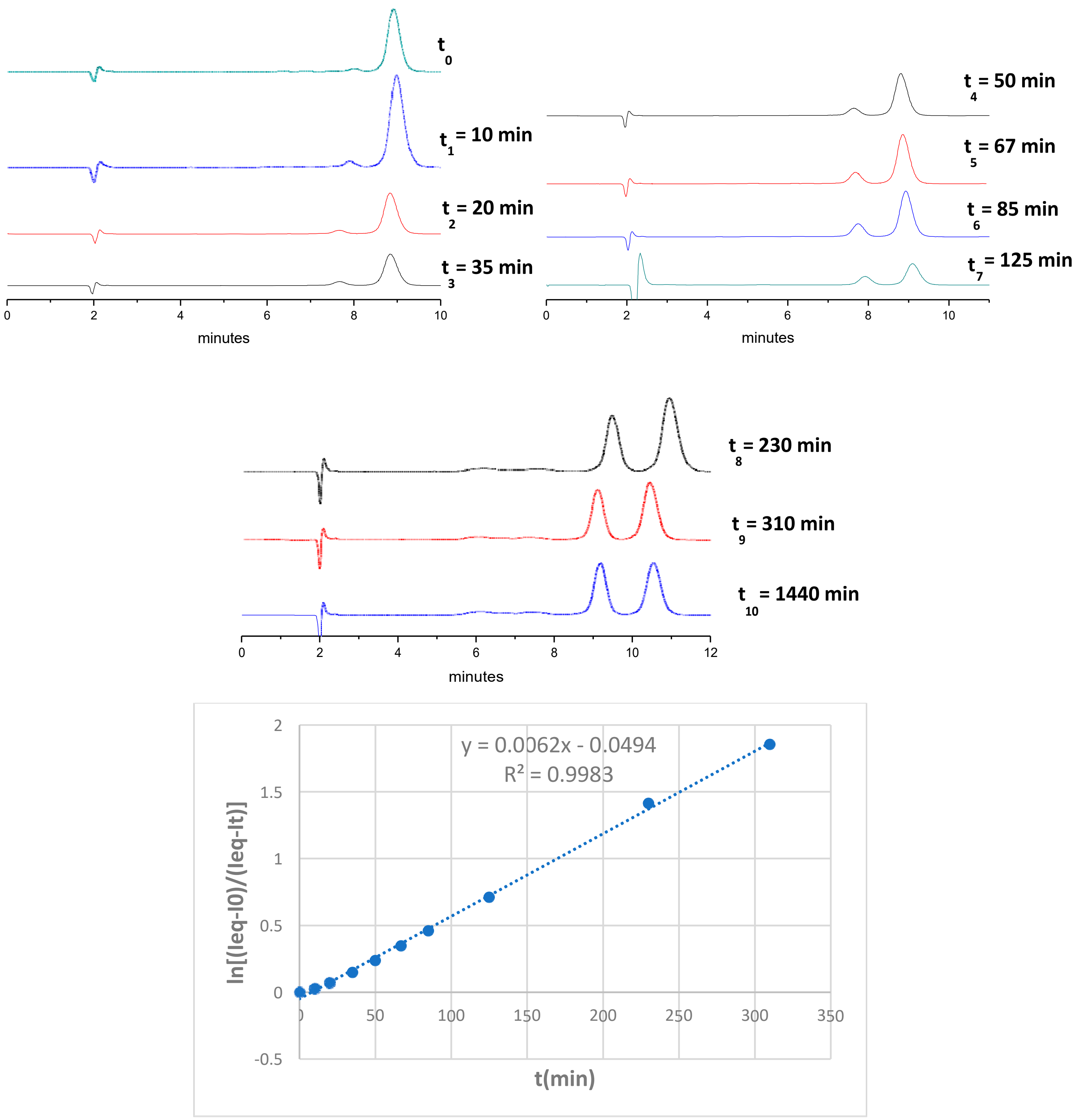

Figure S20. Kinetic studies of reversible first-order diastereomerization of **1b**. A sample of the second eluted atropisomer was heated in tube NMR at +120 °C (bath oil) constant temperature in cis/trans decaline. After the time reported the sample was cooled at room temperature and analyzed by HPLC.

6.4.2 Kinetic study at 130 °C

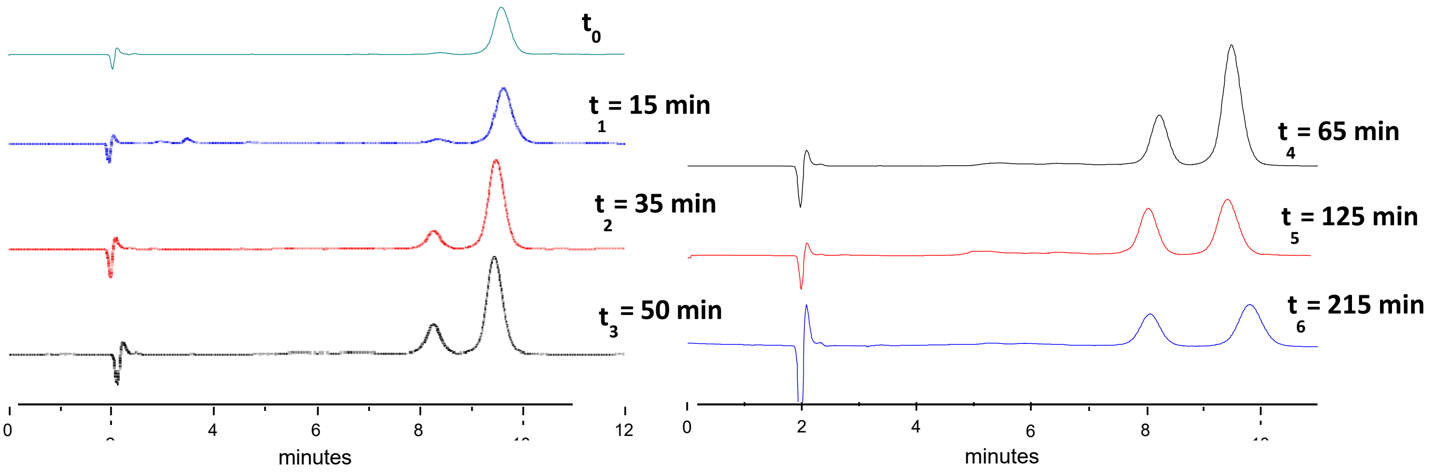

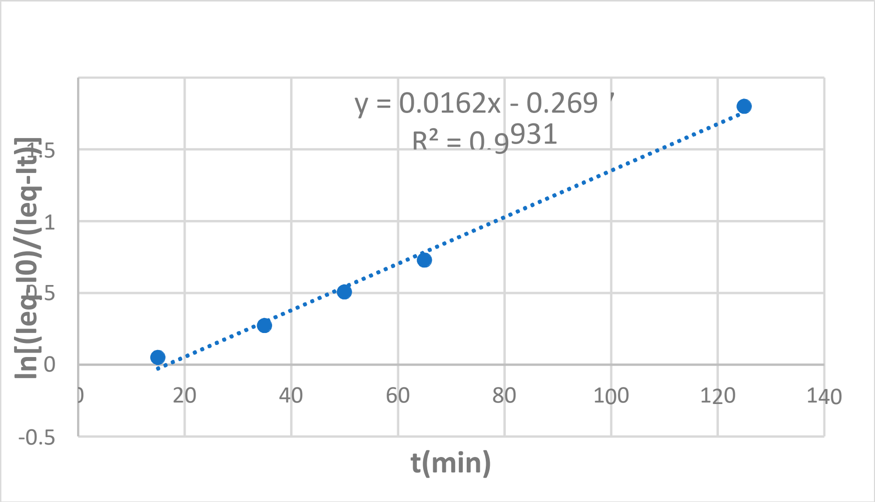

Figure S21. Kinetic studies of reversible first-order diastereomerization of **1b**. A sample of the second eluted atropisomer was heated in tube NMR at +110 °C (bath oil) constant temperature in cis/trans decaline. After the time reported the sample was cooled at room temperature and analyzed by HPLC.

6.5 ECD of 1b:

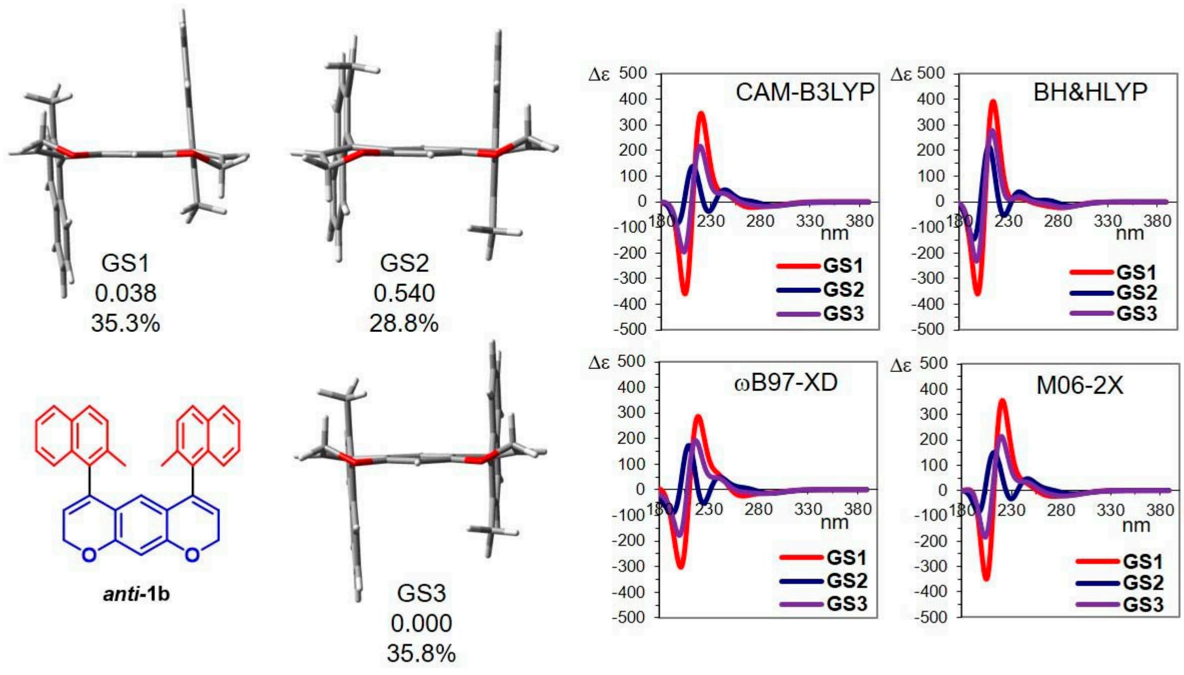

Figure S22. ECD studies of compound ***anti-1b***. In the Figure was reported all the simulated ECD spectra of GSs with the different functionals and same basis set 6-311++G(2d,p), PCM=acetonitrile. The sum of spectra was reported in the main text.

7. **Characterization of 2b**

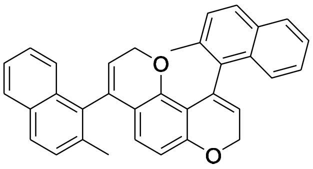

2b

Syn/Anti stereoisomers were separated by semipreparative CSP-HPLC on Chiralpak IB-N5 (250*10 mm L*ID) by using *n*-hexane/chloroform 85/15 at flow rate of 5.0 ml/min. Detector UV 254 nm. *Anti* and *syn* stereoisomers were assigned based on ^{1}H NMR and NOE effect.

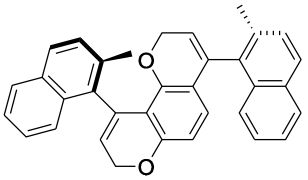

2b (*anti*)

2b (*anti*):^{1}H NMR (600 MHz) ($C_2D_2Cl_4$): δ (ppm) 7.78 – 7.76 (m, 2 H), 7.73 (d, *J* = 5.0 Hz, 1 H), 7.67 (d, *J* = 5.6 Hz, 2 H), 7.62 (d, *J* = 5.5 Hz, 1 H), 7.36 – 7.28 (m, 6 H), 7.26 – 7.24 (m, 2 H), 6.26 (d, *J* = 5.6 Hz, 1 H), 6.12 (d, *J* = 5.6 Hz, 1 H), 5.66 (t, *J* = 2.7 Hz, 1 H), 5.25 (, t, *J* = 2.5 Hz, 1 H), 5.25 (s, 1 H), 4.87 – 4.81 (m, 2 H), 3.96 (dd, J_1 = 9.8 Hz, J_2 = 2.6 Hz, 1 H), 3.66 (dd, J_1 = 9.8 Hz, J_2 = 2.4 Hz, 1 H), 2.39 (s, 3 H), 2.16 (s, 3 H); ^{13}C{^{1}H} NMR (150 MHz) ($C_2D_2Cl_4$): δ (ppm) 155.6, 151.2, 137.4, 134.2, 134.0, 133.8, 132.7, 132.63, 132.60, 132.5, 131.9, 131.8, 128.8, 128.7, 128.0, 127.9, 127.6, 126.6, 126.2, 126.0, 125.9, 125.8, 125.6, 125.1, 124.6, 122.3, 119.7, 118.7, 113.4, 109.1, 65.3, 64.7, 21.0, 20.4.

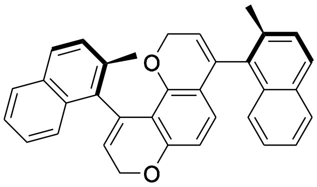

2b (*syn*)

2b (*syn*):^{1}H NMR (600 MHz) ($C_2D_2Cl_4$): δ (ppm) 7.81 – 7.76 (m, 2 H), 7.72 (d, *J* = 5.3 Hz, 1 H), 7.67 (d, *J* = 5.5 Hz, 2 H), 7.55 (d, *J* = 5.6 Hz, 1 H), 7.38 – 7.35 (m, 2 H), 7.33 – 7.29 (m, 3 H), 7.26 – 7.24 (m, 2 H), 6.26 (d, *J* = 5.6 Hz, 1 H), 6.12 (d, *J* = 5.6 Hz, 1 H), 5.66 (t, *J* = 2.7 Hz, 1 H), 5.26 (, t, *J* = 2.5 Hz, 1 H), 4.87 – 4.81 (m, 2 H), 3.94 (dd, J_1 = 9.8 Hz, J_2 = 2.6 Hz, 1 H), 3.73 (dd, J_1 = 9.8 Hz, J_2 = 2.5 Hz, 1 H), 2.35 (s, 3 H), 2.21 (s, 3 H); ^{13}C{^{1}H} NMR (150 MHz) ($C_2D_2Cl_4$): δ (ppm) 155.6, 151.2, 137.4, 134.1, 134.0, 133.8, 132.7, 132.6, 132.5, 131.9, 131.8, 128.8, 128.6, 128.0, 127.9, 127.6, 126.6, 126.2, 126.0, 125.9, 125.6, 125.1, 124.6, 122.3, 119.7, 118.7, 113.4, 109.1, 65.3, 64.7, 20.9, 20.4.

^{1}H NMR (600 MHz), $C_2D_2Cl_4$ – T = 298K

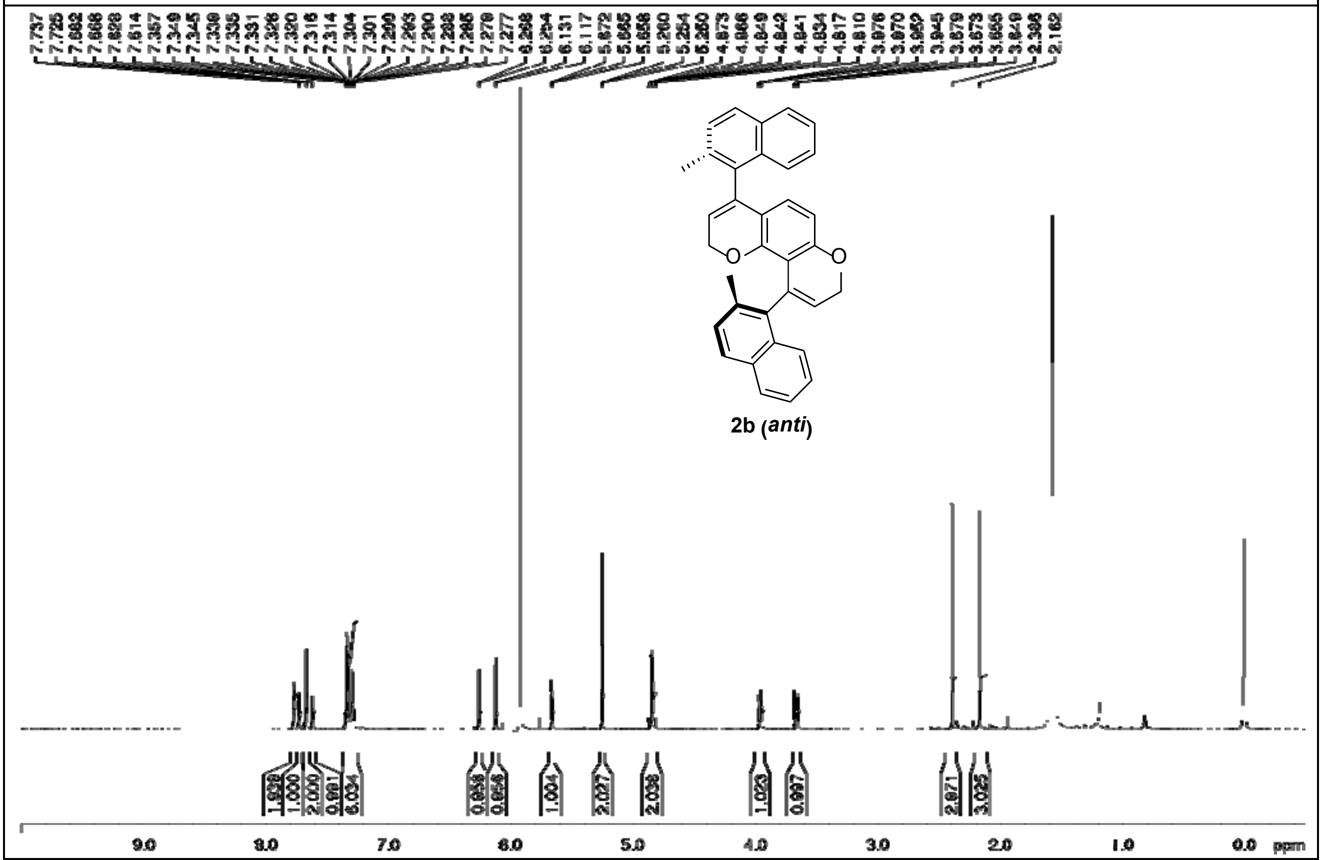

^{13}C NMR (150 MHz), $C_2D_2Cl_4$ – T = 298K

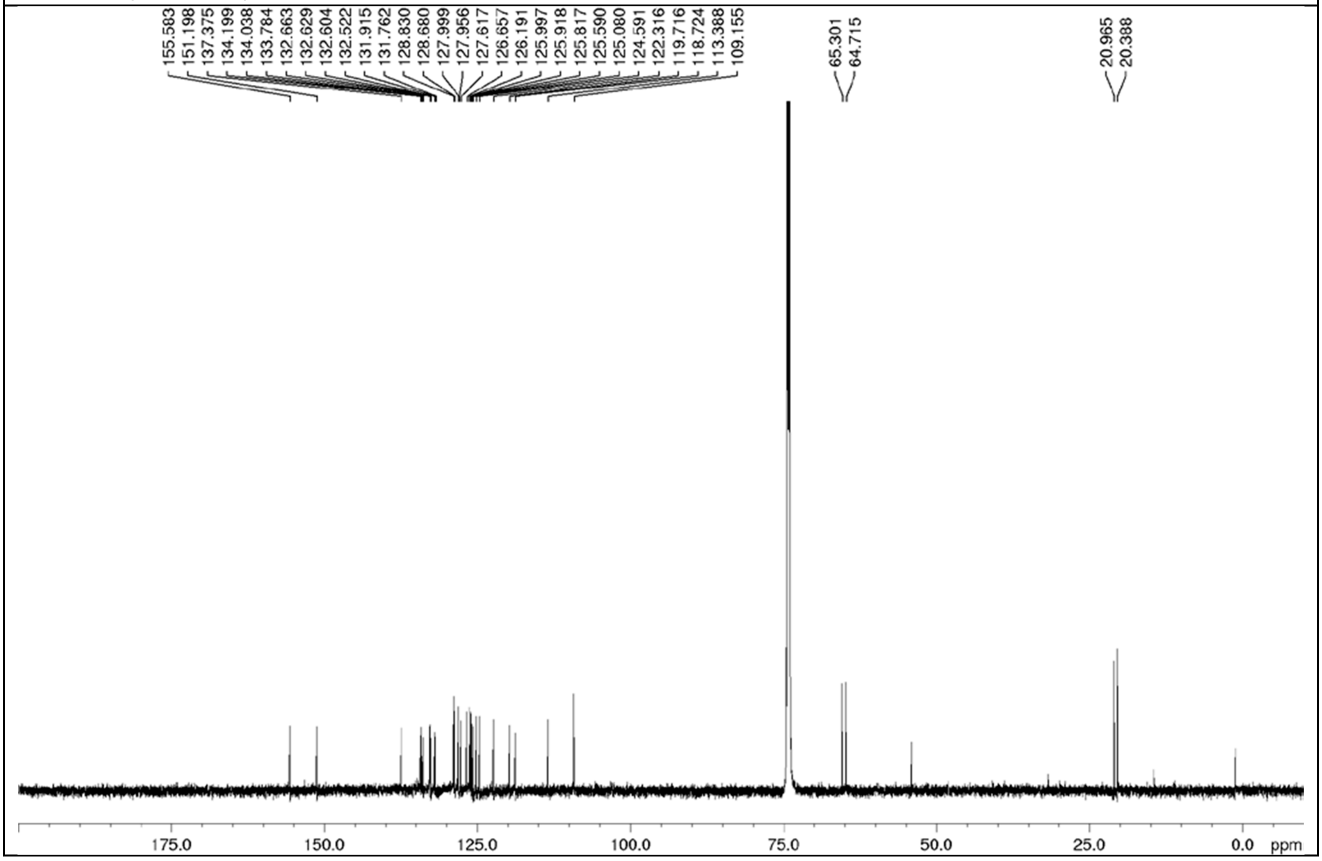

Figure S23. ^{1}H NMR and ^{13}C NMR spectra of compound **2b (*anti*)** at +25 °C.

^{1}H NMR (400.13 MHz), $C_2D_2Cl_4$ – T = 298K

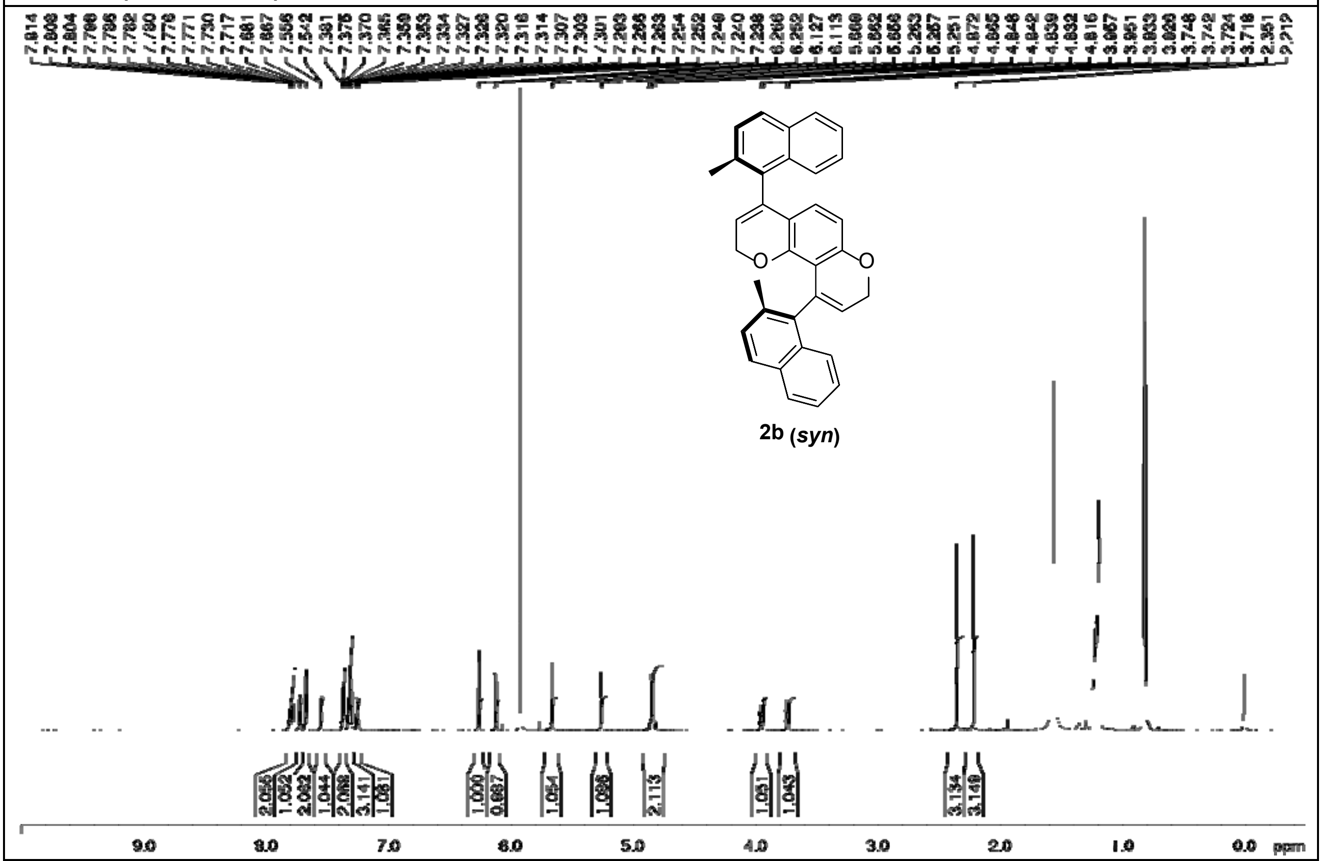

^{13}C NMR (100.6 MHz), $C_2D_2Cl_4$ – T = 298K

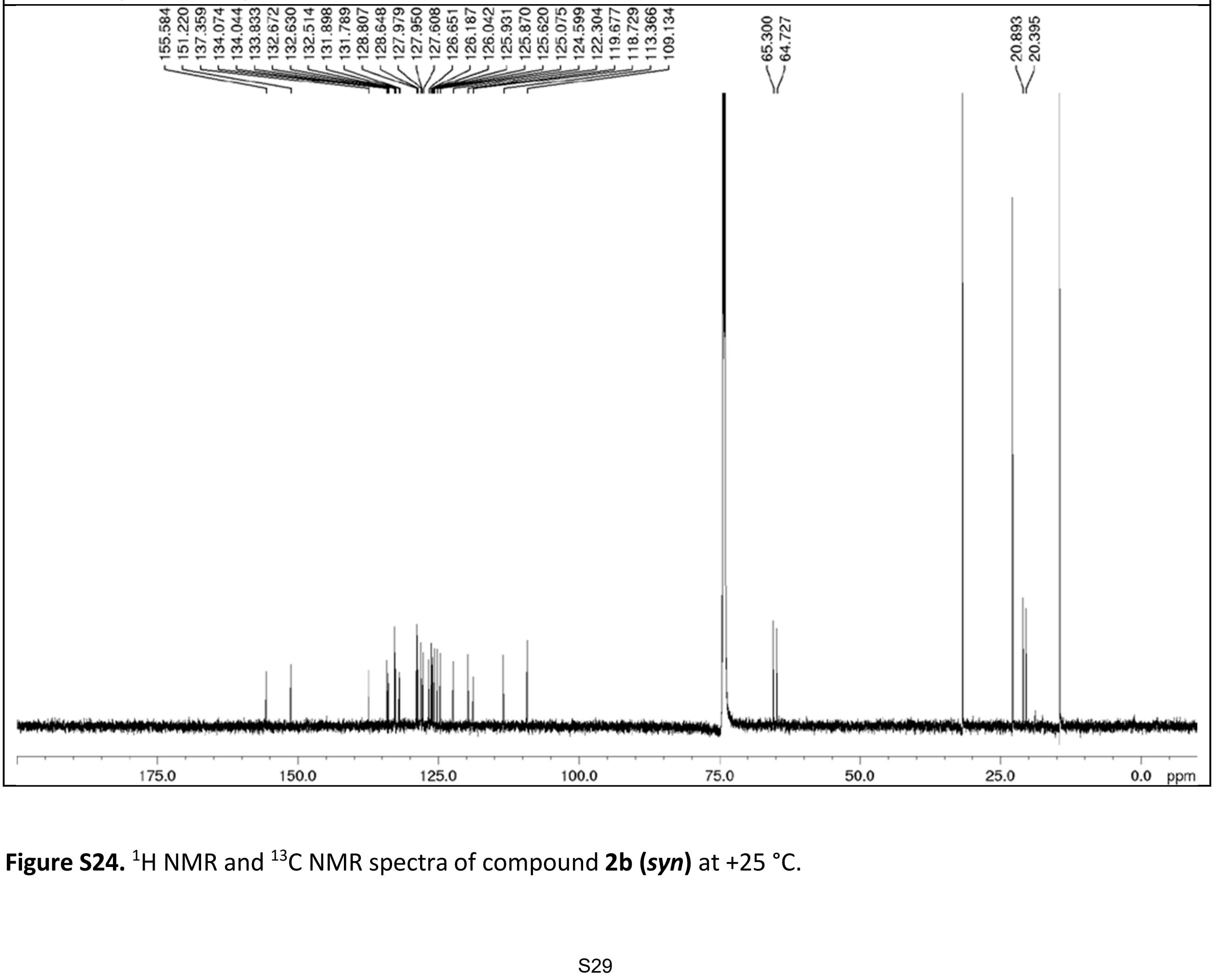

Figure S24. ^{1}H NMR and ^{13}C NMR spectra of compound **2b (*syn*)** at +25 °C.

7.1 Kinetic Study at 102 °C and 120°C - ^{1}HNMR

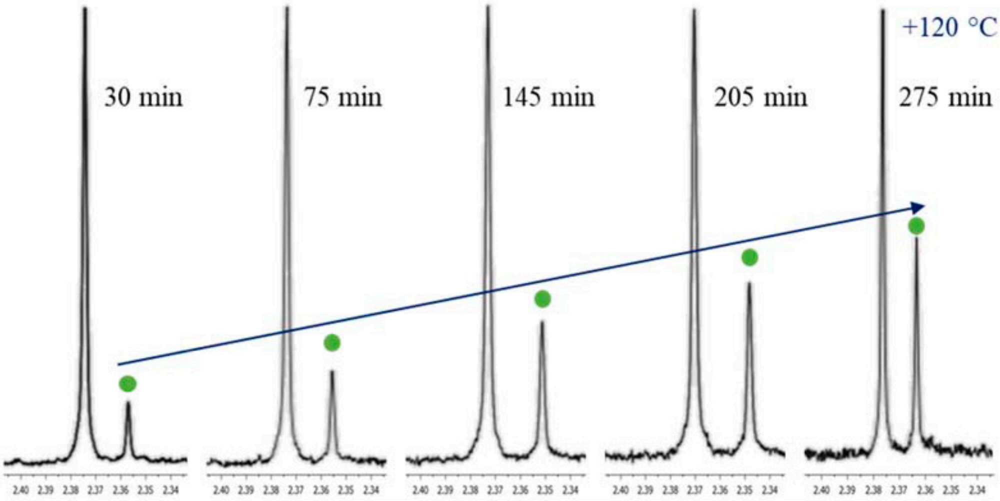

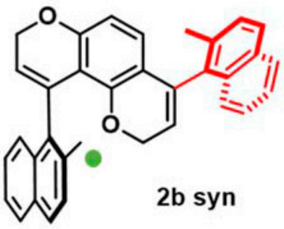

2b syn

102 °C			120 °C	
t (s)	ln (Cx - Cxeq)		t (s)	ln (Cx - Cxeq)
0	-0.88673193		0	-1.016111067
4500	-0.944175935		1800	-1.164752091
9000	-1.007036345		4500	-1.378326191
18000	-1.123007446		8700	-1.532476871
28500	-1.210661792		12300	-1.814005078
46500	-1.376740148		16500	-2.017406151
$\Delta G^{\ddagger}_{102°C}$	133.0 kJ/mol		$\Delta G^{\ddagger}_{120°C}$	133.9 kJ/mol

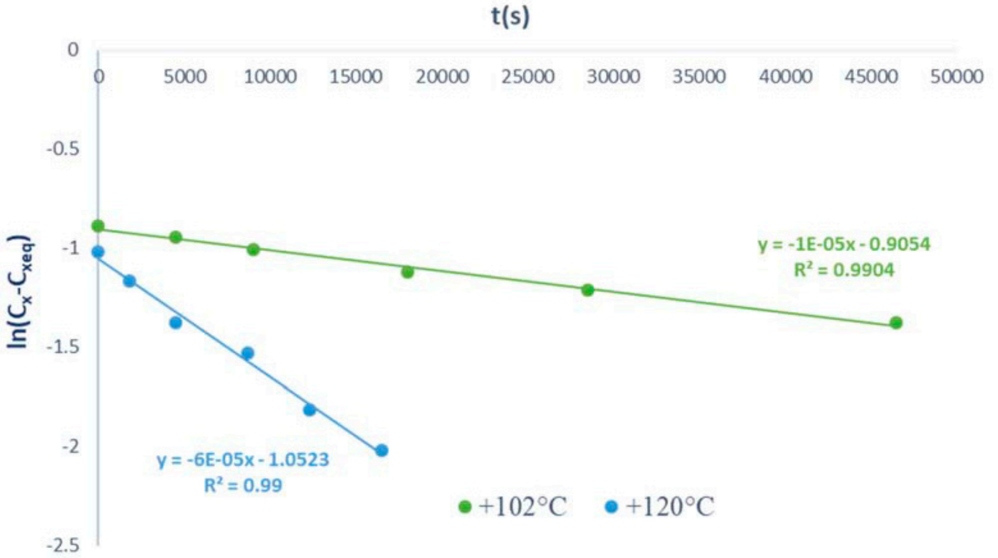

Figure S25. Kinetic studies of reversible first-order diastereomerization of **2b**. A sample of the first eluted atropisomer was heated in tube NMR at +102 °C and +120°C (bath oil) constant temperature in 1,1,2,2-tetrachloroethane-d2. After cooling at room temperature, 1H NMR was acquired at different times and analyzed the integrals of methyl signals to measure the atropisomeric diastereomerization. Xa molar fraction of first eluted atropisomer. Xaeq molar fraction at equilibrium.

7.2 CSP separation and ECD of 2b:

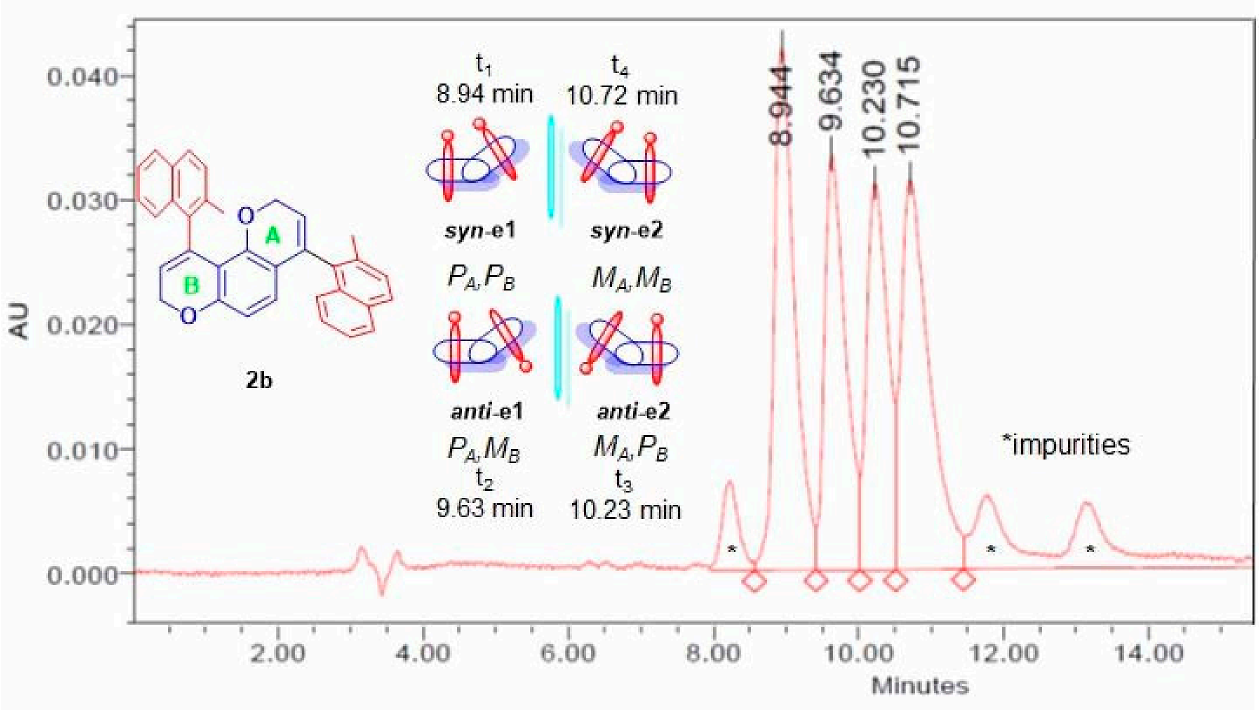

Figure S26. CSP-HPLC separation of compound **2b**. Chiralpak IB-N5 column: 5 mm 10 mm I.D. x 250 mm L; eluent 85:15 *n*-hexane/chloroform.

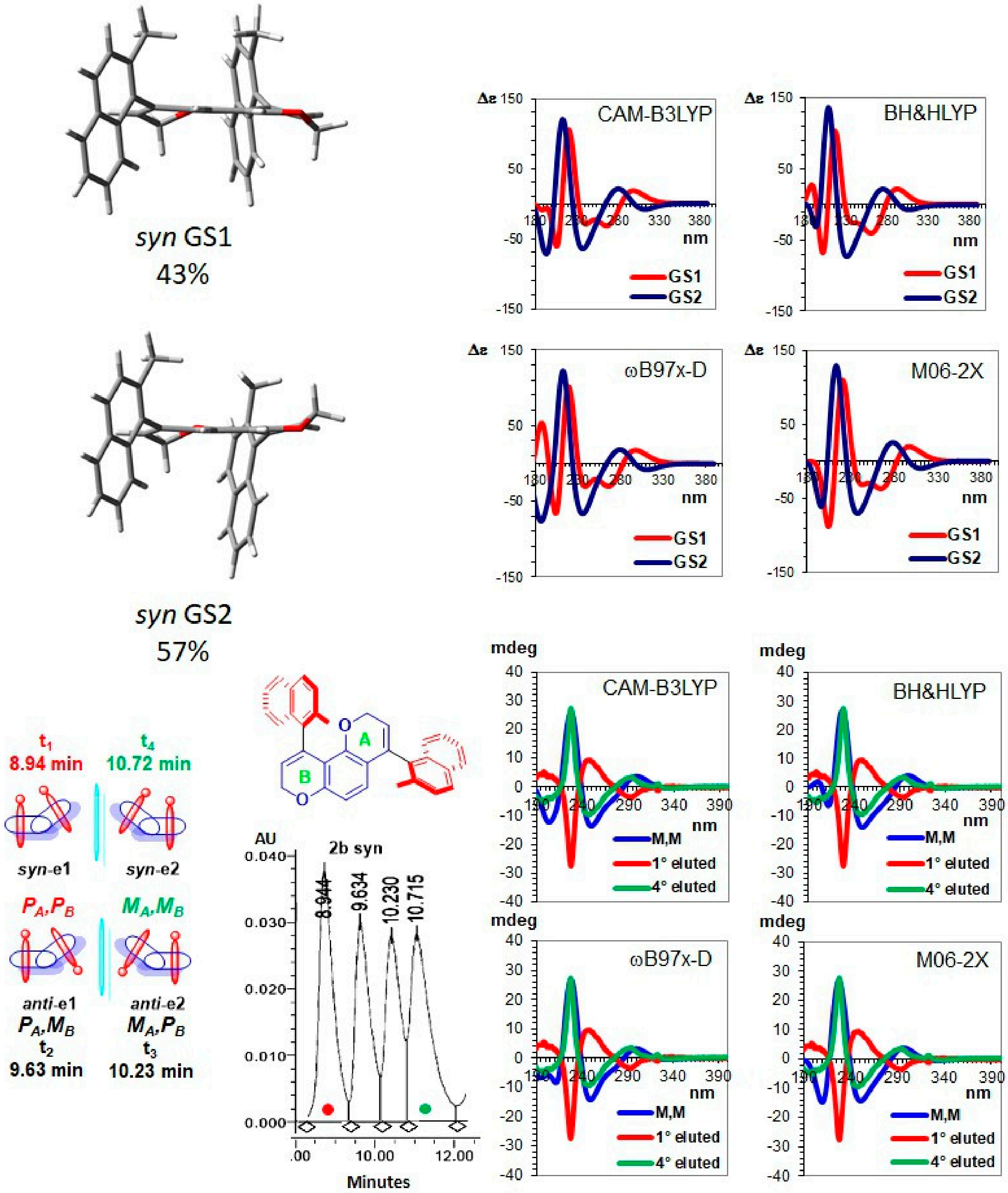

Figure S27. ECD studies of compound ***syn-2b***. In the Figure was reported all the simulated ECD spectra of GSs with the different functionals and same basis set 6-311++G(2d,p) PCM=acetonitrile. Four different functionals and 6-311++G(2d,p) basis set were used. To obtain a beter overlap, the calculated spectra were red-shifted by 11 nm for CAM-B3LYP, 15 nm for BH&HLYP and 12 nm for ωB97x-D, and 10 nm for M06-2X while they were multiplied by a factor of 0.28 for all the functionals.

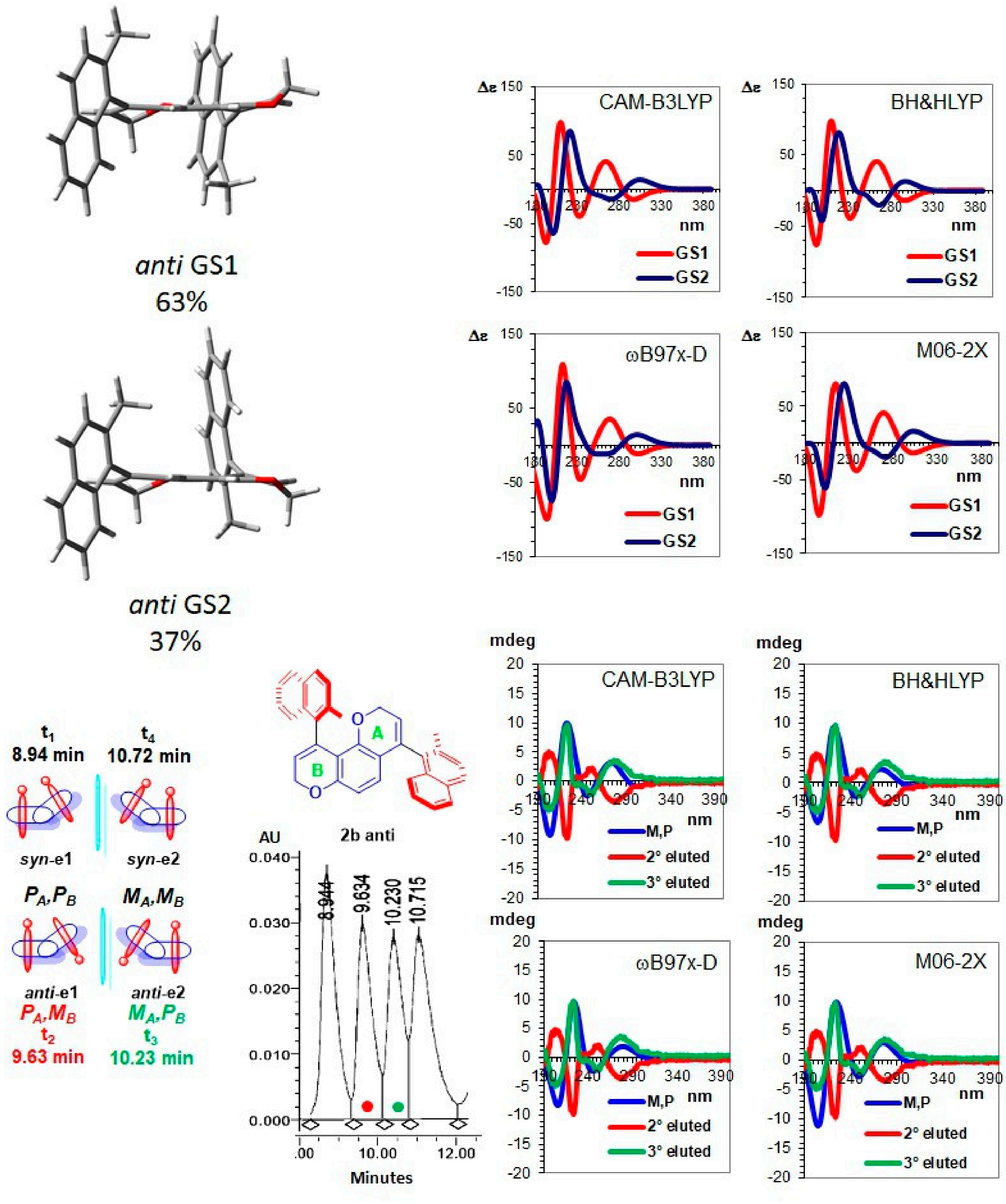

Figure S28. ECD studies of compound ***anti-2b***. In the Figure was reported all the simulated ECD spectra of GSs with the different functionals and same basis set 6-311++G(2d,p) PCM=acetonitrile. Four different functionals and 6-311++G(2d,p) basis set were used. To obtain a beter overlap, the calculated spectra were red-shifted by 10 nm for CAM-B3LYP, 11 nm for BH&HLYP, 9 nm for ωB97x-D, and 7 nm for M06-2X while they were multiplied by a factor of 0.15 for CAM-B3LYP and M06-2X, 0.12 for BH&HLYP and 0.10 for ωB97x-D.

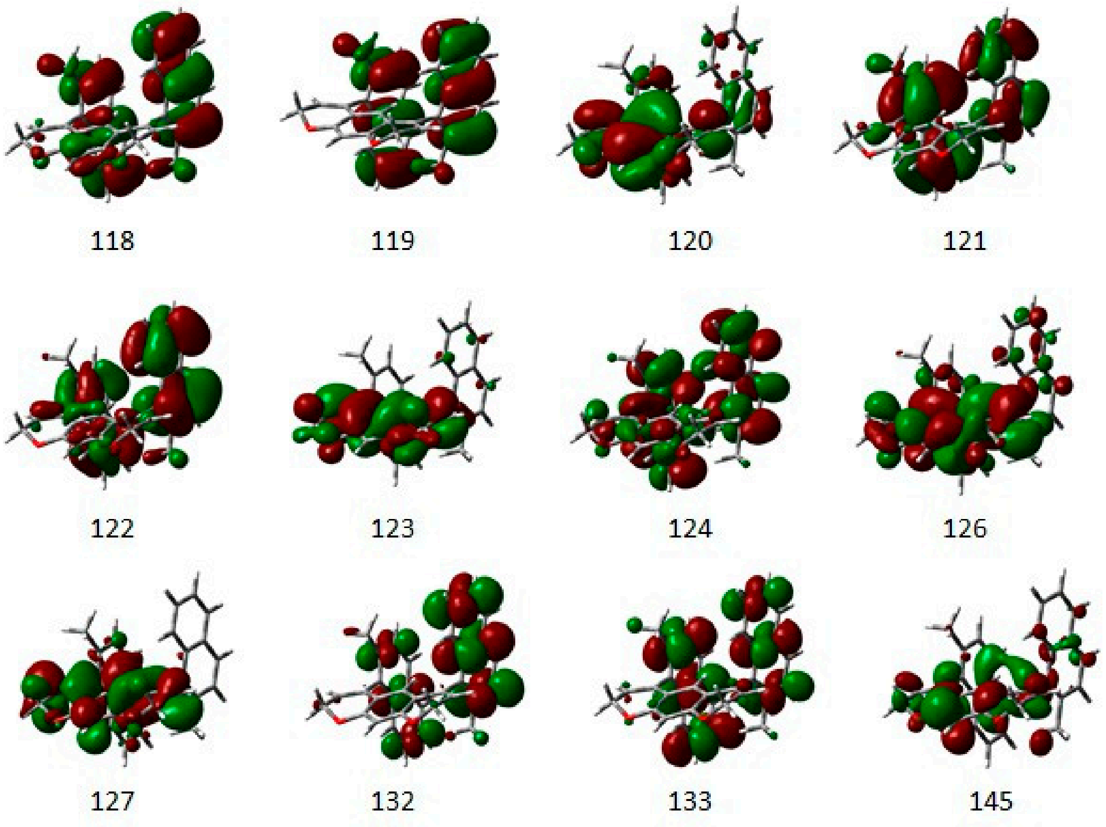

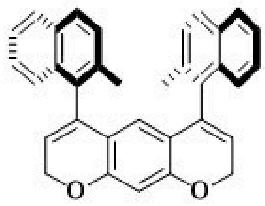

1b anti
GS3

Excited State	Wavelenght (nm)	HOMO	MO	LUMO	%
		123	->	127	12.3
9	243.37	120	->	126	17.7
		122	->	126	23.5
14	217.65	123	->	145	15.3
		120	->	126	13.4
20	211.34	122	->	124	12.1
		123	->	133	17.0
24	206.53	121	->	126	10.8
		119	->	133	20.7
33	200.42	119	->	126	16.8
		118	->	132	13.6

Figure S29. Homo-Lumo orbitals of best calculated GS3 *anti* conformation of compound **1b**.

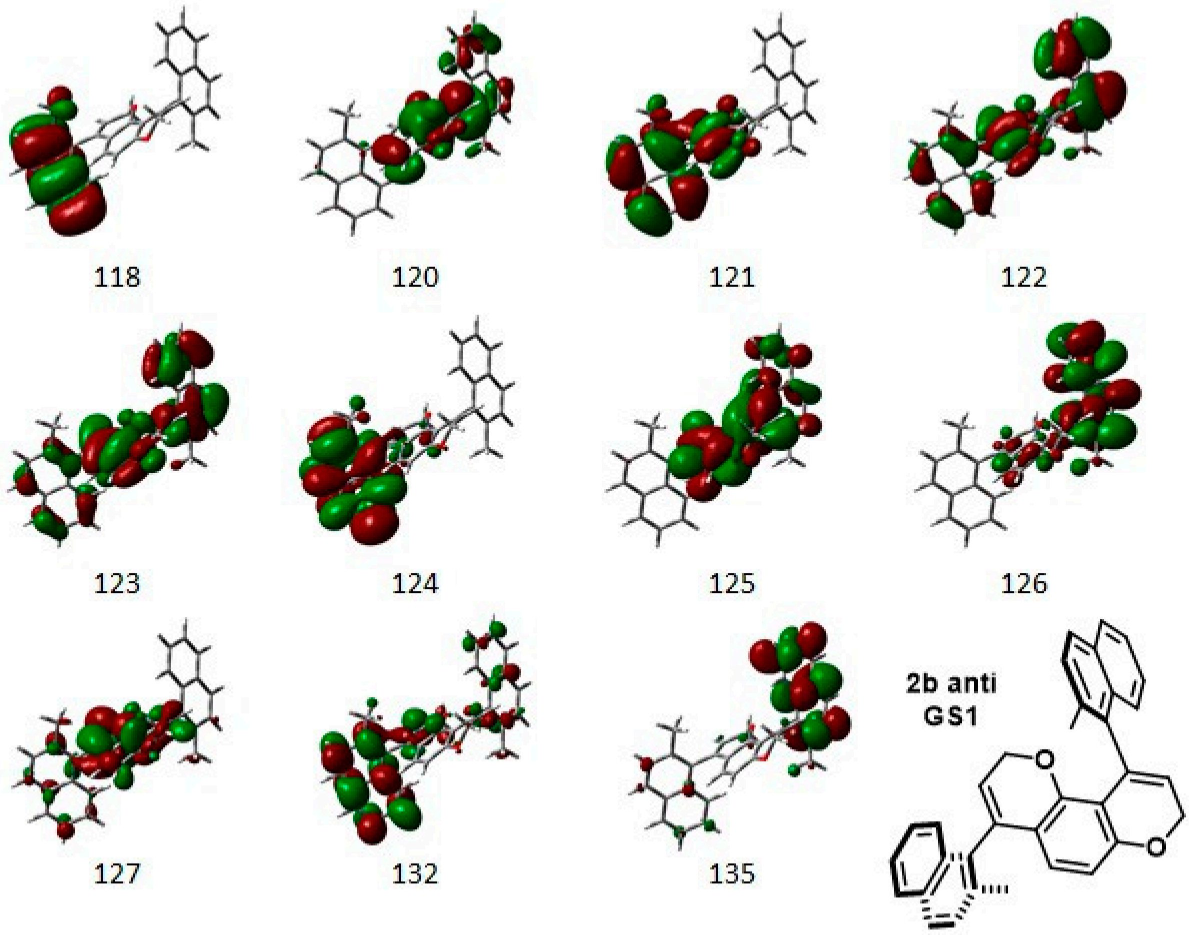

Excited State	Wavelenght (nm)	HOMO	MO	LUMO	%
		120	->	125	14.1
7	266.38	123	->	127	14.8
		120	->	127	19.6
13	233.6	122	->	127	27.6
31	208.82	121	->	126	37.3
		118	->	124	14.8
33	208.16	121	->	126	16.2
		121	->	132	14.7
		120	->	134	11.8
42	201.49	120	->	135	16.5

Figure S30. Homo-Lumo orbitals of best calculated GS1 *anti* conformation of compound **2b**.

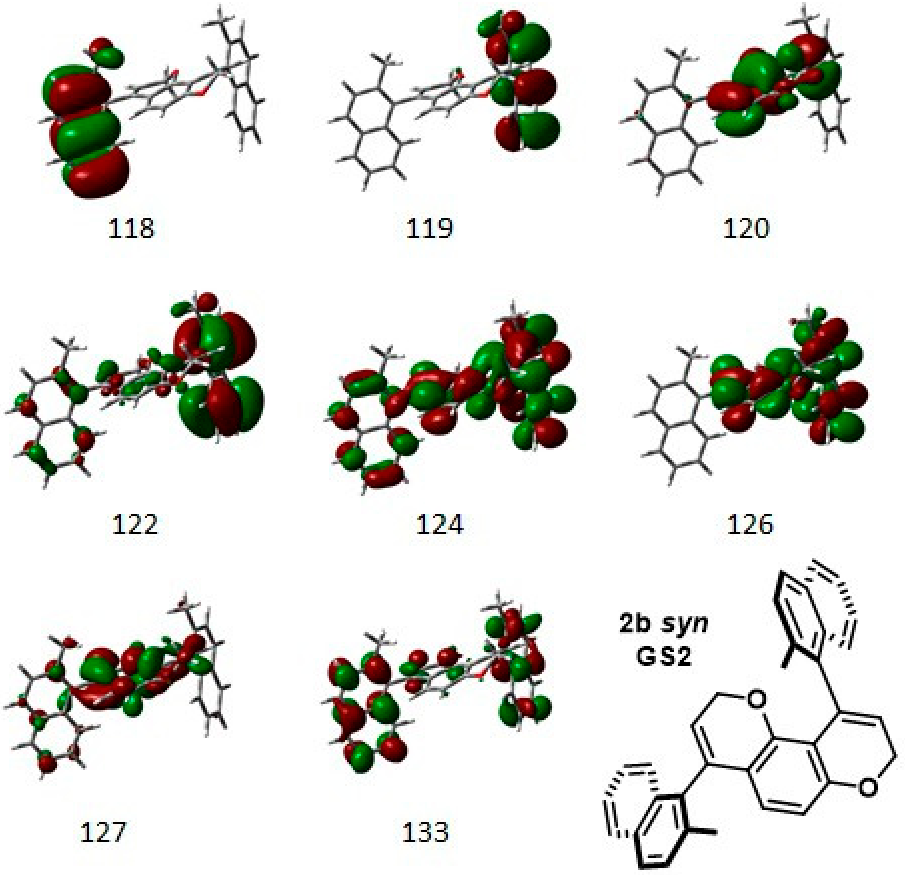

Excited State	Wavelenght (nm)	HOMO	MO	LUMO	%
		122	->	124	30.9
2	282.49	122	->	125	17.3
		122	->	126	10.5
		120	->	127	16.9
14	229.63	122	->	127	19.0
36	206.93	119	->	126	20.2
47	198.77	118	->	129	12.2
61	192.9	120	->	133	13.1

Figure S31. Homo-Lumo orbitals of best calculated GS1 *syn* conformation of compound **2b**.

8. DFT calculations

Table S4. Summarizing tables for experimental and calculated populations:

1a	*anti/syn ratio*	*2a*	*anti/syn ratio*	*1b*	*anti/syn ratio*	*2b*	*anti/syn ratio*
PCM $CHCl_3$	**62:38**	PCM $CHCl_3$	**59:41**	PCM $CHCl_3$	**56:44**	PCM $CHCl_3$	**44:56**
**$C_2D_2Cl_4$**	**60:40**	$C_2D_2Cl_4$	**59:41***	$C_2D_2Cl_4$	**56:44**	$C_2D_2Cl_4$	**51:49**

Table S5. Summarizing tables for experimental and calculated energy barriers (*kJ/mol*):

1a	*Energy barrier*	*2a*	*Energy barrier*	*1b*	*Energy barrier*	*2b*	*Energy barrier*
PCM $CHCl_3$	**TS_{1EXT} 66.5**	PCM $CHCl_3$	**$TS2_{A-EXT-B-UP}$ 66.5 $TS3_{A-UP-B-EXT}$ 70.7**	PCM $CHCl_3$	**TS_{2EXT} 130.5**	PCM $CHCl_3$	**$TS1_{A-INT-B-DO}$ 124.3 $TS4_{A-DO-B-INT}$ 131.4**
D-NMR $C_2D_2Cl_4$	**TS_{1EXT} 65.7**	D-NMR $C_2D_2Cl_4$	**$TS2_{A-EXT-B-UP}$ 15.6 $TS3_{A-UP-B-EXT}$ 74.1**	Kinetic ^{1}HNMR $C_2D_2Cl_4$	**TS_{2EXT} 129.3**	Kinetic ^{1}HNMR $C_2D_2Cl_4$	**$TS_{A-EXT-B-UP}$ 133.9 /**
D-HPLC	**/**	D-HPLC	**/ $TS3_{A-UP-B-EXT}$ 70.7**	Kinetic HPLC cis/trans-decalin	**TS_{2EXT} 129.3**	Kinetic HPLC cis/trans -decalin	**/ /**

Table S6. Comparison for calculated **H**, **G** and **qh-G** (free energy after frequency cut-off at 100 *cm^{-1}*) in *kJ/mol* at 298K for compound **1a/2a**, PCM (chloroform):

1a	*H*	*G*	*qh-G(T)*	*2a*	*H*	*G*	*qh-G(T)*
PCM $CHCl_3$	**TS_{1EXT} 66.5**	**TS_{1EXT} 77.0**	**TS_{1EXT} 72.8**	PCM $CHCl_3$	**$TS2_{A-EXT-B-UP}$ 65.3 $TS3_{A-UP-B-EXT}$ 70.7**	**$TS2_{A-EXT-B-UP}$ 87.1 $TS3_{A-UP-B-EXT}$ 80.7**	**$TS2_{A-EXT-B-UP}$ 72.0 $TS3_{A-UP-B-EXT}$ 77.0**
1a	*anti/syn ratio*	*anti/syn ratio*	*anti/syn ratio*	***2a***	*anti/syn ratio*	*anti/syn ratio*	*anti/syn ratio*
PCM $CHCl_3$	**62:38**	**54:46**	**59:41**	PCM $CHCl_3$	**59:41**	**51:49**	**56:44**

Table S7. Comparison for calculated **H** and **G** in *kJ/mol* at 298K for compound **1a**, with or without **empirical dispersion GD3**, PCM (chloroform):

1a	*H*	*G*	*1a*	*H (GD3)*	*G (GD3)*
PCM $CHCl_3$	**TS_{1EXT} 76.5**	**TS_{1EXT} 77.0**	PCM $CHCl_3$	**TS_{1EXT} 71.5**	**TS_{1EXT} 80.3**

Compound 1a

1a-anti-GS1

Method: opt freq b3lyp/6-311++g(d,p) scrf(solvent=Chloroform)
SCF Done: E(RB3LYP) = -6223.16714758 A.U. after 1 cycles
Lowest frequency = 9.0786

Zero-point correction= (Hartree/Particle)	0.329884
Thermal correction to Energy=	0.353132
Thermal correction to Enthalpy=	0.354077
Thermal correction to Gibbs Free Energy=	0.272168
Sum of electronic and zero-point Energies=	-6222.837263
Sum of electronic and thermal Energies=	-6222.814015
Sum of electronic and thermal Enthalpies=	-6222.813071
Sum of electronic and thermal Free Energies=	-6222.894980

Standard orientation:

Center Number	Atomic Number	Atomic Type	Coordinates (Angstroms) X	Y	Z
1	6	0	-1.169093	-3.023733	-0.274260
2	6	0	-1.186404	-1.613035	-0.301008
3	6	0	-2.437070	-0.947400	-0.698145
4	6	0	-3.560492	-1.676107	-0.763889
5	6	0	-0.000024	-3.725182	0.000005
6	6	0	-0.000011	-0.938320	-0.000002
7	6	0	1.186375	-1.613044	0.301008
8	6	0	1.169051	-3.023742	0.274266
9	6	0	3.560464	-1.676135	0.763879
10	6	0	2.437048	-0.947419	0.698140
11	1	0	-0.000029	-4.807782	0.000007
12	1	0	-4.505682	-1.239106	-1.064938
13	1	0	-0.000006	0.144979	-0.000005
14	1	0	4.505659	-1.239142	1.064925
15	8	0	2.275995	-3.750505	0.601793
16	8	0	-2.276042	-3.750488	-0.601784
17	6	0	3.549875	-3.118784	0.357010
18	1	0	4.272669	-3.704595	0.924596
19	1	0	3.790308	-3.226778	-0.711883
20	6	0	-3.549919	-3.118755	-0.357015
21	1	0	-4.272713	-3.704560	-0.924606
22	1	0	-3.790363	-3.226743	0.711875
23	6	0	2.422761	0.498405	1.071830
24	6	0	2.005510	0.872617	2.358397
25	6	0	2.809909	1.523842	0.201270
26	6	0	1.991588	2.203546	2.762202
27	1	0	1.694153	0.094349	3.045861
28	6	0	2.800777	2.863021	0.587913
29	6	0	2.391138	3.201267	1.874557
30	1	0	1.670528	2.460020	3.764901
31	1	0	3.109527	3.630304	-0.110073
32	1	0	2.384692	4.242092	2.176711
33	6	0	-2.422771	0.498424	-1.071838
34	6	0	-2.809886	1.523866	-0.201270

35	6	0	-2.005539	0.872629	-2.358412
36	6	0	-2.800740	2.863045	-0.587912
37	6	0	-1.991605	2.203559	-2.762217
38	1	0	-1.694209	0.094356	-3.045883
39	6	0	-2.391121	3.201285	-1.874564
40	1	0	-3.109466	3.630333	0.110080
41	1	0	-1.670561	2.460028	-3.764922
42	1	0	-2.384664	4.242110	-2.176717
43	35	0	3.369943	1.124771	-1.599338
44	35	0	-3.369897	1.124800	1.599346

1a-anti-GS2

Method: opt freq b3lyp/6-311++g(d,p) scrf(solvent=Chloroform)
SCF Done: E(RB3LYP) = -6223.16804199 A.U. after 1 cycles
Lowest frequency = 14.9143

Zero-point correction= 0.329951
(Hartree/Particle)
Thermal correction to Energy= 0.353111
Thermal correction to Enthalpy= 0.354055
Thermal correction to Gibbs Free Energy= 0.273376
Sum of electronic and zero-point Energies= -6222.838091
Sum of electronic and thermal Energies= -6222.814931
Sum of electronic and thermal Enthalpies= -6222.813987
Sum of electronic and thermal Free Energies= -6222.894666

Standard orientation:

Center Number	Atomic Number	Atomic Type	Coordinates (Angstroms) X	Y	Z
1	6	0	1.036926	2.675414	0.604372
2	6	0	1.071880	1.265473	0.588527
3	6	0	2.254591	0.600835	1.159110
4	6	0	3.087411	1.322691	1.924111
5	6	0	0.000011	3.377796	-0.000010
6	6	0	-0.000003	0.590178	0.000015
7	6	0	-1.071878	1.265473	-0.588508
8	6	0	-1.036913	2.675413	-0.604377
9	6	0	-3.087418	1.322688	-1.924082
10	6	0	-2.254589	0.600834	-1.159092
11	1	0	0.000015	4.460375	-0.000018
12	1	0	3.978708	0.888062	2.362071
13	1	0	-0.000008	-0.493366	0.000023
14	1	0	-3.978706	0.888049	-2.362053
15	8	0	-2.063069	3.398707	-1.139188
16	8	0	2.063091	3.398701	1.139178
17	6	0	-2.789074	2.764142	-2.211830
18	1	0	-3.701361	3.349486	-2.324663
19	1	0	-2.201880	2.869443	-3.137110
20	6	0	2.789029	2.764137	2.211867
21	1	0	3.701297	3.349496	2.324775
22	1	0	2.201761	2.869414	3.137101
23	6	0	-2.499575	-0.849395	-0.909393
24	6	0	-2.281014	-1.768952	-1.946442
25	6	0	-2.951196	-1.361616	0.314433
26	6	0	-2.499320	-3.132800	-1.771691
27	1	0	-1.927069	-1.393993	-2.899794
28	6	0	-3.175984	-2.722299	0.506412

29	6	0	-2.944811	-3.610661	-0.541694
30	1	0	-2.320285	-3.817322	-2.592507
31	1	0	-3.534600	-3.082187	1.462072
32	1	0	-3.118573	-4.669924	-0.391953
33	6	0	2.499593	-0.849387	0.909396
34	6	0	2.951192	-1.361600	-0.314442
35	6	0	2.281074	-1.768952	1.946448
36	6	0	3.175995	-2.722279	-0.506430
37	6	0	2.499395	-3.132796	1.771688
38	1	0	1.927147	-1.394000	2.899810
39	6	0	2.944860	-3.610647	0.541679
40	1	0	3.534595	-3.082159	-1.462099
41	1	0	2.320391	-3.817322	2.592507
42	1	0	3.118634	-4.669907	0.391930
43	35	0	-3.335373	-0.180520	1.785634
44	35	0	3.335335	-0.180495	-1.785646

1a-anti-GS3

Method: opt freq b3lyp/6-311++g(d,p) scrf(solvent=Chloroform)
SCF Done: E(RB3LYP) = -6223.16765424 A.U. after 1 cycles
Lowest frequency = 12.5752

Zero-point correction= 0.329927
(Hartree/Particle)
Thermal correction to Energy= 0.353124
Thermal correction to Enthalpy= 0.354068
Thermal correction to Gibbs Free Energy= 0.273025
Sum of electronic and zero-point Energies= -6222.837727
Sum of electronic and thermal Energies= -6222.814530
Sum of electronic and thermal Enthalpies= -6222.813586
Sum of electronic and thermal Free Energies= -6222.894629

Standard orientation:

Center Number	Atomic Number	Atomic Type	Coordinates (Angstroms) X	Y	Z
1	6	0	1.400465	2.781356	-0.358276
2	6	0	1.260627	1.390724	-0.547058
3	6	0	2.422864	0.646501	-1.058583
4	6	0	3.437451	1.337689	-1.599572
5	6	0	0.374296	3.541269	0.192422
6	6	0	0.046112	0.800422	-0.189385
7	6	0	-1.014481	1.538797	0.342306
8	6	0	-0.827688	2.925450	0.524852
9	6	0	-3.296081	1.771123	1.114792
10	6	0	-2.285506	0.950727	0.793597
11	1	0	0.503287	4.605195	0.345105
12	1	0	4.322767	0.842901	-1.982380
13	1	0	-0.069912	-0.270123	-0.308613
14	1	0	-4.247917	1.390480	1.467229
15	8	0	-1.792835	3.699679	1.099051
16	8	0	2.572823	3.416387	-0.647453
17	6	0	-3.154314	3.252285	0.932103
18	1	0	-3.499736	3.559477	-0.067033
19	1	0	-3.730377	3.808013	1.671817
20	6	0	3.361444	2.831921	-1.704652
21	1	0	4.345132	3.292316	-1.615293
22	1	0	2.929333	3.139728	-2.669305

23	6	0	-2.411289	-0.530334	0.942051
24	6	0	-2.022452	-1.138433	2.145125
25	6	0	-2.908825	-1.364856	-0.065505
26	6	0	-2.137786	-2.511472	2.338004
27	1	0	-1.627400	-0.510782	2.935682
28	6	0	-3.030010	-2.742163	0.108936
29	6	0	-2.642877	-3.315017	1.317404
30	1	0	-1.834676	-2.951093	3.280961
31	1	0	-3.423339	-3.357015	-0.690145
32	1	0	-2.737830	-4.385831	1.455355
33	6	0	2.452599	-0.843866	-0.995512
34	6	0	2.679273	-1.566248	0.183701
35	6	0	2.254204	-1.583263	-2.171312
36	6	0	2.708039	-2.958284	0.201499
37	6	0	2.278445	-2.975262	-2.171605
38	1	0	2.072658	-1.043746	-3.093689
39	6	0	2.503167	-3.664045	-0.982104
40	1	0	2.895323	-3.483935	1.128839
41	1	0	2.120916	-3.517430	-3.096592
42	1	0	2.524315	-4.747562	-0.968185
43	35	0	-3.457665	-0.630776	-1.760868
44	35	0	3.015707	-0.647774	1.841842

1a-syn-GS1

Method: opt freq b3lyp/6-311++g(d,p) scrf(solvent=Chloroform)
SCF Done: E(RB3LYP) = -6223.16728046 A.U. after 1 cycles
Lowest frequency = 7.8333

Zero-point correction= 0.329911
(Hartree/Particle)
Thermal correction to Energy= 0.353119
Thermal correction to Enthalpy= 0.354063
Thermal correction to Gibbs Free Energy= 0.272494
Sum of electronic and zero-point Energies= -6222.837369
Sum of electronic and thermal Energies= -6222.814162
Sum of electronic and thermal Enthalpies= -6222.813218
Sum of electronic and thermal Free Energies= -6222.894786

Standard orientation:

Center Number	Atomic Number	Atomic Type	Coordinates (Angstroms) X	Y	Z
1	6	0	1.774108	2.668183	0.401340
2	6	0	1.521173	1.298693	0.624990
3	6	0	2.676225	0.414261	0.849166
4	6	0	3.864545	0.971261	1.127000
5	6	0	0.744633	3.550701	0.093400
6	6	0	0.194441	0.862589	0.584842
7	6	0	-0.866349	1.728447	0.304600
8	6	0	-0.562360	3.079311	0.032241
9	6	0	-3.196402	2.185179	-0.159546
10	6	0	-2.283852	1.334446	0.331685
11	1	0	0.958171	4.595115	-0.095336
12	1	0	4.750709	0.368867	1.291205
13	1	0	-0.019293	-0.182163	0.776221
14	1	0	-4.255276	1.953892	-0.148314
15	8	0	-1.548980	3.989483	-0.211060
16	8	0	3.047686	3.157823	0.399346

17	6	0	-2.761087	3.470185	-0.797131
18	1	0	-3.501903	4.258593	-0.666109
19	1	0	-2.594014	3.336676	-1.876953
20	6	0	3.993310	2.461487	1.236722
21	1	0	4.973575	2.805879	0.907814
22	1	0	3.847793	2.795987	2.275528
23	6	0	-2.681767	0.038312	0.957615
24	6	0	-2.744933	-0.056470	2.356675
25	6	0	-3.005228	-1.108956	0.223577
26	6	0	-3.128690	-1.232547	2.992098
27	1	0	-2.492664	0.819255	2.943955
28	6	0	-3.389839	-2.296932	0.843789
29	6	0	-3.452772	-2.355975	2.232942
30	1	0	-3.176057	-1.271200	4.074063
31	1	0	-3.635461	-3.164613	0.245418
32	1	0	-3.753448	-3.278805	2.715303
33	6	0	2.513540	-1.067631	0.791487
34	6	0	2.367319	-1.785321	-0.403304
35	6	0	2.512250	-1.804013	1.985694
36	6	0	2.223620	-3.170260	-0.417869
37	6	0	2.368774	-3.188873	1.989508
38	1	0	2.620808	-1.267356	2.921181
39	6	0	2.220966	-3.872795	0.785254
40	1	0	2.121303	-3.693717	-1.359593
41	1	0	2.371219	-3.729038	2.928977
42	1	0	2.107681	-4.950604	0.773865
43	35	0	-2.918304	-1.103973	-1.701274
44	35	0	2.411945	-0.876453	-2.099131

1a-syn-GS2
Method: opt freq b3lyp/6-311++g(d,p) scrf(solvent=Chloroform)
SCF Done: E(RB3LYP) = -6223.16717319 A.U. after 1 cycles
Lowest frequency = 15.9570

Zero-point correction= 0.329950
(Hartree/Particle)
Thermal correction to Energy= 0.353082
Thermal correction to Enthalpy= 0.354026
Thermal correction to Gibbs Free Energy= 0.274115
Sum of electronic and zero-point Energies= -6222.837223
Sum of electronic and thermal Energies= -6222.814092
Sum of electronic and thermal Enthalpies= -6222.813147
Sum of electronic and thermal Free Energies= -6222.893058

Standard orientation:

Center Number	Atomic Number	Atomic Type	Coordinates (Angstroms) X	Y	Z
1	6	0	-1.202702	2.877117	-0.141439
2	6	0	-1.225297	1.534812	-0.573754
3	6	0	-2.537645	0.888638	-0.739050
4	6	0	-3.633936	1.660982	-0.760466
5	6	0	-0.002742	3.540023	0.092154
6	6	0	-0.001499	0.892014	-0.778598
7	6	0	1.221698	1.536149	-0.574359
8	6	0	1.197839	2.878372	-0.141817
9	6	0	3.630160	1.664958	-0.761933
10	6	0	2.534683	0.891488	-0.740547
11	1	0	-0.003216	4.567916	0.431787

12	1	0	-4.625618	1.237635	-0.872676
13	1	0	-0.001019	-0.145396	-1.091432
14	1	0	4.622212	1.242682	-0.874915
15	8	0	2.353774	3.554401	0.120487
16	8	0	-2.359298	3.551971	0.121022
17	6	0	3.507076	3.155329	-0.648103
18	1	0	3.440205	3.623953	-1.642197
19	1	0	4.360920	3.590721	-0.129268
20	6	0	-3.512205	3.151534	-0.647454
21	1	0	-4.366464	3.586446	-0.128907
22	1	0	-3.445642	3.619700	-1.641784
23	6	0	2.631853	-0.588265	-0.908830
24	6	0	2.836749	-1.121612	-2.190024
25	6	0	2.537738	-1.494601	0.155390
26	6	0	2.940749	-2.493901	-2.401052
27	1	0	2.910419	-0.436928	-3.027207
28	6	0	2.640989	-2.870015	-0.037481
29	6	0	2.840036	-3.369653	-1.322578
30	1	0	3.099547	-2.875551	-3.402834
31	1	0	2.572681	-3.541654	0.808472
32	1	0	2.919611	-4.440068	-1.472827
33	6	0	-2.633405	-0.591306	-0.906202
34	6	0	-2.535092	-1.496890	0.158303
35	6	0	-2.841342	-1.125706	-2.186473
36	6	0	-2.637082	-2.872549	-0.033449
37	6	0	-2.944168	-2.498255	-2.396367
38	1	0	-2.918245	-0.441624	-3.023854
39	6	0	-2.839173	-3.373226	-1.317669
40	1	0	-2.565514	-3.543580	0.812716
41	1	0	-3.105354	-2.880726	-3.397455
42	1	0	-2.917770	-4.443835	-1.467051
43	35	0	2.303878	-0.859885	1.956156
44	35	0	-2.297371	-0.860780	1.958077

1a-syn-GS3
Method: opt freq b3lyp/6-311++g(d,p) scrf(solvent=Chloroform) iop(1/8=1, 1/9
SCF Done: E(RB3LYP) = -6223.16710227 A.U. after 2 cycles
Lowest frequency = 8.5585

Zero-point correction= 0.329843
(Hartree/Particle)
Thermal correction to Energy= 0.353096
Thermal correction to Enthalpy= 0.354040
Thermal correction to Gibbs Free Energy= 0.272021
Sum of electronic and zero-point Energies= -6222.837259
Sum of electronic and thermal Energies= -6222.814006
Sum of electronic and thermal Enthalpies= -6222.813062
Sum of electronic and thermal Free Energies= -6222.895081

Standard orientation:

Center Number	Atomic Number	Atomic Type	Coordinates (Angstroms) X	Y	Z
1	6	0	1.200266	3.035759	0.309381
2	6	0	1.223895	1.626116	0.369683
3	6	0	2.534435	0.964294	0.468085
4	6	0	3.639079	1.687193	0.234055
5	6	0	-0.000567	3.737166	0.298154

6	6	0	-0.000176	0.952394	0.403053
7	6	0	-1.224435	1.625779	0.369951
8	6	0	-1.201200	3.035431	0.309709
9	6	0	-3.639673	1.686206	0.234896
10	6	0	-2.534771	0.963573	0.468513
11	1	0	-0.000720	4.819252	0.264774
12	1	0	4.629652	1.253368	0.308670
13	1	0	-0.000020	-0.128431	0.476864
14	1	0	-4.630099	1.252055	0.309584
15	8	0	-2.354634	3.762936	0.335514
16	8	0	2.353511	3.763563	0.334794
17	6	0	-3.527597	3.116032	-0.201192
18	1	0	-4.368932	3.713448	0.149459
19	1	0	-3.492364	3.190701	-1.298881
20	6	0	3.526433	3.116893	-0.202304
21	1	0	3.490667	3.191329	-1.299990
22	1	0	4.367749	3.714623	0.147851
23	6	0	-2.616058	-0.469551	0.881349
24	6	0	-2.596495	-0.795334	2.245989
25	6	0	-2.717820	-1.528249	-0.028611
26	6	0	-2.688599	-2.113124	2.682132
27	1	0	-2.513055	0.010102	2.966852
28	6	0	-2.808603	-2.854628	0.390019
29	6	0	-2.794812	-3.145247	1.751398
30	1	0	-2.678388	-2.332528	3.743324
31	1	0	-2.889606	-3.648765	-0.340784
32	1	0	-2.866999	-4.176097	2.078195
33	6	0	2.616240	-0.468692	0.881303
34	6	0	2.718349	-1.527622	-0.028331
35	6	0	2.596874	-0.794069	2.246051
36	6	0	2.809686	-2.853842	0.390696
37	6	0	2.689517	-2.111687	2.682589
38	1	0	2.513164	0.011557	2.966669
39	6	0	2.796091	-3.144053	1.752160
40	1	0	2.890954	-3.648166	-0.339873
41	1	0	2.679451	-2.330778	3.743847
42	1	0	2.868707	-4.174774	2.079269
43	35	0	-2.734286	-1.191073	-1.925382
44	35	0	2.734528	-1.191022	-1.925208

1a-TS1-ext
Method: opt(ts,calcfc,noeigen) freq b3lyp/6-311++g(d,p) scrf(solvent=Chlorof
SCF Done: E(RB3LYP) = -6223.14216740 A.U. after 1 cycles
Lowest frequency = -39.4369

Zero-point correction= 0.330516
(Hartree/Particle)
Thermal correction to Energy= 0.352591
Thermal correction to Enthalpy= 0.353535
Thermal correction to Gibbs Free Energy= 0.276413
Sum of electronic and zero-point Energies= -6222.811651
Sum of electronic and thermal Energies= -6222.789576
Sum of electronic and thermal Enthalpies= -6222.788632
Sum of electronic and thermal Free Energies= -6222.865754

Standard orientation:

Center Number	Atomic Number	Atomic Type	Coordinates (Angstroms) X	Y	Z

1	6	0	-0.718656	2.691531	-0.327435
2	6	0	-0.727685	1.296371	-0.089889
3	6	0	-2.046323	0.694713	0.308122
4	6	0	-2.837422	1.532474	1.011950
5	6	0	0.396476	3.356563	-0.827545
6	6	0	0.520536	0.661222	-0.174713
7	6	0	1.677824	1.306368	-0.622442
8	6	0	1.586923	2.662845	-0.989218
9	6	0	4.056110	1.379014	-1.068253
10	6	0	2.976953	0.633873	-0.789471
11	1	0	0.346183	4.418629	-1.030584
12	1	0	-3.786730	1.233244	1.418504
13	1	0	0.631248	-0.362484	0.147253
14	1	0	5.032344	0.930651	-1.213987
15	8	0	2.649780	3.328902	-1.526104
16	8	0	-1.819506	3.444404	-0.032199
17	6	0	3.958175	2.874037	-1.123087
18	1	0	4.646112	3.296132	-1.855723
19	1	0	4.193995	3.317621	-0.143553
20	6	0	-2.458597	2.969520	1.171438
21	1	0	-1.779396	3.141600	2.019824
22	1	0	-3.340672	3.594817	1.303644
23	6	0	3.066067	-0.855259	-0.708646
24	6	0	2.885131	-1.621490	-1.870157
25	6	0	3.331124	-1.546299	0.479687
26	6	0	2.975213	-3.010016	-1.848421
27	1	0	2.675260	-1.105787	-2.800338
28	6	0	3.422659	-2.936342	0.520812
29	6	0	3.244040	-3.668661	-0.649837
30	1	0	2.838674	-3.573951	-2.763696
31	1	0	3.633803	-3.437955	1.456271
32	1	0	3.317564	-4.749537	-0.619728
33	6	0	-2.495723	-0.673537	-0.119838
34	6	0	-3.824968	-1.179158	-0.091525
35	6	0	-1.559103	-1.581907	-0.661851
36	6	0	-4.136899	-2.487108	-0.463802
37	6	0	-1.859480	-2.880495	-1.050228
38	1	0	-0.547144	-1.255590	-0.809677
39	6	0	-3.157688	-3.355568	-0.928143
40	1	0	-5.165575	-2.816909	-0.407569
41	1	0	-1.072786	-3.509505	-1.449707
42	1	0	-3.420223	-4.367477	-1.212624
43	35	0	-5.434035	-0.171020	0.324577
44	35	0	3.591378	-0.586582	2.129796

1a-TS2-int

Method: opt(ts,calcfc,noeigen) freq b3lyp/6-311++g(d,p) scrf(solvent=Chlorof

SCF Done: E(RB3LYP) = -6223.12825759 A.U. after 1 cycles

Lowest frequency = -35.7809

Zero-point correction= 0.330310
(Hartree/Particle)

Thermal correction to Energy= 0.352312

Thermal correction to Enthalpy= 0.353256

Thermal correction to Gibbs Free Energy= 0.277027

Sum of electronic and zero-point Energies= -6222.797947

Sum of electronic and thermal Energies= -6222.775946

Sum of electronic and thermal Enthalpies= -6222.775002

Sum of electronic and thermal Free Energies= -6222.851231

Standard orientation:

Center Number	Atomic Number	Atomic Type	Coordinates (Angstroms) X	Y	Z
1	6	0	-1.030044	2.958870	-0.358474
2	6	0	-1.144740	1.593487	-0.005806
3	6	0	-2.463581	1.167658	0.577767
4	6	0	-3.023621	2.131274	1.347766
5	6	0	0.096076	3.473335	-0.991136
6	6	0	0.037104	0.848259	-0.083387
7	6	0	1.217671	1.347783	-0.645185
8	6	0	1.210501	2.662694	-1.151165
9	6	0	3.573349	1.197293	-1.194083
10	6	0	2.460313	0.568429	-0.787948
11	1	0	0.127070	4.515651	-1.281254
12	1	0	-3.923564	1.976277	1.924132
13	1	0	0.057586	-0.149858	0.319297
14	1	0	4.508727	0.664101	-1.321413
15	8	0	2.286522	3.182214	-1.812151
16	8	0	-2.019901	3.836772	-0.006884
17	6	0	3.579206	2.678595	-1.420650
18	1	0	4.253966	2.959547	-2.229613
19	1	0	3.901061	3.212121	-0.512830
20	6	0	-2.484315	3.525187	1.324701
21	1	0	-1.656294	3.683416	2.031338
22	1	0	-3.269048	4.248439	1.543978
23	6	0	2.474041	-0.904994	-0.542117
24	6	0	2.352018	-1.785411	-1.627900
25	6	0	2.632757	-1.473772	0.726965
26	6	0	2.388279	-3.165957	-1.455259
27	1	0	2.227746	-1.365313	-2.619362
28	6	0	2.667319	-2.853353	0.919633
29	6	0	2.543401	-3.700960	-0.177943
30	1	0	2.295368	-3.819719	-2.314523
31	1	0	2.795513	-3.258204	1.915118
32	1	0	2.571265	-4.774185	-0.029759
33	6	0	-3.283600	-0.081055	0.303089
34	6	0	-2.937959	-1.347362	-0.229240
35	6	0	-4.670745	0.034221	0.599714
36	6	0	-3.851447	-2.407877	-0.298659
37	6	0	-5.584432	-1.002339	0.527106
38	1	0	-5.060351	1.001392	0.872766
39	6	0	-5.168776	-2.258557	0.099357
40	1	0	-3.513179	-3.357209	-0.691362
41	1	0	-6.620945	-0.815894	0.782494
42	1	0	-5.856746	-3.092909	0.033841
43	35	0	-1.258019	-1.848018	-1.040017
44	35	0	2.843625	-0.352332	2.279633

Compound 2a:
2a-anti-GS1
Method: opt freq b3lyp/6-311++g(d,p) scrf(solvent=Chloroform)

SCF Done: E(RB3LYP) = -6223.16229827 A.U. after 1 cycles
Lowest frequency = 13.7385

Zero-point correction= 0.330013
(Hartree/Particle)
Thermal correction to Energy= 0.353144
Thermal correction to Enthalpy= 0.354089
Thermal correction to Gibbs Free Energy= 0.273471
Sum of electronic and zero-point Energies= -6222.832285
Sum of electronic and thermal Energies= -6222.809154
Sum of electronic and thermal Enthalpies= -6222.808210
Sum of electronic and thermal Free Energies= -6222.888827

Standard orientation:

Center Number	Atomic Number	Atomic Type	Coordinates (Angstroms) X	Y	Z
1	6	0	0.856689	2.862704	0.630366
2	6	0	-0.483472	2.526379	0.794031
3	6	0	-0.997551	1.280124	0.367558
4	6	0	-0.119222	0.426098	-0.322928
5	6	0	1.245434	0.743254	-0.491191
6	6	0	1.710720	1.966053	0.001580
7	8	0	-0.604308	-0.764888	-0.773497
8	6	0	0.044673	-1.317311	-1.935650
9	6	0	1.537930	-1.254326	-1.827752
10	6	0	2.111328	-0.254882	-1.141910
11	8	0	-1.286162	3.425057	1.435671
12	6	0	-2.676873	3.383239	1.057883
13	6	0	-3.197450	1.979899	1.079785
14	6	0	-2.396853	0.964705	0.720468
15	6	0	-2.884551	-0.443577	0.830220
16	6	0	-3.823583	-1.016670	-0.033496
17	6	0	-4.291663	-2.317275	0.142543
18	6	0	-3.815599	-3.079892	1.205510
19	6	0	-2.874781	-2.538516	2.080113
20	6	0	-2.419888	-1.238852	1.888353
21	6	0	3.595721	-0.129173	-1.058873
22	6	0	4.311796	0.287663	-2.191324
23	6	0	5.697761	0.418992	-2.172325
24	6	0	6.404319	0.133364	-1.006558
25	6	0	5.722772	-0.288625	0.132981
26	6	0	4.336393	-0.413571	0.096418
27	35	0	-4.488614	-0.042971	-1.560070
28	35	0	3.458606	-1.046567	1.688248
29	1	0	1.208504	3.821230	0.990319
30	1	0	2.756435	2.222532	-0.119369
31	1	0	-0.314180	-2.344153	-1.999288
32	1	0	-0.314632	-0.775721	-2.824035
33	1	0	2.125176	-2.021142	-2.319768
34	1	0	-3.191023	4.018521	1.777899
35	1	0	-2.781417	3.837838	0.060963
36	1	0	-4.220169	1.809026	1.394876
37	1	0	-5.017070	-2.729294	-0.546995
38	1	0	-4.179804	-4.091328	1.343623
39	1	0	-2.498591	-3.124996	2.910224

40	1	0	-1.689469	-0.815920	2.568903
41	1	0	3.757810	0.516204	-3.094488
42	1	0	6.221201	0.744223	-3.063728
43	1	0	7.483218	0.231978	-0.977567
44	1	0	6.263739	-0.525320	1.040016

2a-anti-GS2

Method: opt freq b3lyp/6-311++g(d,p) scrf(solvent=Chloroform)
SCF Done: E(RB3LYP) = -6223.16326195 A.U. after 1 cycles
Lowest frequency = 17.3605

Zero-point correction= 0.330103
(Hartree/Particle)
Thermal correction to Energy= 0.353174
Thermal correction to Enthalpy= 0.354118
Thermal correction to Gibbs Free Energy= 0.274094
Sum of electronic and zero-point Energies= -6222.833159
Sum of electronic and thermal Energies= -6222.810088
Sum of electronic and thermal Enthalpies= -6222.809144
Sum of electronic and thermal Free Energies= -6222.889168

Standard orientation:

Center Number	Atomic Number	Atomic Type	Coordinates (Angstroms) X	Y	Z
1	6	0	0.580965	2.062561	-1.753930
2	6	0	-0.653837	2.172154	-1.122514
3	6	0	-1.116524	1.191343	-0.215179
4	6	0	-0.237810	0.141329	0.096621
5	6	0	1.040238	0.039312	-0.490426
6	6	0	1.416551	1.000298	-1.433461
7	8	0	-0.622630	-0.752478	1.052271
8	6	0	-0.083927	-2.084196	0.933531
9	6	0	1.387500	-2.069413	0.648767
10	6	0	1.921505	-1.053110	-0.044129
11	8	0	-1.445302	3.231208	-1.461912
12	6	0	-2.335483	3.680515	-0.421855
13	6	0	-3.087738	2.531389	0.175290
14	6	0	-2.500539	1.328275	0.284597
15	6	0	-3.279674	0.191727	0.860420
16	6	0	-3.729790	-0.902408	0.111397
17	6	0	-4.492027	-1.921603	0.675697
18	6	0	-4.828232	-1.860641	2.026374
19	6	0	-4.408367	-0.777657	2.795285
20	6	0	-3.647262	0.231568	2.213415
21	6	0	3.373146	-1.034092	-0.388495
22	6	0	3.853213	-1.897688	-1.384690
23	6	0	5.198370	-1.923510	-1.743135
24	6	0	6.101170	-1.075805	-1.105803
25	6	0	5.656443	-0.210558	-0.108384
26	6	0	4.307489	-0.197403	0.236704
27	35	0	-3.347728	-1.024818	-1.773640
28	35	0	3.762408	0.986714	1.653345
29	1	0	0.879868	2.821866	-2.465407
30	1	0	2.392513	0.932294	-1.899122
31	1	0	-0.306788	-2.566142	1.885138
32	1	0	-0.637790	-2.616059	0.145260

33	1	0	1.990606	-2.895822	1.007158
34	1	0	-1.744108	4.215889	0.336429
35	1	0	-3.003075	4.395741	-0.900691
36	1	0	-4.113297	2.688142	0.489034
37	1	0	-4.827758	-2.748542	0.063251
38	1	0	-5.420977	-2.654079	2.466607
39	1	0	-4.669941	-0.718234	3.845378
40	1	0	-3.308505	1.068218	2.813115
41	1	0	3.146239	-2.550397	-1.883699
42	1	0	5.537484	-2.601574	-2.517477
43	1	0	7.150797	-1.085059	-1.375642
44	1	0	6.352092	0.443230	0.401536

2a-syn-GS1

Method: opt freq b3lyp/6-311++g(d,p) scrf(solvent=Chloroform)
SCF Done: E(RB3LYP) = -6223.16232288 A.U. after 1 cycles
Lowest frequency = 14.8887

Zero-point correction= 0.330032
(Hartree/Particle)
Thermal correction to Energy= 0.353121
Thermal correction to Enthalpy= 0.354065
Thermal correction to Gibbs Free Energy= 0.274093
Sum of electronic and zero-point Energies= -6222.832290
Sum of electronic and thermal Energies= -6222.809202
Sum of electronic and thermal Enthalpies= -6222.808258
Sum of electronic and thermal Free Energies= -6222.888230

Standard orientation:

Center Number	Atomic Number	Atomic Type	Coordinates (Angstroms) X	Y	Z
1	6	0	-0.530463	2.760394	-1.075000
2	6	0	0.824497	2.527932	-0.863244
3	6	0	1.281112	1.528444	0.025894
4	6	0	0.306855	0.835355	0.765075
5	6	0	-1.072997	1.051527	0.562865
6	6	0	-1.466565	2.014815	-0.370319
7	8	0	0.726160	-0.118623	1.643690
8	6	0	-0.131562	-0.368270	2.773635
9	6	0	-1.572033	-0.475227	2.375252
10	6	0	-2.022326	0.216887	1.318952
11	8	0	1.712143	3.270061	-1.589039
12	6	0	2.977682	3.510331	-0.945429
13	6	0	3.551285	2.243229	-0.391036
14	6	0	2.738368	1.285498	0.084797
15	6	0	3.338053	0.022407	0.608594
16	6	0	3.217766	-1.218213	-0.029646
17	6	0	3.826525	-2.366560	0.468396
18	6	0	4.585890	-2.291945	1.634077
19	6	0	4.738155	-1.069054	2.283526
20	6	0	4.121765	0.068514	1.770921
21	6	0	-3.462259	0.176010	0.929140
22	6	0	-4.403345	0.860273	1.713266
23	6	0	-5.759854	0.852605	1.399586
24	6	0	-6.207814	0.152724	0.281789
25	6	0	-5.298646	-0.541452	-0.513538
26	6	0	-3.945266	-0.522459	-0.185780

27	35	0	2.230686	-1.381426	-1.674798
28	35	0	-2.744857	-1.537533	-1.294914
29	1	0	-0.831057	3.524477	-1.780636
30	1	0	-2.522546	2.188920	-0.539077
31	1	0	0.237446	-1.296091	3.210094
32	1	0	0.019231	0.436752	3.509683
33	1	0	-2.224801	-1.109611	2.963841
34	1	0	3.617379	3.939767	-1.715595
35	1	0	2.832472	4.267142	-0.159187
36	1	0	4.627083	2.111580	-0.405120
37	1	0	3.715170	-3.307550	-0.054757
38	1	0	5.058867	-3.186416	2.022611
39	1	0	5.332992	-0.999676	3.186921
40	1	0	4.228983	1.018698	2.281166
41	1	0	-4.049997	1.409465	2.578395
42	1	0	-6.461532	1.391736	2.025192
43	1	0	-7.260829	0.138832	0.025651
44	1	0	-5.638233	-1.098511	-1.377129

2a-syn-GS2
Method: opt freq b3lyp/6-311++g(d,p) scrf(solvent=Chloroform)
SCF Done: E(RB3LYP) = -6223.16271061 A.U. after 1 cycles
Lowest frequency = 9.6202

Zero-point correction= 0.330055
(Hartree/Particle)
Thermal correction to Energy= 0.353176
Thermal correction to Enthalpy= 0.354120
Thermal correction to Gibbs Free Energy= 0.273298
Sum of electronic and zero-point Energies= -6222.832656
Sum of electronic and thermal Energies= -6222.809534
Sum of electronic and thermal Enthalpies= -6222.808590
Sum of electronic and thermal Free Energies= -6222.889412

Standard orientation:

Center Number	Atomic Number	Atomic Type	Coordinates (Angstroms) X	Y	Z
1	6	0	-0.427017	2.566916	-1.276774
2	6	0	0.910693	2.452368	-0.912159
3	6	0	1.354888	1.456480	-0.012328
4	6	0	0.375727	0.635980	0.571080
5	6	0	-0.992061	0.761840	0.249074
6	6	0	-1.365233	1.724309	-0.694414
7	8	0	0.774306	-0.244736	1.532843
8	6	0	-0.028977	-1.431511	1.690807
9	6	0	-1.494210	-1.119203	1.691114
10	6	0	-1.954511	-0.087060	0.970769
11	8	0	1.802322	3.297131	-1.507271
12	6	0	2.962380	3.613533	-0.713465
13	6	0	3.585439	2.372711	-0.152181
14	6	0	2.812678	1.330662	0.195825
15	6	0	3.459796	0.094218	0.727917
16	6	0	3.521970	-1.115723	0.025667
17	6	0	4.173148	-2.234727	0.537700
18	6	0	4.790402	-2.160187	1.784456
19	6	0	4.759062	-0.966217	2.501280
20	6	0	4.103127	0.141657	1.973335
21	6	0	-3.410393	0.245671	0.931111

22	6	0	-3.922937	1.199673	1.823432
23	6	0	-5.273436	1.533273	1.838017
24	6	0	-6.148980	0.918008	0.944782
25	6	0	-5.669547	-0.026774	0.041730
26	6	0	-4.313466	-0.349542	0.042549
27	35	0	2.736883	-1.274793	-1.726573
28	35	0	-3.709761	-1.654292	-1.240277
29	1	0	-0.716733	3.326628	-1.991621
30	1	0	-2.411354	1.835013	-0.953349
31	1	0	0.226155	-2.136057	0.884947
32	1	0	0.293922	-1.868706	2.635353
33	1	0	-2.159026	-1.768388	2.249274
34	1	0	3.640115	4.136890	-1.386737
35	1	0	2.660846	4.311788	0.082160
36	1	0	4.664539	2.329012	-0.060580
37	1	0	4.205827	-3.151356	-0.037055
38	1	0	5.296997	-3.031245	2.183621
39	1	0	5.241620	-0.896456	3.469244
40	1	0	4.066605	1.067587	2.535288
41	1	0	-3.238970	1.677047	2.515974
42	1	0	-5.640172	2.269174	2.543937
43	1	0	-7.203334	1.168947	0.945808
44	1	0	-6.340576	-0.509195	-0.657041

2a-TS4-A-fix-DOWN-B-int
Method: opt(ts,calcfc,noeigen) freq b3lyp/6-311++g(d,p) scrf(solvent=Chlorof
SCF Done: E(RB3LYP) = -6223.11744317 A.U. after 1 cycles
Lowest frequency = -24.5883

Zero-point correction= 0.329931
(Hartree/Particle)
Thermal correction to Energy= 0.351879
Thermal correction to Enthalpy= 0.352824
Thermal correction to Gibbs Free Energy= 0.277053
Sum of electronic and zero-point Energies= -6222.787512
Sum of electronic and thermal Energies= -6222.765564
Sum of electronic and thermal Enthalpies= -6222.764620
Sum of electronic and thermal Free Energies= -6222.840390

Standard orientation:

Center Number	Atomic Number	Atomic Type	Coordinates (Angstroms) X	Y	Z
1	6	0	0.400790	2.832566	-1.276656
2	6	0	-0.902976	2.618069	-0.841366
3	6	0	-1.244842	1.560894	0.038023
4	6	0	-0.174550	1.044224	0.787011
5	6	0	1.164077	1.219816	0.361656
6	6	0	1.421509	2.077121	-0.711567
7	8	0	-0.454603	0.392280	1.939787
8	6	0	0.598364	0.303663	2.922118
9	6	0	1.931010	0.005293	2.307389
10	6	0	2.209314	0.474482	1.082396
11	8	0	-1.879982	3.511291	-1.190475
12	6	0	-2.761061	3.745005	-0.054406
13	6	0	-3.436334	2.452268	0.282488
14	6	0	-2.716504	1.311368	0.192790
15	6	0	-3.477653	0.045866	-0.151942

16	6	0	-3.233900	-1.323403	0.105826
17	6	0	-4.143134	-2.322020	-0.262139
18	6	0	-5.313421	-2.022732	-0.942637
19	6	0	-5.553762	-0.700392	-1.295656
20	6	0	-4.649541	0.281553	-0.922818
21	6	0	3.549544	0.270392	0.460622
22	6	0	4.652734	0.972143	0.970364
23	6	0	5.927637	0.821355	0.431497
24	6	0	6.127654	-0.044280	-0.641066
25	6	0	5.053633	-0.760539	-1.165198
26	6	0	3.784878	-0.597089	-0.615111
27	35	0	-1.652250	-2.061081	0.910845
28	35	0	2.353930	-1.655008	-1.346434
29	1	0	0.602728	3.618588	-1.993174
30	1	0	2.441980	2.213337	-1.049516
31	1	0	0.620973	1.252854	3.478732
32	1	0	0.280777	-0.481533	3.608363
33	1	0	2.647399	-0.575603	2.876612
34	1	0	-2.160606	4.165765	0.762547
35	1	0	-3.479481	4.492330	-0.387285
36	1	0	-4.513820	2.449464	0.384375
37	1	0	-3.911789	-3.349633	-0.016198
38	1	0	-5.998690	-2.815365	-1.218793
39	1	0	-6.425254	-0.429081	-1.879815
40	1	0	-4.832961	1.286572	-1.270995
41	1	0	4.491406	1.650939	1.799843
42	1	0	6.758672	1.378999	0.847204
43	1	0	7.114501	-0.170614	-1.070956
44	1	0	5.201260	-1.446193	-1.989528

2a-TS2-B-fix-DOWN-A-ext

Method: opt(ts,calcfc,noeigen) freq b3lyp/6-311++g(d,p) scrf(solvent=Chlorof
SCF Done: E(RB3LYP) = -6223.13721221 A.U. after 1 cycles
Lowest frequency = -39.2377

Zero-point correction= 0.330575
(Hartree/Particle)
Thermal correction to Energy= 0.352562
Thermal correction to Enthalpy= 0.353506
Thermal correction to Gibbs Free Energy= 0.276839
Sum of electronic and zero-point Energies= -6222.806637
Sum of electronic and thermal Energies= -6222.784650
Sum of electronic and thermal Enthalpies= -6222.783706
Sum of electronic and thermal Free Energies= -6222.860373

Standard orientation:

Center Number	Atomic Number	Atomic Type	Coordinates (Angstroms) X	Y	Z
1	6	0	0.092165	3.532975	-0.674255
2	6	0	1.308909	2.974945	-0.302168
3	6	0	1.485065	1.573943	-0.275914
4	6	0	0.344508	0.780700	-0.488895
5	6	0	-0.951025	1.323490	-0.668093
6	6	0	-1.013542	2.712017	-0.863164
7	8	0	0.506667	-0.572455	-0.545681
8	6	0	-0.327930	-1.156436	-1.565949

9	6	0	-1.760135	-0.844925	-1.276817
10	6	0	-2.089087	0.343905	-0.729365
11	8	0	2.358503	3.817977	-0.088712
12	6	0	3.389069	3.294387	0.774060
13	6	0	3.792020	1.917707	0.345264
14	6	0	2.868338	1.072432	-0.137555
15	6	0	3.290339	-0.271591	-0.639464
16	6	0	3.581305	-1.357089	0.192838
17	6	0	4.029575	-2.575203	-0.313552
18	6	0	4.197086	-2.727684	-1.687421
19	6	0	3.916115	-1.663244	-2.542393
20	6	0	3.469516	-0.454602	-2.018331
21	6	0	-3.446312	0.619626	-0.147408
22	6	0	-3.809025	1.940493	0.199173
23	6	0	-5.049646	2.306105	0.703904
24	6	0	-6.023892	1.339462	0.910747
25	6	0	-5.702798	0.013187	0.652633
26	6	0	-4.446660	-0.343095	0.160885
27	35	0	3.339119	-1.227480	2.099898
28	35	0	-4.243755	-2.274918	0.095341
29	1	0	0.017240	4.605791	-0.800455
30	1	0	-1.928436	3.180508	-1.193006
31	1	0	0.004248	-0.787548	-2.547926
32	1	0	-0.142188	-2.228722	-1.519927
33	1	0	-2.476558	-1.627887	-1.450369
34	1	0	4.214262	4.001438	0.702527
35	1	0	3.014223	3.303280	1.808454
36	1	0	4.832927	1.628429	0.430040
37	1	0	4.242922	-3.395302	0.359776
38	1	0	4.547430	-3.674502	-2.081815
39	1	0	4.049103	-1.771455	-3.612605
40	1	0	3.256026	0.376498	-2.681274
41	1	0	-3.080851	2.722241	0.090756
42	1	0	-5.242194	3.346872	0.936383
43	1	0	-7.004541	1.595918	1.293291
44	1	0	-6.426480	-0.764829	0.855355

\- 2a-TS3-A-fix-UP-B-ext

Method: opt(ts,calcfc,noeigen) freq b3lyp/6-311++g(d,p) scrf(solvent=Chlorof
SCF Done: E(RB3LYP) = -6223.13541046 A.U. after 1 cycles
Lowest frequency = -39.2879

Zero-point correction= 0.330318
(Hartree/Particle)
Thermal correction to Energy= 0.352304
Thermal correction to Enthalpy= 0.353248
Thermal correction to Gibbs Free Energy= 0.276776
Sum of electronic and zero-point Energies= -6222.805092
Sum of electronic and thermal Energies= -6222.783107
Sum of electronic and thermal Enthalpies= -6222.782163
Sum of electronic and thermal Free Energies= -6222.858634

Standard orientation:

Center Number	Atomic Number	Atomic Type	Coordinates (Angstroms) X	Y	Z
1	6	0	0.713257	-1.704051	1.731598
2	6	0	-0.500517	-1.641829	1.057638

3	6	0	-0.857845	-0.542559	0.237070
4	6	0	0.201770	0.305749	-0.134330
5	6	0	1.472993	0.223541	0.474939
6	6	0	1.682117	-0.748740	1.457019
7	8	0	0.019085	1.135028	-1.209318
8	6	0	0.820671	2.331668	-1.280753
9	6	0	2.237417	2.103837	-0.857459
10	6	0	2.543839	1.109295	-0.014073
11	8	0	-1.355988	-2.701944	1.157935
12	6	0	-1.993442	-2.959537	-0.116898
13	6	0	-2.777076	-1.750400	-0.516915
14	6	0	-2.289334	-0.525421	-0.232003
15	6	0	-3.159084	0.694999	-0.177360
16	6	0	-4.576801	0.710174	-0.077155
17	6	0	-5.309885	1.896727	-0.036748
18	6	0	-4.674514	3.131780	-0.044792
19	6	0	-3.286391	3.162544	-0.062940
20	6	0	-2.569934	1.975313	-0.122084
21	6	0	3.948986	0.909591	0.450036
22	6	0	4.474278	1.761288	1.433139
23	6	0	5.780286	1.624085	1.895788
24	6	0	6.596053	0.620366	1.378916
25	6	0	6.104625	-0.238679	0.398357
26	6	0	4.795393	-0.087798	-0.051688
27	35	0	-5.727603	-0.839081	0.152606
28	35	0	4.183974	-1.281592	-1.432289
29	1	0	0.908270	-2.534622	2.398110
30	1	0	2.646391	-0.810984	1.946345
31	1	0	0.757262	2.649211	-2.321796
32	1	0	0.349616	3.115178	-0.665281
33	1	0	2.994312	2.777112	-1.243373
34	1	0	-1.217978	-3.242689	-0.842348
35	1	0	-2.647673	-3.814376	0.047696
36	1	0	-3.767286	-1.905697	-0.908175
37	1	0	-6.388544	1.846202	0.026532
38	1	0	-5.260309	4.042581	-0.008978
39	1	0	-2.753077	4.105461	-0.030895
40	1	0	-1.499960	2.036788	-0.140133
41	1	0	3.834633	2.537666	1.836990
42	1	0	6.156969	2.297868	2.656344
43	1	0	7.614504	0.502540	1.730276
44	1	0	6.733956	-1.015003	-0.017134

- 2a-TS1-B-fix-UP-A-int

Method: opt(ts,calcfc,noeigen) freq b3lyp/6-311++g(d,p) scrf(solvent=Chlorof

SCF Done: E(RB3LYP) = -6223.12446587 A.U. after 1 cycles

Lowest frequency = -37.2478

Zero-point correction= 0.330357
(Hartree/Particle)

Thermal correction to Energy= 0.352279

Thermal correction to Enthalpy= 0.353223

Thermal correction to Gibbs Free Energy= 0.277296

Sum of electronic and zero-point Energies= -6222.794109

Sum of electronic and thermal Energies= -6222.772187

Sum of electronic and thermal Enthalpies= -6222.771243

Sum of electronic and thermal Free Energies= -6222.847170

Standard orientation:

Center Number	Atomic Number	Atomic Type	Coordinates (Angstroms) X	Y	Z
1	6	0	0.362569	2.855512	-0.713538
2	6	0	-0.979337	2.541783	-0.538674
3	6	0	-1.445912	1.221485	-0.693892
4	6	0	-0.496406	0.247462	-1.046596
5	6	0	0.895576	0.499880	-1.045748
6	6	0	1.279535	1.842047	-0.967023
7	8	0	-0.942605	-0.976990	-1.463288
8	6	0	-0.169337	-1.419589	-2.599469
9	6	0	1.265679	-1.544653	-2.201913
10	6	0	1.791308	-0.675874	-1.306882
11	8	0	-1.840869	3.553787	-0.217860
12	6	0	-3.174759	3.410747	-0.741812
13	6	0	-3.713498	2.032975	-0.504751
14	6	0	-2.887761	0.973334	-0.493615
15	6	0	-3.445783	-0.387451	-0.240938
16	6	0	-3.180536	-1.137648	0.911655
17	6	0	-3.745515	-2.392811	1.121444
18	6	0	-4.607905	-2.927454	0.167240
19	6	0	-4.905794	-2.198300	-0.981882
20	6	0	-4.330224	-0.946615	-1.175631
21	6	0	3.078733	-1.069374	-0.605632
22	6	0	3.397437	-2.454071	-0.684485
23	6	0	4.569051	-3.024542	-0.219135
24	6	0	5.517194	-2.229098	0.414468
25	6	0	5.219945	-0.891189	0.610793
26	6	0	4.024999	-0.327329	0.144314
27	35	0	-2.052148	-0.434760	2.305529
28	35	0	3.855956	1.494798	0.774790
29	1	0	0.676387	3.890495	-0.660560
30	1	0	2.305811	2.114656	-1.143265
31	1	0	-0.582380	-2.388326	-2.878823
32	1	0	-0.331452	-0.717288	-3.430426
33	1	0	1.802329	-2.401940	-2.581315
34	1	0	-3.768804	4.167719	-0.230663
35	1	0	-3.158271	3.657079	-1.814949
36	1	0	-4.776667	1.918873	-0.326467
37	1	0	-3.521068	-2.941651	2.026929
38	1	0	-5.046424	-3.905114	0.330304
39	1	0	-5.581089	-2.602836	-1.726915
40	1	0	-4.548594	-0.384079	-2.075903
41	1	0	2.666932	-3.124233	-1.108091
42	1	0	4.723168	-4.091109	-0.333043
43	1	0	6.446832	-2.641361	0.787978
44	1	0	5.912251	-0.261966	1.153420

2a-TS4-A-fix-UP-B-int

Method: opt(ts,calcfc,noeigen) freq b3lyp/6-311++g(d,p) scrf(solvent=Chlorof

SCF Done: E(RB3LYP) = -6223.11744683 A.U. after 1 cycles

Lowest frequency = -28.4483

Zero-point correction= 0.329895
(Hartree/Particle)

Thermal correction to Energy= 0.351921

Thermal correction to Enthalpy= 0.352865

Thermal correction to Gibbs Free Energy= 0.275906

Sum of electronic and zero-point Energies= -6222.787552

Sum of electronic and thermal Energies= -6222.765526
Sum of electronic and thermal Enthalpies= -6222.764582
Sum of electronic and thermal Free Energies= -6222.841541

Standard orientation:

Center Number	Atomic Number	Atomic Type	Coordinates (Angstroms) X	Y	Z
1	6	0	0.252815	2.443589	-1.551308
2	6	0	-0.993401	2.406113	-0.934224
3	6	0	-1.389998	1.337845	-0.092067
4	6	0	-0.333888	0.582663	0.444390
5	6	0	0.941579	0.574629	-0.170991
6	6	0	1.191671	1.477385	-1.208923
7	8	0	-0.564547	-0.122503	1.574845
8	6	0	0.579381	-0.527760	2.357164
9	6	0	1.723561	-0.983187	1.506719
10	6	0	1.922234	-0.410868	0.311380
11	8	0	-1.836675	3.476457	-1.062303
12	6	0	-2.497540	3.737033	0.209618
13	6	0	-3.336133	2.544592	0.549589
14	6	0	-2.846344	1.316263	0.268304
15	6	0	-3.856358	0.229451	-0.046084
16	6	0	-3.820506	-1.180165	0.066593
17	6	0	-4.931575	-1.978508	-0.229235
18	6	0	-6.116442	-1.429076	-0.694301
19	6	0	-6.171181	-0.056639	-0.904623
20	6	0	-5.066959	0.725276	-0.604663
21	6	0	3.062114	-0.801570	-0.569632
22	6	0	2.816850	-1.623057	-1.681539
23	6	0	3.842259	-2.035973	-2.525000
24	6	0	5.150804	-1.622741	-2.278565
25	6	0	5.424811	-0.797231	-1.192094
26	6	0	4.384583	-0.395137	-0.355176
27	35	0	-2.295698	-2.243466	0.560291
28	35	0	4.835063	0.776103	1.108569
29	1	0	0.488925	3.252005	-2.231507
30	1	0	2.161187	1.474736	-1.692489
31	1	0	0.871446	0.323207	2.990735
32	1	0	0.211345	-1.321672	3.007316
33	1	0	2.388751	-1.743705	1.898368
34	1	0	-1.725401	3.971237	0.953732
35	1	0	-3.112455	4.620573	0.046536
36	1	0	-4.372618	2.707305	0.815168
37	1	0	-4.849748	-3.049281	-0.100092
38	1	0	-6.960743	-2.070026	-0.918827
39	1	0	-7.056191	0.408194	-1.322977
40	1	0	-5.121080	1.776515	-0.842944
41	1	0	1.798132	-1.941851	-1.870899
42	1	0	3.621093	-2.677582	-3.369842
43	1	0	5.959424	-1.936394	-2.928491
44	1	0	6.435920	-0.464759	-0.996240

2a-TS1-B-fix-DOWN-A-int

Method: opt(ts,calcfc,noeigen) freq b3lyp/6-311++g(d,p) scrf(solvent=Chlorof
SCF Done: E(RB3LYP) = -6223.12454746 A.U. after 1 cycles
Lowest frequency = -37.0681

Zero-point correction= 0.330348
(Hartree/Particle)
Thermal correction to Energy= 0.352311
Thermal correction to Enthalpy= 0.353256
Thermal correction to Gibbs Free Energy= 0.276848
Sum of electronic and zero-point Energies= -6222.794199
Sum of electronic and thermal Energies= -6222.772236
Sum of electronic and thermal Enthalpies= -6222.771292
Sum of electronic and thermal Free Energies= -6222.847699

Standard orientation:

Center Number	Atomic Number	Atomic Type	Coordinates (Angstroms) X	Y	Z
1	6	0	0.750749	2.821300	-0.462131
2	6	0	-0.546574	2.576400	-0.029558
3	6	0	-1.059670	1.265075	0.028232
4	6	0	-0.207085	0.226759	-0.385736
5	6	0	1.168249	0.425752	-0.651009
6	6	0	1.587352	1.754613	-0.770253
7	8	0	-0.747276	-1.010999	-0.602132
8	6	0	-0.199293	-1.579065	-1.811702
9	6	0	1.280641	-1.723133	-1.662473
10	6	0	1.976963	-0.801369	-0.956930
11	8	0	-1.318102	3.644242	0.334280
12	6	0	-2.730024	3.494833	0.083585
13	6	0	-3.234413	2.168842	0.564016
14	6	0	-2.443696	1.085968	0.507386
15	6	0	-2.910141	-0.219981	1.061201
16	6	0	-3.914998	-1.004426	0.484356
17	6	0	-4.351296	-2.191807	1.068694
18	6	0	-3.775932	-2.622964	2.260782
19	6	0	-2.767378	-1.867200	2.856174
20	6	0	-2.345063	-0.685201	2.258455
21	6	0	3.360267	-1.180728	-0.461101
22	6	0	3.630451	-2.577933	-0.467552
23	6	0	4.854406	-3.148768	-0.166163
24	6	0	5.917235	-2.336962	0.214091
25	6	0	5.688213	-0.976941	0.336092
26	6	0	4.441037	-0.412000	0.038304
27	35	0	-4.722246	-0.508709	-1.196305
28	35	0	4.425439	1.463275	0.517731
29	1	0	1.091062	3.843983	-0.565935
30	1	0	2.571659	1.971417	-1.148059
31	1	0	-0.676670	-2.551882	-1.923506
32	1	0	-0.492646	-0.946087	-2.661732
33	1	0	1.722420	-2.628793	-2.051909
34	1	0	-3.205422	4.321992	0.609572
35	1	0	-2.909780	3.626151	-0.994626
36	1	0	-4.244004	2.111361	0.953805
37	1	0	-5.129096	-2.774788	0.592716
38	1	0	-4.115742	-3.546166	2.715710
39	1	0	-2.313264	-2.194919	3.784038
40	1	0	-1.562663	-0.095137	2.722479
41	1	0	2.822126	-3.254740	-0.693021
42	1	0	4.963490	-4.226105	-0.207587
43	1	0	6.890062	-2.749415	0.453193

44	1	0	6.479779	-0.329014	0.687203

- 2a-TS3-A-fix-DOWN-B-ext

Method: opt(ts,calcfc,noeigen) freq b3lyp/6-311++g(d,p) scrf(solvent=Chlorof
SCF Done: E(RB3LYP) = -6223.13525737 A.U. after 1 cycles
Lowest frequency = -39.5397

Zero-point correction= 0.330280
(Hartree/Particle)
Thermal correction to Energy= 0.352304
Thermal correction to Enthalpy= 0.353248
Thermal correction to Gibbs Free Energy= 0.276370
Sum of electronic and zero-point Energies= -6222.804977
Sum of electronic and thermal Energies= -6222.782953
Sum of electronic and thermal Enthalpies= -6222.782009
Sum of electronic and thermal Free Energies= -6222.858887

Standard orientation:

Center Number	Atomic Number	Atomic Type	Coordinates (Angstroms) X	Y	Z
1	6	0	-0.612676	-2.301828	-1.192385
2	6	0	0.662006	-2.062523	-0.692976
3	6	0	0.954450	-0.955201	0.141691
4	6	0	-0.161055	-0.313378	0.711046
5	6	0	-1.477529	-0.572700	0.270499
6	6	0	-1.670209	-1.531339	-0.728627
7	8	0	0.045907	0.471894	1.814040
8	6	0	-0.924043	1.495758	2.115292
9	6	0	-2.330091	1.055405	1.859252
10	6	0	-2.592937	0.100891	0.957679
11	8	0	1.653191	-2.957609	-0.980616
12	6	0	2.505222	-3.158043	0.173237
13	6	0	3.132978	-1.852077	0.543078
14	6	0	2.416805	-0.716581	0.411639
15	6	0	3.059103	0.635676	0.321709
16	6	0	4.424394	0.895850	0.024677
17	6	0	4.942270	2.190124	-0.034759
18	6	0	4.124504	3.297618	0.149253
19	6	0	2.768252	3.091511	0.364360
20	6	0	2.269499	1.798504	0.439996
21	6	0	-3.999214	-0.323033	0.682174
22	6	0	-4.585256	-1.325266	1.469519
23	6	0	-5.896582	-1.741896	1.261246
24	6	0	-6.655139	-1.162145	0.246028
25	6	0	-6.100109	-0.167387	-0.554819
26	6	0	-4.785886	0.238042	-0.330629
27	35	0	5.761435	-0.423912	-0.473496
28	35	0	-4.073086	1.621936	-1.465519
29	1	0	-0.768977	-3.126404	-1.876322
30	1	0	-2.673027	-1.735086	-1.083208
31	1	0	-0.759937	1.736461	3.166081
32	1	0	-0.686985	2.397158	1.527550
33	1	0	-3.122573	1.570502	2.390109
34	1	0	1.901945	-3.601099	0.978144
35	1	0	3.257879	-3.880989	-0.137794
36	1	0	4.181966	-1.853964	0.782769
37	1	0	5.992968	2.326408	-0.252759

38	1	0	4.543512	4.295376	0.095168
39	1	0	2.091094	3.930664	0.472832
40	1	0	1.218205	1.677515	0.611298
41	1	0	-3.991189	-1.775860	2.256402
42	1	0	-6.323363	-2.515171	1.889112
43	1	0	-7.677459	-1.477935	0.073584
44	1	0	-6.681602	0.291422	-1.343907

- 2a-TS2-B-fix-UP-A-ext

Method: opt(ts,calcfc,noeigen) freq b3lyp/6-311++g(d,p) scrf(solvent=Chlorof
SCF Done: E(RB3LYP) = -6223.13787337 A.U. after 1 cycles
Lowest frequency = -38.2596

Zero-point correction= 0.330618
(Hartree/Particle)
Thermal correction to Energy= 0.352574
Thermal correction to Enthalpy= 0.353519
Thermal correction to Gibbs Free Energy= 0.277295
Sum of electronic and zero-point Energies= -6222.807255
Sum of electronic and thermal Energies= -6222.785299
Sum of electronic and thermal Enthalpies= -6222.784355
Sum of electronic and thermal Free Energies= -6222.860579

Standard orientation:

Center Number	Atomic Number	Atomic Type	Coordinates (Angstroms) X	Y	Z
1	6	0	-0.374523	3.251928	0.442056
2	6	0	-1.441135	2.702041	-0.257967
3	6	0	-1.499830	1.316271	-0.518913
4	6	0	-0.371510	0.552162	-0.176586
5	6	0	0.816239	1.121469	0.343076
6	6	0	0.730399	2.464110	0.742760
7	8	0	-0.439235	-0.801560	-0.335631
8	6	0	0.226273	-1.490099	0.744177
9	6	0	1.659741	-1.072948	0.792211
10	6	0	1.993838	0.200560	0.496403
11	8	0	-2.485343	3.519398	-0.576368
12	6	0	-3.244227	3.104659	-1.728918
13	6	0	-3.613245	1.656190	-1.633737
14	6	0	-2.772343	0.784838	-1.052906
15	6	0	-3.176786	-0.649539	-0.949121
16	6	0	-3.573389	-1.257022	0.248126
17	6	0	-3.991810	-2.583367	0.301430
18	6	0	-4.024448	-3.339595	-0.868535
19	6	0	-3.648857	-2.759957	-2.078094
20	6	0	-3.234654	-1.431560	-2.111263
21	6	0	3.411122	0.629343	0.242642
22	6	0	3.727451	2.004355	0.166437
23	6	0	5.008271	2.504761	-0.024924
24	6	0	6.077230	1.631355	-0.169689
25	6	0	5.818936	0.266790	-0.170773
26	6	0	4.524639	-0.223133	0.006589
27	35	0	-3.588866	-0.253529	1.893277
28	35	0	4.466430	-2.150134	-0.240794
29	1	0	-0.411051	4.294599	0.731755
30	1	0	1.521065	2.916851	1.321955
31	1	0	-0.314969	-1.283538	1.678708

32	1	0	0.132200	-2.551248	0.517145
33	1	0	2.387869	-1.838624	0.992104
34	1	0	-4.124177	3.746355	-1.743507
35	1	0	-2.650106	3.313751	-2.631393
36	1	0	-4.575018	1.338245	-2.019226
37	1	0	-4.296842	-3.017626	1.244849
38	1	0	-4.349157	-4.372870	-0.828638
39	1	0	-3.676854	-3.338920	-2.993856
40	1	0	-2.932627	-0.981704	-3.049828
41	1	0	2.932395	2.722561	0.239165
42	1	0	5.158748	3.577218	-0.063871
43	1	0	7.089065	1.992438	-0.309677
44	1	0	6.627037	-0.433658	-0.333497

Compound 1b:

```
1b-anti-GS1
Method: opt freq b3lyp/6-31+g(d,p) scrf(solvent=Chloroform)
 SCF Done:  E(RB3LYP) =  -1461.81839842     A.U. after    1 cycles
 Lowest frequency =  14.0019

 Zero-point correction=                           0.499875
(Hartree/Particle)
 Thermal correction to Energy=                    0.528862
 Thermal correction to Enthalpy=                  0.529806
 Thermal correction to Gibbs Free Energy=         0.438317
 Sum of electronic and zero-point Energies=          -1461.318523
 Sum of electronic and thermal Energies=             -1461.289537
 Sum of electronic and thermal Enthalpies=           -1461.288592
 Sum of electronic and thermal Free Energies=        -1461.380081
```

Standard orientation:

Center Number	Atomic Number	Atomic Type	Coordinates (Angstroms) X	Y	Z
1	6	0	1.023082	3.039608	0.632382
2	6	0	1.057313	1.626073	0.622980
3	6	0	2.228538	0.954733	1.217919
4	6	0	3.060423	1.689030	1.980089
5	6	0	0.000000	3.744627	0.000000
6	6	0	0.000000	0.950656	0.000000
7	6	0	-1.057313	1.626073	-0.622980
8	6	0	-1.023082	3.039607	-0.632382
9	6	0	-3.060423	1.689030	-1.980089
10	6	0	-2.228538	0.954733	-1.217919
11	1	0	0.000000	4.829031	0.000000
12	1	0	3.948286	1.254617	2.430844
13	1	0	0.000000	-0.134723	0.000000
14	1	0	-3.948286	1.254617	-2.430844
15	8	0	-2.041510	3.766519	-1.183990
16	8	0	2.041510	3.766519	1.183990
17	6	0	-2.757150	3.131900	-2.266138
18	1	0	-3.667979	3.721269	-2.389120
19	1	0	-2.156936	3.238603	-3.185491
20	6	0	2.757150	3.131900	2.266138
21	1	0	3.667979	3.721269	2.389120
22	1	0	2.156935	3.238603	3.185491

23	6	0	-2.475777	-0.499122	-0.952897
24	6	0	-3.116264	-0.896506	0.269803
25	6	0	-2.084567	-1.456594	-1.884690
26	6	0	-3.536802	0.044345	1.254094
27	6	0	-3.355652	-2.288241	0.520946
28	6	0	-2.332855	-2.831685	-1.612044
29	6	0	-4.153150	-0.367305	2.417779
30	1	0	-3.371549	1.101875	1.078437
31	6	0	-3.995308	-2.680971	1.729732
32	6	0	-2.947317	-3.239353	-0.451588
33	6	0	-4.386369	-1.743864	2.661764
34	1	0	-4.464427	0.369345	3.152949
35	1	0	-4.172180	-3.739277	1.904632
36	1	0	-4.874622	-2.055509	3.580518
37	6	0	2.475777	-0.499122	0.952896
38	6	0	3.116264	-0.896506	-0.269803
39	6	0	2.084566	-1.456594	1.884690
40	6	0	3.536803	0.044345	-1.254094
41	6	0	3.355652	-2.288241	-0.520946
42	6	0	2.332854	-2.831685	1.612044
43	6	0	4.153151	-0.367305	-2.417778
44	1	0	3.371550	1.101875	-1.078437
45	6	0	3.995309	-2.680971	-1.729731
46	6	0	2.947317	-3.239353	0.451587
47	6	0	4.386371	-1.743863	-2.661763
48	1	0	4.464429	0.369345	-3.152948
49	1	0	4.172181	-3.739277	-1.904632
50	1	0	4.874624	-2.055509	-3.580517
51	1	0	3.123377	-4.295557	0.264202
52	1	0	2.019899	-3.571463	2.344220
53	1	0	-3.123377	-4.295557	-0.264202
54	1	0	-2.019900	-3.571463	-2.344220
55	6	0	1.409156	-1.085173	3.187205
56	1	0	0.880623	-1.946014	3.606209
57	1	0	2.140729	-0.751738	3.933342
58	1	0	0.689239	-0.272549	3.057880
59	6	0	-1.409157	-1.085173	-3.187206
60	1	0	-0.880621	-1.946013	-3.606208
61	1	0	-2.140732	-0.751743	-3.933343
62	1	0	-0.689244	-0.272546	-3.057882

1b-anti-GS2

Method: opt freq b3lyp/6-31+g(d,p) scrf(solvent=Chloroform)
SCF Done: E(RB3LYP) = -1461.81815460 A.U. after 1 cycles
Lowest frequency = 12.7558

Zero-point correction= 0.499697
(Hartree/Particle)
Thermal correction to Energy= 0.528776
Thermal correction to Enthalpy= 0.529720
Thermal correction to Gibbs Free Energy= 0.437218
Sum of electronic and zero-point Energies= -1461.318457
Sum of electronic and thermal Energies= -1461.289379
Sum of electronic and thermal Enthalpies= -1461.288435
Sum of electronic and thermal Free Energies= -1461.380936

Standard orientation:

Center Number	Atomic Number	Atomic Type	Coordinates (Angstroms) X	Y	Z
1	6	0	1.199526	3.277894	-0.088545
2	6	0	1.221955	1.864318	-0.114526
3	6	0	2.519904	1.193058	-0.316030
4	6	0	3.640419	1.928013	-0.185873
5	6	0	0.000000	3.982765	0.000000
6	6	0	0.000000	1.188622	0.000000
7	6	0	-1.221955	1.864318	0.114526
8	6	0	-1.199526	3.277894	0.088545
9	6	0	-3.640419	1.928013	0.185873
10	6	0	-2.519904	1.193058	0.316030
11	1	0	0.000000	5.067217	0.000000
12	1	0	4.624460	1.491315	-0.331809
13	1	0	0.000000	0.103306	0.000000
14	1	0	-4.624460	1.491314	0.331809
15	8	0	-2.347259	4.005278	0.240844
16	8	0	2.347259	4.005278	-0.240844
17	6	0	-3.563027	3.373370	-0.214707
18	1	0	-4.371502	3.960140	0.226090
19	1	0	-3.621016	3.486873	-1.310560
20	6	0	3.563027	3.373370	0.214707
21	1	0	4.371501	3.960141	-0.226090
22	1	0	3.621015	3.486874	1.310560
23	6	0	-2.566726	-0.259417	0.681707
24	6	0	-2.779266	-1.241613	-0.343085
25	6	0	-2.406291	-0.650597	2.009278
26	6	0	-2.927076	-0.897609	-1.719114
27	6	0	-2.839103	-2.630634	0.014182
28	6	0	-2.468331	-2.032708	2.337168
29	6	0	-3.130712	-1.866135	-2.679721
30	1	0	-2.878766	0.146343	-2.009253
31	6	0	-3.055184	-3.605201	-1.000300
32	6	0	-2.679846	-2.994752	1.376483
33	6	0	-3.199176	-3.235739	-2.319842
34	1	0	-3.238499	-1.578156	-3.721619
35	1	0	-3.102406	-4.652578	-0.712842
36	1	0	-3.362698	-3.988862	-3.085132
37	6	0	2.566726	-0.259417	-0.681707
38	6	0	2.779266	-1.241613	0.343085
39	6	0	2.406291	-0.650597	-2.009278
40	6	0	2.927076	-0.897609	1.719114
41	6	0	2.839103	-2.630634	-0.014182
42	6	0	2.468331	-2.032708	-2.337168
43	6	0	3.130712	-1.866134	2.679721
44	1	0	2.878766	0.146343	2.009253
45	6	0	3.055185	-3.605201	1.000300
46	6	0	2.679847	-2.994751	-1.376483
47	6	0	3.199176	-3.235739	2.319842
48	1	0	3.238499	-1.578156	3.721619
49	1	0	3.102406	-4.652577	0.712842
50	1	0	3.362699	-3.988861	3.085133
51	1	0	2.723902	-4.045550	-1.651126
52	1	0	2.346154	-2.326944	-3.376214
53	1	0	-2.723902	-4.045550	1.651126
54	1	0	-2.346154	-2.326944	3.376214
55	6	0	2.182265	0.358987	-3.112988
56	1	0	2.040565	-0.141551	-4.074384
57	1	0	1.298972	0.977375	-2.919982

58	1	0	3.033255	1.042542	-3.208425
59	6	0	-2.182265	0.358987	3.112988
60	1	0	-2.040566	-0.141551	4.074384
61	1	0	-1.298972	0.977374	2.919982
62	1	0	-3.033255	1.042542	3.208424

1b-anti-GS3

Method: opt freq b3lyp/6-31+g(d,p) scrf(solvent=Chloroform)
SCF Done: E(RB3LYP) = -1461.81827116 A.U. after 1 cycles
Lowest frequency = 10.8279

Zero-point correction= 0.499717
(Hartree/Particle)
Thermal correction to Energy= 0.528773
Thermal correction to Enthalpy= 0.529717
Thermal correction to Gibbs Free Energy= 0.437397
Sum of electronic and zero-point Energies= -1461.318554
Sum of electronic and thermal Energies= -1461.289498
Sum of electronic and thermal Enthalpies= -1461.288554
Sum of electronic and thermal Free Energies= -1461.380874

Standard orientation:

Center Number	Atomic Number	Atomic Type	Coordinates (Angstroms) X	Y	Z
1	6	0	-0.754183	3.279893	-0.466009
2	6	0	-0.986965	1.898407	-0.275330
3	6	0	-2.304478	1.355770	-0.656994
4	6	0	-3.301787	2.226544	-0.901263
5	6	0	0.490144	3.849641	-0.199693
6	6	0	0.071797	1.117415	0.205838
7	6	0	1.328735	1.659485	0.503812
8	6	0	1.514388	3.046184	0.299496
9	6	0	3.569239	1.517171	1.408251
10	6	0	2.486387	0.861376	0.950419
11	1	0	0.653956	4.909228	-0.361762
12	1	0	-4.291190	1.886873	-1.194697
13	1	0	-0.080207	0.050103	0.331537
14	1	0	4.458434	0.984812	1.734085
15	8	0	2.729698	3.634523	0.514846
16	8	0	-1.720962	4.093391	-0.988248
17	6	0	3.561493	3.014902	1.520053
18	1	0	3.203106	3.339376	2.511619
19	1	0	4.556190	3.438308	1.367248
20	6	0	-3.086410	3.702691	-0.727294
21	1	0	-3.688413	4.285447	-1.427485
22	1	0	-3.348258	4.025944	0.294553
23	6	0	2.444117	-0.634277	0.869462
24	6	0	2.760084	-1.285048	-0.371283
25	6	0	2.098563	-1.385698	1.989183
26	6	0	3.119784	-0.561582	-1.545264
27	6	0	2.723483	-2.716987	-0.445719
28	6	0	2.068065	-2.805436	1.889635
29	6	0	3.422697	-1.215473	-2.721748
30	1	0	3.159349	0.521707	-1.506611
31	6	0	3.042790	-3.361982	-1.673092
32	6	0	2.370186	-3.454139	0.715601
33	6	0	3.384911	-2.630691	-2.790171

34	1	0	3.693979	-0.641559	-3.603273
35	1	0	3.012592	-4.448000	-1.713074
36	1	0	3.627518	-3.134079	-3.721512
37	6	0	-2.498056	-0.124575	-0.786734
38	6	0	-3.045988	-0.865766	0.313498
39	6	0	-2.144300	-0.774822	-1.967047
40	6	0	-3.405010	-0.253210	1.550266
41	6	0	-3.237677	-2.282371	0.182761
42	6	0	-2.344575	-2.178684	-2.071777
43	6	0	-3.927593	-0.994403	2.589497
44	1	0	-3.263321	0.815490	1.669339
45	6	0	-3.783388	-3.017959	1.272166
46	6	0	-2.874374	-2.912740	-1.035659
47	6	0	-4.122973	-2.391912	2.451725
48	1	0	-4.191752	-0.504207	3.522263
49	1	0	-3.926377	-4.089347	1.155658
50	1	0	-4.538007	-2.964183	3.276264
51	1	0	-3.017841	-3.985292	-1.138803
52	1	0	-2.068818	-2.675708	-2.998047
53	1	0	2.337020	-4.539346	0.662446
54	1	0	1.792877	-3.382542	2.768567
55	6	0	-1.562599	-0.023741	-3.143789
56	1	0	-1.328745	-0.707536	-3.963834
57	1	0	-0.641384	0.501483	-2.869329
58	1	0	-2.260053	0.732718	-3.519963
59	6	0	1.763205	-0.743179	3.317843
60	1	0	1.199608	-1.435641	3.949413
61	1	0	1.169492	0.166703	3.194526
62	1	0	2.672079	-0.462892	3.864242

1b-syn-GS1

Method: opt freq b3lyp/6-31+g(d,p) scrf(solvent=Chloroform)
SCF Done: E(RB3LYP) = -1461.81811921 A.U. after 1 cycles
Lowest frequency = 14.0986

Zero-point correction= 0.499764
(Hartree/Particle)
Thermal correction to Energy= 0.528809
Thermal correction to Enthalpy= 0.529753
Thermal correction to Gibbs Free Energy= 0.437825
Sum of electronic and zero-point Energies= -1461.318355
Sum of electronic and thermal Energies= -1461.289310
Sum of electronic and thermal Enthalpies= -1461.288366
Sum of electronic and thermal Free Energies= -1461.380294

Standard orientation:

Center Number	Atomic Number	Atomic Type	Coordinates (Angstroms) X	Y	Z
1	6	0	1.514444	3.062890	-0.287893
2	6	0	1.358042	1.730093	0.158163
3	6	0	2.560104	1.001680	0.606786
4	6	0	3.681198	1.710292	0.837331
5	6	0	0.444545	3.779181	-0.822369
6	6	0	0.077465	1.165449	0.095222
7	6	0	-1.025994	1.865486	-0.409856
8	6	0	-0.810783	3.176878	-0.892984
9	6	0	-3.332265	2.004110	-1.130420

10	6	0	-2.404886	1.341281	-0.414010
11	1	0	0.586972	4.793334	-1.178992
12	1	0	4.601932	1.227596	1.152670
13	1	0	-0.063841	0.149158	0.448913
14	1	0	-4.366903	1.674234	-1.161946
15	8	0	-1.847331	3.934344	-1.363526
16	8	0	2.742072	3.665062	-0.295826
17	6	0	-2.947744	3.203947	-1.947850
18	1	0	-3.763797	3.925963	-2.017408
19	1	0	-2.662970	2.913827	-2.973364
20	6	0	3.677872	3.206192	0.703906
21	1	0	4.648854	3.586871	0.380826
22	1	0	3.424632	3.691752	1.661561
23	6	0	-2.749838	0.126054	0.392374
24	6	0	-2.771367	-1.162500	-0.239271
25	6	0	-3.050335	0.251192	1.747062
26	6	0	-2.452156	-1.348367	-1.616488
27	6	0	-3.117626	-2.320698	0.534840
28	6	0	-3.389264	-0.911331	2.492417
29	6	0	-2.481842	-2.600609	-2.193472
30	1	0	-2.180849	-0.485924	-2.215562
31	6	0	-3.143848	-3.598374	-0.091656
32	6	0	-3.425220	-2.157834	1.910678
33	6	0	-2.834250	-3.739850	-1.426763
34	1	0	-2.230362	-2.716491	-3.243814
35	1	0	-3.412399	-4.465288	0.506895
36	1	0	-2.855973	-4.720325	-1.893434
37	6	0	2.517407	-0.486425	0.776189
38	6	0	2.679235	-1.333752	-0.372368
39	6	0	2.320518	-1.042228	2.037132
40	6	0	2.886421	-0.814516	-1.683112
41	6	0	2.641195	-2.758099	-0.209081
42	6	0	2.286296	-2.459020	2.173263
43	6	0	3.042949	-1.653932	-2.766757
44	1	0	2.925711	0.260078	-1.825509
45	6	0	2.807103	-3.597270	-1.345927
46	6	0	2.440955	-3.292971	1.091271
47	6	0	3.003276	-3.060828	-2.600283
48	1	0	3.199359	-1.233767	-3.756227
49	1	0	2.776755	-4.674908	-1.205727
50	1	0	3.129179	-3.711351	-3.460871
51	1	0	2.406784	-4.371897	1.218953
52	1	0	2.127669	-2.882747	3.161553
53	1	0	-3.687880	-3.032394	2.500403
54	1	0	-3.626886	-0.802080	3.547370
55	6	0	-3.042839	1.594838	2.441784
56	1	0	-2.087236	2.113131	2.309313
57	1	0	-3.819815	2.255713	2.041121
58	1	0	-3.219584	1.478361	3.514303
59	6	0	2.150666	-0.190660	3.276824
60	1	0	1.650538	-0.757084	4.067605
61	1	0	3.121080	0.136682	3.669891
62	1	0	1.564503	0.710525	3.078204

1b-syn-GS2
Method: opt freq b3lyp/6-31+g(d,p) scrf(solvent=Chloroform)
SCF Done: E(RB3LYP) = -1461.81785909 A.U. after 2 cycles
Lowest frequency = 9.2212

Zero-point correction= 0.499697
(Hartree/Particle)
Thermal correction to Energy= 0.528740
Thermal correction to Enthalpy= 0.529685
Thermal correction to Gibbs Free Energy= 0.437623
Sum of electronic and zero-point Energies= -1461.318162
Sum of electronic and thermal Energies= -1461.289119
Sum of electronic and thermal Enthalpies= -1461.288175
Sum of electronic and thermal Free Energies= -1461.380236

Standard orientation:

Center Number	Atomic Number	Atomic Type	Coordinates (Angstroms) X	Y	Z
1	6	0	1.202799	3.082684	-0.771864
2	6	0	1.227673	1.854057	-0.072525
3	6	0	2.539835	1.240633	0.208385
4	6	0	3.641685	1.994912	0.037776
5	6	0	0.000042	3.685418	-1.137517
6	6	0	0.000017	1.264993	0.256955
7	6	0	-1.227625	1.854094	-0.072505
8	6	0	-1.202728	3.082721	-0.771845
9	6	0	-3.641636	1.995008	0.037810
10	6	0	-2.539803	1.240709	0.208430
11	1	0	0.000052	4.622230	-1.683616
12	1	0	4.635568	1.593495	0.214864
13	1	0	0.000005	0.307010	0.767344
14	1	0	-4.635526	1.593621	0.214925
15	8	0	-2.359570	3.687266	-1.179102
16	8	0	2.359659	3.687187	-1.179141
17	6	0	-3.525164	3.437338	-0.363903
18	1	0	-3.474919	4.094974	0.520451
19	1	0	-4.372740	3.758271	-0.972871
20	6	0	3.525221	3.437256	-0.363894
21	1	0	4.372820	3.758226	-0.972808
22	1	0	3.474915	4.094860	0.520481
23	6	0	-2.624652	-0.186396	0.657714
24	6	0	-2.614750	-1.238930	-0.319243
25	6	0	-2.715364	-0.486950	2.013951
26	6	0	-2.525137	-0.983675	-1.718286
27	6	0	-2.705827	-2.603366	0.113617
28	6	0	-2.801540	-1.848930	2.418702
29	6	0	-2.525062	-2.016115	-2.633088
30	1	0	-2.459178	0.042040	-2.064794
31	6	0	-2.704324	-3.645862	-0.854614
32	6	0	-2.798366	-2.875786	1.504251
33	6	0	-2.615770	-3.362671	-2.200405
34	1	0	-2.453996	-1.796162	-3.694477
35	1	0	-2.774906	-4.674939	-0.511142
36	1	0	-2.615083	-4.166683	-2.930602
37	6	0	2.624671	-0.186491	0.657618
38	6	0	2.614640	-1.238986	-0.319391
39	6	0	2.715538	-0.487112	2.013824
40	6	0	2.524856	-0.983668	-1.718408
41	6	0	2.705769	-2.603440	0.113393
42	6	0	2.801771	-1.849118	2.418500
43	6	0	2.524658	-2.016066	-2.633260
44	1	0	2.458866	0.042064	-2.064863
45	6	0	2.704137	-3.645892	-0.854883

46	6	0	2.798490	-2.875927	1.504006
47	6	0	2.615411	-3.362640	-2.200651
48	1	0	2.453461	-1.796061	-3.694630
49	1	0	2.774760	-4.674984	-0.511466
50	1	0	2.614626	-4.166620	-2.930884
51	1	0	2.863099	-3.908826	1.836468
52	1	0	2.868405	-2.073201	3.480026
53	1	0	-2.862932	-3.908668	1.836772
54	1	0	-2.868053	-2.072966	3.480244
55	6	0	-2.739333	0.590048	3.077183
56	1	0	-2.044731	1.404062	2.852068
57	1	0	-3.737400	1.035694	3.168817
58	1	0	-2.474205	0.174164	4.053380
59	6	0	2.739670	0.589782	3.077155
60	1	0	2.473367	0.174065	4.053108
61	1	0	3.738119	1.034411	3.169650
62	1	0	2.046062	1.404496	2.851559

1b-syn-GS3

Method: opt freq b3lyp/6-31+g(d,p) scrf(solvent=Chloroform)
SCF Done: E(RB3LYP) = -1461.81801029 A.U. after 1 cycles
Lowest frequency = 14.0369

Zero-point correction= 0.499690
(Hartree/Particle)
Thermal correction to Energy= 0.528762
Thermal correction to Enthalpy= 0.529707
Thermal correction to Gibbs Free Energy= 0.437517
Sum of electronic and zero-point Energies= -1461.318320
Sum of electronic and thermal Energies= -1461.289248
Sum of electronic and thermal Enthalpies= -1461.288304
Sum of electronic and thermal Free Energies= -1461.380494

Standard orientation:

Center Number	Atomic Number	Atomic Type	Coordinates (Angstroms) X	Y	Z
1	6	0	1.203140	3.240139	-0.500628
2	6	0	1.227814	1.850967	-0.237869
3	6	0	2.539455	1.203700	-0.045346
4	6	0	3.642461	1.888230	-0.402179
5	6	0	0.000403	3.936195	-0.612967
6	6	0	0.000133	1.187872	-0.110345
7	6	0	-1.227419	1.851202	-0.237898
8	6	0	-1.202471	3.240368	-0.500665
9	6	0	-3.642052	1.888925	-0.402285
10	6	0	-2.539190	1.204193	-0.045393
11	1	0	0.000507	5.003802	-0.802961
12	1	0	4.636150	1.468079	-0.274082
13	1	0	0.000030	0.126279	0.115227
14	1	0	-4.635828	1.468978	-0.274193
15	8	0	-2.359103	3.964830	-0.580572
16	8	0	2.359910	3.964385	-0.580493
17	6	0	-3.525766	3.245600	-1.035783
18	1	0	-4.372650	3.886055	-0.781173
19	1	0	-3.476776	3.169805	-2.135266
20	6	0	3.526471	3.244949	-1.035636

21	1	0	3.477556	3.169202	-2.135126
22	1	0	4.373457	3.885236	-0.780939
23	6	0	-2.622322	-0.164484	0.559661
24	6	0	-2.698349	-1.313883	-0.296523
25	6	0	-2.629028	-0.316505	1.944320
26	6	0	-2.676991	-1.214889	-1.718788
27	6	0	-2.792865	-2.621870	0.286850
28	6	0	-2.723001	-1.622985	2.497969
29	6	0	-2.751596	-2.339281	-2.513976
30	1	0	-2.601146	-0.235029	-2.177650
31	6	0	-2.872089	-3.761890	-0.561392
32	6	0	-2.804246	-2.741583	1.700973
33	6	0	-2.853001	-3.628287	-1.932595
34	1	0	-2.730842	-2.237660	-3.595306
35	1	0	-2.946347	-4.745463	-0.104349
36	1	0	-2.912650	-4.505945	-2.569567
37	6	0	2.622303	-0.165021	0.559646
38	6	0	2.698051	-1.314397	-0.296595
39	6	0	2.629017	-0.317110	1.944297
40	6	0	2.676673	-1.215330	-1.718853
41	6	0	2.792302	-2.622432	0.286712
42	6	0	2.722718	-1.623637	2.497882
43	6	0	2.751013	-2.339699	-2.514098
44	1	0	2.601027	-0.235431	-2.177667
45	6	0	2.871254	-3.762428	-0.561586
46	6	0	2.803697	-2.742215	1.700830
47	6	0	2.852157	-3.628755	-1.932782
48	1	0	2.730251	-2.238022	-3.595423
49	1	0	2.945314	-4.746039	-0.104592
50	1	0	2.911600	-4.506396	-2.569798
51	1	0	2.874197	-3.731117	2.146646
52	1	0	2.731020	-1.731224	3.579358
53	1	0	-2.874951	-3.730448	2.146838
54	1	0	-2.731300	-1.730519	3.579450
55	6	0	-2.559140	0.873849	2.875105
56	1	0	-1.718562	1.531087	2.629963
57	1	0	-3.467790	1.483528	2.811089
58	1	0	-2.445011	0.549251	3.912801
59	6	0	2.559430	0.873214	2.875143
60	1	0	2.445022	0.548593	3.912803
61	1	0	3.468325	1.482549	2.811309
62	1	0	1.719141	1.530787	2.629913

1b-TS2-ext
Method: opt(ts,calcfc,noeigen) b3lyp/6-31+g(d,p) freq scrf(solvent=Chlorofor
SCF Done: E(RB3LYP) = -1461.76777077 A.U. after 1 cycles
Lowest frequency = -20.6403

Zero-point correction= 0.500613
(Hartree/Particle)
Thermal correction to Energy= 0.528008
Thermal correction to Enthalpy= 0.528952
Thermal correction to Gibbs Free Energy= 0.443397
Sum of electronic and zero-point Energies= -1461.267158
Sum of electronic and thermal Energies= -1461.239763
Sum of electronic and thermal Enthalpies= -1461.238819
Sum of electronic and thermal Free Energies= -1461.324373

Standard orientation:

Center Number	Atomic Number	Atomic Type	Coordinates (Angstroms) X	Y	Z
1	6	0	-1.607940	2.922058	-0.502825
2	6	0	-1.458028	1.579572	-0.075487
3	6	0	-2.738761	0.832302	0.207821
4	6	0	-3.687221	1.634722	0.758670
5	6	0	-0.523987	3.721886	-0.860257
6	6	0	-0.141341	1.184521	0.224046
7	6	0	0.978817	1.987736	-0.045234
8	6	0	0.764715	3.247135	-0.648024
9	6	0	3.334419	2.534543	0.132735
10	6	0	2.372960	1.599400	0.254685
11	1	0	-0.687756	4.730666	-1.222295
12	1	0	-4.608651	1.234620	1.158704
13	1	0	0.034812	0.232981	0.707546
14	1	0	4.373706	2.299268	0.345718
15	8	0	1.808550	4.039963	-1.043577
16	8	0	-2.851914	3.503020	-0.498001
17	6	0	3.008417	3.948252	-0.249507
18	1	0	3.794808	4.390744	-0.865261
19	1	0	2.885202	4.582292	0.645254
20	6	0	-3.553849	3.123838	0.708646
21	1	0	-3.019341	3.548506	1.574270
22	1	0	-4.534872	3.599647	0.644108
23	6	0	2.726847	0.211350	0.698359
24	6	0	3.146813	-0.759522	-0.272372
25	6	0	2.703530	-0.113702	2.052197
26	6	0	3.170235	-0.484647	-1.670940
27	6	0	3.563824	-2.060376	0.166602
28	6	0	3.106607	-1.415779	2.460980
29	6	0	3.583809	-1.437462	-2.579028
30	1	0	2.857539	0.492933	-2.021612
31	6	0	3.993517	-3.018216	-0.794113
32	6	0	3.530656	-2.358535	1.554247
33	6	0	4.004264	-2.717859	-2.139308
34	1	0	3.588248	-1.205114	-3.640090
35	1	0	4.311700	-3.997612	-0.445545
36	1	0	4.332017	-3.457975	-2.863403
37	6	0	-3.135874	-0.598128	-0.053076
38	6	0	-2.233737	-1.744186	-0.093428
39	6	0	-4.511301	-0.890401	-0.204026
40	6	0	-0.886199	-1.682291	-0.542999
41	6	0	-2.739788	-3.058457	0.200494
42	6	0	-4.983028	-2.212717	0.034333
43	6	0	-0.063919	-2.793111	-0.561804
44	1	0	-0.509899	-0.761043	-0.958398
45	6	0	-1.858989	-4.173655	0.240751
46	6	0	-4.139221	-3.249489	0.334366
47	6	0	-0.534244	-4.047625	-0.115606
48	1	0	0.948257	-2.698693	-0.940686
49	1	0	-2.269811	-5.143546	0.509736
50	1	0	0.126822	-4.909145	-0.105082
51	1	0	-4.525373	-4.237058	0.571752
52	1	0	-6.055150	-2.386441	-0.014309
53	1	0	3.840543	-3.344747	1.890335
54	1	0	3.081939	-1.659370	3.519893
55	6	0	-5.587505	0.065813	-0.699342
56	1	0	-6.187908	-0.460868	-1.450227

57	1	0	-6.282109	0.383149	0.087535
58	1	0	-5.173864	0.962498	-1.161736
59	6	0	2.299609	0.889503	3.110759
60	1	0	2.071886	0.385683	4.054192
61	1	0	3.107840	1.605581	3.303835
62	1	0	1.421679	1.470343	2.814489

1b-TS1-int

Method: opt(ts,calcfc,noeigen) b3lyp/6-31+g(d,p) freq scrf(solvent=Chlorofor
SCF Done: E(RB3LYP) = -1461.75506826 A.U. after 1 cycles
Lowest frequency = -49.4085

Zero-point correction= 0.500591
(Hartree/Particle)
Thermal correction to Energy= 0.528142
Thermal correction to Enthalpy= 0.529086
Thermal correction to Gibbs Free Energy= 0.442738
Sum of electronic and zero-point Energies= -1461.254477
Sum of electronic and thermal Energies= -1461.226927
Sum of electronic and thermal Enthalpies= -1461.225982
Sum of electronic and thermal Free Energies= -1461.312330

Standard orientation:

Center Number	Atomic Number	Atomic Type	Coordinates (Angstroms) X	Y	Z
1	6	0	-0.930627	2.806988	-0.534523
2	6	0	-0.843295	1.584193	0.190611
3	6	0	-2.158442	1.010901	0.694364
4	6	0	-3.053976	1.989583	0.999449
5	6	0	0.151512	3.360149	-1.219709
6	6	0	0.465652	1.102587	0.364465
7	6	0	1.595553	1.670246	-0.243710
8	6	0	1.409033	2.788669	-1.082983
9	6	0	3.988980	1.895922	-0.551389
10	6	0	2.969471	1.151759	-0.082494
11	1	0	0.018777	4.275434	-1.785437
12	1	0	-4.079734	1.790301	1.246595
13	1	0	0.645112	0.266991	1.015846
14	1	0	5.017900	1.557464	-0.465258
15	8	0	2.445185	3.338082	-1.786688
16	8	0	-2.086745	3.535893	-0.540279
17	6	0	3.747250	3.241326	-1.171798
18	1	0	4.458826	3.449287	-1.973877
19	1	0	3.838373	4.046623	-0.423151
20	6	0	-2.730372	3.427333	0.749824
21	1	0	-2.061018	3.868757	1.504816
22	1	0	-3.635279	4.032661	0.684305
23	6	0	3.224211	-0.170237	0.576879
24	6	0	3.250673	-1.365434	-0.218988
25	6	0	3.458269	-0.233696	1.947907
26	6	0	3.010416	-1.355209	-1.623994
27	6	0	3.526915	-2.625201	0.409546
28	6	0	3.725534	-1.496023	2.549073
29	6	0	3.042841	-2.520611	-2.361713
30	1	0	2.801331	-0.411870	-2.116697
31	6	0	3.557986	-3.808652	-0.379776

32	6	0	3.762624	-2.654924	1.809627
33	6	0	3.321110	-3.762060	-1.736774
34	1	0	2.854630	-2.487149	-3.430986
35	1	0	3.773073	-4.755035	0.110074
36	1	0	3.347710	-4.671990	-2.329127
37	6	0	-2.610797	-0.422681	0.442410
38	6	0	-4.042223	-0.727170	0.283540
39	6	0	-1.753770	-1.494250	0.130632
40	6	0	-5.082278	-0.175020	1.086951
41	6	0	-4.456311	-1.736215	-0.651313
42	6	0	-2.198414	-2.528395	-0.748349
43	6	0	-6.419798	-0.455017	0.864475
44	1	0	-4.835301	0.401806	1.967037
45	6	0	-5.835531	-1.960609	-0.907817
46	6	0	-3.475522	-2.584592	-1.232889
47	6	0	-6.813510	-1.311472	-0.184547
48	1	0	-7.168021	-0.030113	1.527620
49	1	0	-6.103423	-2.699490	-1.658767
50	1	0	-7.865063	-1.502926	-0.375487
51	1	0	-3.771423	-3.330380	-1.965227
52	1	0	-1.469009	-3.276011	-1.049964
53	1	0	3.969937	-3.607664	2.290244
54	1	0	3.905185	-1.534552	3.620366
55	6	0	-0.395351	-1.766375	0.739339
56	1	0	-0.363921	-2.831771	0.993865
57	1	0	0.440578	-1.584074	0.059889
58	1	0	-0.239688	-1.220341	1.669481
59	6	0	3.464303	1.003732	2.819200
60	1	0	3.338781	0.736083	3.872132
61	1	0	4.413064	1.546545	2.727317
62	1	0	2.668369	1.702335	2.546974

Compound 2b:

2b-anti-GS1
Method: opt freq b3lyp/6-31+g(d,p) scrf(solvent=Chloroform)
SCF Done: E(RB3LYP) = -1461.81237347 A.U. after 2 cycles
Lowest frequency = 13.1670

Zero-point correction= 0.499907
(Hartree/Particle)
Thermal correction to Energy= 0.528815
Thermal correction to Enthalpy= 0.529760
Thermal correction to Gibbs Free Energy= 0.438488
Sum of electronic and zero-point Energies= -1461.312466
Sum of electronic and thermal Energies= -1461.283558
Sum of electronic and thermal Enthalpies= -1461.282614
Sum of electronic and thermal Free Energies= -1461.373886

Standard orientation:

Center Number	Atomic Number	Atomic Type	Coordinates (Angstroms) X	Y	Z
1	6	0	-0.837277	2.942034	0.106261
2	6	0	0.547728	2.777028	0.087890
3	6	0	1.150121	1.494826	0.034066
4	6	0	0.286209	0.388910	-0.089124

5	6	0	-1.120359	0.532935	-0.120907
6	6	0	-1.657943	1.822062	0.000144
7	8	0	0.848952	-0.842343	-0.266266
8	6	0	0.082673	-1.969446	0.204472
9	6	0	-1.357226	-1.877105	-0.208277
10	6	0	-1.942494	-0.672713	-0.343006
11	8	0	1.316851	3.902028	0.201523
12	6	0	2.614624	3.815000	-0.423151
13	6	0	3.320933	2.556880	-0.014694
14	6	0	2.621046	1.423065	0.188765
15	1	0	-1.250815	3.942018	0.178807
16	1	0	-2.735536	1.949452	-0.021292
17	1	0	0.574627	-2.844736	-0.224081
18	1	0	0.184735	-2.026975	1.301398
19	1	0	-1.909486	-2.799162	-0.366662
20	1	0	2.478069	3.872737	-1.515977
21	1	0	3.152004	4.707700	-0.098937
22	1	0	4.397528	2.584981	0.126540
23	6	0	-3.382788	-0.533305	-0.733549
24	6	0	-4.403003	-0.621643	0.272203
25	6	0	-3.725831	-0.316876	-2.066671
26	6	0	-4.106023	-0.820062	1.652862
27	6	0	-5.781412	-0.496704	-0.109174
28	6	0	-5.098200	-0.197196	-2.418985
29	6	0	-5.109833	-0.897417	2.595567
30	1	0	-3.070095	-0.912017	1.960513
31	6	0	-6.793840	-0.585872	0.887301
32	6	0	-6.096977	-0.284837	-1.476471
33	1	0	-5.355256	-0.034757	-3.462391
34	6	0	-6.469906	-0.782581	2.211993
35	1	0	-4.857289	-1.047083	3.641437
36	1	0	-7.833121	-0.493924	0.581828
37	1	0	-7.139459	-0.190982	-1.769728
38	1	0	-7.251376	-0.847976	2.963370
39	6	0	3.325804	0.189404	0.671303
40	6	0	4.119445	-0.584445	-0.240767
41	6	0	3.249254	-0.169914	2.014793
42	6	0	4.217764	-0.276696	-1.629273
43	6	0	4.834184	-1.730668	0.244500
44	6	0	3.963938	-1.312236	2.469680
45	6	0	4.986952	-1.042681	-2.480519
46	1	0	3.672171	0.574987	-2.019538
47	6	0	5.624475	-2.497224	-0.657489
48	6	0	4.733247	-2.071388	1.618853
49	1	0	3.902293	-1.578644	3.521830
50	6	0	5.704147	-2.163422	-1.992030
51	1	0	5.041222	-0.787318	-3.535051
52	1	0	6.165484	-3.357713	-0.271523
53	1	0	5.276139	-2.936654	1.990761
54	1	0	6.309631	-2.757168	-2.670703
55	6	0	2.426022	0.617536	3.010081
56	1	0	2.578339	1.695468	2.898940
57	1	0	1.352878	0.432670	2.879143
58	1	0	2.686637	0.339147	4.034911
59	6	0	-2.675777	-0.218608	-3.150849
60	1	0	-1.958101	0.583051	-2.944996
61	1	0	-2.098849	-1.146234	-3.233930
62	1	0	-3.136874	-0.018166	-4.121511

2b-anti-GS2

Method: opt freq b3lyp/6-31+g(d,p) scrf(solvent=Chloroform)
SCF Done: E(RB3LYP) = -1461.81168165 A.U. after 1 cycles
Lowest frequency = 10.3619

Zero-point correction= 0.499740
(Hartree/Particle)
Thermal correction to Energy= 0.528766
Thermal correction to Enthalpy= 0.529710
Thermal correction to Gibbs Free Energy= 0.437425
Sum of electronic and zero-point Energies= -1461.311941
Sum of electronic and thermal Energies= -1461.282915
Sum of electronic and thermal Enthalpies= -1461.281971
Sum of electronic and thermal Free Energies= -1461.374257

Standard orientation:

Center Number	Atomic Number	Atomic Type	Coordinates (Angstroms) X	Y	Z
1	6	0	0.638866	-1.973951	-2.102445
2	6	0	-0.700507	-2.090960	-1.731758
3	6	0	-1.237256	-1.397725	-0.617674
4	6	0	-0.331688	-0.648586	0.162492
5	6	0	1.030563	-0.510871	-0.196017
6	6	0	1.489314	-1.178748	-1.339559
7	8	0	-0.821014	0.019820	1.247136
8	6	0	0.107842	0.267636	2.321593
9	6	0	1.427164	0.774943	1.819458
10	6	0	1.882278	0.379577	0.616749
11	8	0	-1.505145	-2.856608	-2.530907
12	6	0	-2.626842	-3.470557	-1.865062
13	6	0	-3.366195	-2.471508	-1.027262
14	6	0	-2.701278	-1.480977	-0.400579
15	1	0	0.995452	-2.510773	-2.974734
16	1	0	2.530381	-1.077479	-1.628779
17	1	0	0.229625	-0.666119	2.896970
18	1	0	-0.391406	0.995598	2.964071
19	1	0	2.004414	1.437469	2.458147
20	1	0	-2.254040	-4.314684	-1.260731
21	1	0	-3.251425	-3.874098	-2.663932
22	1	0	-4.445354	-2.561713	-0.942771
23	6	0	3.204421	0.840249	0.081732
24	6	0	4.402143	0.142915	0.454942
25	6	0	3.266875	1.938385	-0.773623
26	6	0	4.391953	-0.998231	1.310154
27	6	0	5.667282	0.593532	-0.052260
28	6	0	4.532209	2.365324	-1.262325
29	6	0	5.560708	-1.647923	1.648253
30	1	0	3.444939	-1.355489	1.699871
31	6	0	6.855639	-0.096203	0.319139
32	6	0	5.696825	1.719617	-0.915508
33	1	0	4.570658	3.226336	-1.924449
34	6	0	6.808371	-1.193235	1.151516
35	1	0	5.526071	-2.516113	2.300257
36	1	0	7.806305	0.260126	-0.069625
37	1	0	6.652712	2.064732	-1.301258
38	1	0	7.721986	-1.711771	1.427160
39	6	0	-3.466603	-0.473089	0.405161

40	6	0	-3.718076	0.826477	-0.149626
41	6	0	-3.966295	-0.809509	1.659379
42	6	0	-3.252312	1.212770	-1.440098
43	6	0	-4.475103	1.782517	0.606059
44	6	0	-4.717965	0.154033	2.387518
45	6	0	-3.508993	2.470216	-1.946618
46	1	0	-2.690050	0.501826	-2.035521
47	6	0	-4.723671	3.071154	0.056209
48	6	0	-4.965079	1.410609	1.885678
49	1	0	-5.096885	-0.116474	3.369899
50	6	0	-4.250762	3.413285	-1.192336
51	1	0	-3.142314	2.739270	-2.933173
52	1	0	-5.299504	3.783709	0.641653
53	1	0	-5.537797	2.131929	2.463134
54	1	0	-4.447656	4.399794	-1.601929
55	6	0	-3.717656	-2.162190	2.291497
56	1	0	-2.690851	-2.502423	2.128985
57	1	0	-4.379340	-2.931628	1.876186
58	1	0	-3.898162	-2.121637	3.369605
59	6	0	2.024257	2.696252	-1.184634
60	1	0	2.269036	3.484326	-1.901568
61	1	0	1.536791	3.162735	-0.321387
62	1	0	1.283124	2.035480	-1.646781

2b-syn-GS1

Method: opt freq b3lyp/6-31+g(d,p) scrf(solvent=Chloroform)
SCF Done: E(RB3LYP) = -1461.81205939 A.U. after 1 cycles
Lowest frequency = 9.6479

Zero-point correction= 0.499738
(Hartree/Particle)
Thermal correction to Energy= 0.528737
Thermal correction to Enthalpy= 0.529681
Thermal correction to Gibbs Free Energy= 0.437627
Sum of electronic and zero-point Energies= -1461.312322
Sum of electronic and thermal Energies= -1461.283323
Sum of electronic and thermal Enthalpies= -1461.282378
Sum of electronic and thermal Free Energies= -1461.374432

Standard orientation:

Center Number	Atomic Number	Atomic Type	Coordinates (Angstroms) X	Y	Z
1	6	0	0.850067	2.846092	-0.709467
2	6	0	-0.533112	2.671478	-0.738839
3	6	0	-1.142507	1.432275	-0.419043
4	6	0	-0.280555	0.348376	-0.153298
5	6	0	1.126236	0.500242	-0.117496
6	6	0	1.666450	1.764171	-0.390951
7	8	0	-0.842707	-0.856989	0.154506
8	6	0	-0.062311	-2.026965	-0.163304
9	6	0	1.368041	-1.880590	0.264935
10	6	0	1.946919	-0.665962	0.264174
11	8	0	-1.297104	3.768612	-1.030626
12	6	0	-2.558268	3.484506	-1.668916
13	6	0	-3.296093	2.399906	-0.943400
14	6	0	-2.621852	1.393980	-0.351481
15	1	0	1.265850	3.819642	-0.945089

16	1	0	2.743184	1.896485	-0.361122
17	1	0	-0.561756	-2.848484	0.353927
18	1	0	-0.140387	-2.212070	-1.248149
19	1	0	1.919593	-2.774507	0.542245
20	1	0	-3.107058	4.427860	-1.657208
21	1	0	-2.363680	3.213634	-2.720408
22	1	0	-4.380513	2.447503	-0.901839
23	6	0	3.380216	-0.474266	0.658463
24	6	0	4.415300	-0.667184	-0.317124
25	6	0	3.703086	-0.111353	1.964309
26	6	0	4.138894	-1.018080	-1.671427
27	6	0	5.787488	-0.494104	0.067458
28	6	0	5.069772	0.053973	2.320485
29	6	0	5.156613	-1.194337	-2.585504
30	1	0	3.107884	-1.149010	-1.981559
31	6	0	6.814614	-0.687763	-0.898646
32	6	0	6.082405	-0.131732	1.407531
33	1	0	5.311014	0.331707	3.343158
34	6	0	6.510546	-1.031009	-2.197958
35	1	0	4.919774	-1.460101	-3.611816
36	1	0	7.849032	-0.557640	-0.590525
37	1	0	7.120167	-0.000458	1.703129
38	1	0	7.303035	-1.175411	-2.926416
39	6	0	-3.374480	0.351264	0.421249
40	6	0	-4.096679	-0.672629	-0.279006
41	6	0	-3.407917	0.408060	1.812054
42	6	0	-4.077062	-0.796222	-1.699185
43	6	0	-4.857473	-1.634131	0.467219
44	6	0	-4.166723	-0.557782	2.528227
45	6	0	-4.778887	-1.795235	-2.341817
46	1	0	-3.496073	-0.089643	-2.281245
47	6	0	-5.576492	-2.648650	-0.225115
48	6	0	-4.871826	-1.547628	1.884017
49	1	0	-4.190577	-0.499077	3.613436
50	6	0	-5.542878	-2.730878	-1.600302
51	1	0	-4.744217	-1.867387	-3.425275
52	1	0	-6.154186	-3.363527	0.355680
53	1	0	-5.449958	-2.271862	2.452328
54	1	0	-6.094982	-3.510656	-2.116902
55	6	0	-2.651960	1.463007	2.588233
56	1	0	-1.575432	1.253155	2.600494
57	1	0	-2.778568	2.457896	2.150391
58	1	0	-2.993039	1.500031	3.626507
59	6	0	2.637411	0.097260	3.017145
60	1	0	3.080366	0.440793	3.955651
61	1	0	1.897174	0.839556	2.699946
62	1	0	2.089961	-0.829945	3.220298

2b-syn-GS2

Method: opt freq b3lyp/6-31+g(d,p) scrf(solvent=Chloroform)

SCF Done: E(RB3LYP) = -1461.81252926 A.U. after 1 cycles

Lowest frequency = 9.6660

Zero-point correction= 0.499838
(Hartree/Particle)

Thermal correction to Energy= 0.528825

Thermal correction to Enthalpy= 0.529769

Thermal correction to Gibbs Free Energy= 0.437685

Sum of electronic and zero-point Energies= -1461.312691

Sum of electronic and thermal Energies= -1461.283705

Sum of electronic and thermal Enthalpies= -1461.282761
Sum of electronic and thermal Free Energies= -1461.374844

Standard orientation:

Center Number	Atomic Number	Atomic Type	Coordinates (Angstroms) X	Y	Z
1	6	0	0.591220	-1.938409	2.169463
2	6	0	-0.763744	-2.000749	1.844151
3	6	0	-1.294957	-1.337222	0.709033
4	6	0	-0.376750	-0.672413	-0.127660
5	6	0	1.008026	-0.630639	0.157269
6	6	0	1.464279	-1.257634	1.324928
7	8	0	-0.848227	-0.127471	-1.287501
8	6	0	-0.129937	1.018163	-1.789481
9	6	0	1.354206	0.795892	-1.770438
10	6	0	1.904380	0.018850	-0.819241
11	8	0	-1.586466	-2.664384	2.711903
12	6	0	-2.767353	-3.238948	2.115280
13	6	0	-3.473024	-2.236680	1.251892
14	6	0	-2.767765	-1.330557	0.546662
15	1	0	0.944466	-2.439126	3.064314
16	1	0	2.524081	-1.234962	1.557712
17	1	0	-0.414171	1.897970	-1.188486
18	1	0	-0.507432	1.166877	-2.803004
19	1	0	1.962178	1.295728	-2.519306
20	1	0	-3.386414	-3.562621	2.953686
21	1	0	-2.467542	-4.132856	1.543099
22	1	0	-4.558484	-2.253246	1.216186
23	6	0	3.383290	-0.213492	-0.746830
24	6	0	4.218810	0.738332	-0.071475
25	6	0	3.940650	-1.348573	-1.332002
26	6	0	3.697487	1.904277	0.563033
27	6	0	5.636103	0.516895	-0.014901
28	6	0	5.347276	-1.545027	-1.262742
29	6	0	4.527246	2.798523	1.206562
30	1	0	2.628622	2.085957	0.534741
31	6	0	6.465539	1.461366	0.652850
32	6	0	6.172550	-0.644977	-0.628357
33	1	0	5.772701	-2.430417	-1.727840
34	6	0	5.927324	2.579609	1.251472
35	1	0	4.105256	3.678272	1.684021
36	1	0	7.537310	1.281855	0.683079
37	1	0	7.245341	-0.815842	-0.588916
38	1	0	6.570455	3.292650	1.758930
39	6	0	-3.491112	-0.312658	-0.285251
40	6	0	-3.627892	1.029050	0.205863
41	6	0	-4.062230	-0.676612	-1.500858
42	6	0	-3.085780	1.447649	1.456248
43	6	0	-4.343082	1.997644	-0.574762
44	6	0	-4.771805	0.299869	-2.253389
45	6	0	-3.232045	2.745446	1.901375
46	1	0	-2.553749	0.729410	2.070340
47	6	0	-4.476816	3.328351	-0.088374
48	6	0	-4.908892	1.595913	-1.813357
49	1	0	-5.207414	0.006646	-3.205215
50	6	0	-3.932539	3.699864	1.122152
51	1	0	-2.809834	3.037828	2.858641
52	1	0	-5.022778	4.049637	-0.691507

53	1	0	-5.451501	2.325723	-2.408936
54	1	0	-4.042644	4.718215	1.483464
55	6	0	-3.932925	-2.074496	-2.066286
56	1	0	-4.610423	-2.777951	-1.568007
57	1	0	-2.918818	-2.466298	-1.945511
58	1	0	-4.176962	-2.081786	-3.132444
59	6	0	3.088378	-2.370841	-2.050577
60	1	0	2.580537	-1.930323	-2.915687
61	1	0	2.307568	-2.775245	-1.397240
62	1	0	3.698491	-3.205833	-2.404862

2b-TS1-A-int-B-fix-down
Method: opt(ts,calcfc,noeigen) b3lyp/6-31+g(d,p) freq scrf(solvent=Chlorofor
SCF Done: E(RB3LYP) = -1461.76395522 A.U. after 1 cycles
Lowest frequency = -28.3606

Zero-point correction= 0.500654
(Hartree/Particle)
Thermal correction to Energy= 0.527990
Thermal correction to Enthalpy= 0.528934
Thermal correction to Gibbs Free Energy= 0.443339
Sum of electronic and zero-point Energies= -1461.263301
Sum of electronic and thermal Energies= -1461.235965
Sum of electronic and thermal Enthalpies= -1461.235021
Sum of electronic and thermal Free Energies= -1461.320616

Standard orientation:

Center Number	Atomic Number	Atomic Type	Coordinates (Angstroms) X	Y	Z
1	6	0	-0.015140	3.607293	-0.132594
2	6	0	-1.252285	3.064609	0.206850
3	6	0	-1.490973	1.671842	0.110513
4	6	0	-0.381003	0.859616	-0.193479
5	6	0	0.938823	1.364670	-0.310081
6	6	0	1.057967	2.758548	-0.397976
7	8	0	-0.588875	-0.471783	-0.437293
8	6	0	0.187259	-0.895886	-1.579464
9	6	0	2.038034	0.360208	-0.522053
10	8	0	-2.267723	3.931324	0.502211
11	6	0	-3.303154	3.380428	1.341919
12	6	0	-3.769586	2.056794	0.813351
13	6	0	-2.895230	1.214203	0.228443
14	1	0	0.101808	4.685248	-0.166891
15	1	0	2.001739	3.199983	-0.680168
16	1	0	-0.030982	-1.958376	-1.705569
17	1	0	-0.175292	-0.360646	-2.472545
18	1	0	-2.908998	3.295463	2.368465
19	1	0	-4.101911	4.123943	1.343410
20	1	0	-4.820780	1.799018	0.904963
21	6	0	3.452133	0.385351	-0.003996
22	6	0	4.191076	-0.886450	-0.002626
23	6	0	4.178164	1.507190	0.444012
24	6	0	3.571271	-2.161452	0.173411
25	6	0	5.625810	-0.888792	-0.042405
26	6	0	5.603859	1.465109	0.423423
27	6	0	4.293513	-3.339191	0.170363
28	1	0	2.506812	-2.211326	0.362662

29	6	0	6.343053	-2.115696	-0.113908
30	6	0	6.315649	0.343552	0.091145
31	1	0	6.133660	2.380292	0.676586
32	6	0	5.693081	-3.326718	-0.028344
33	1	0	3.777886	-4.280512	0.337481
34	1	0	7.426586	-2.073423	-0.190071
35	1	0	7.400315	0.363961	0.031760
36	1	0	6.250849	-4.257889	-0.063259
37	6	0	-3.388530	-0.081376	-0.346303
38	6	0	-3.608926	-1.205787	0.516275
39	6	0	-3.681452	-0.168282	-1.704635
40	6	0	-3.313231	-1.174465	1.910317
41	6	0	-4.131030	-2.424133	-0.033742
42	6	0	-4.196054	-1.386964	-2.226140
43	6	0	-3.532051	-2.274590	2.713187
44	1	0	-2.906782	-0.266553	2.342240
45	6	0	-4.351148	-3.540065	0.821849
46	6	0	-4.415807	-2.482535	-1.423153
47	1	0	-4.425215	-1.442805	-3.287322
48	6	0	-4.060672	-3.471282	2.167135
49	1	0	-3.295255	-2.225462	3.772271
50	1	0	-4.753712	-4.453539	0.391061
51	1	0	-4.813752	-3.402700	-1.843513
52	1	0	-4.231941	-4.330169	2.809576
53	6	0	-3.467124	0.998796	-2.643218
54	1	0	-2.404058	1.247983	-2.742379
55	1	0	-3.849897	0.770054	-3.641441
56	1	0	-3.970122	1.902561	-2.283828
57	6	0	3.631233	2.778930	1.067180
58	1	0	3.667946	3.644363	0.395596
59	1	0	2.617404	2.672817	1.444452
60	1	0	4.272288	3.024740	1.920664
61	6	0	1.641093	-0.677631	-1.305356
62	1	0	2.332452	-1.418408	-1.682931

2b-TS4-A-fix-up-B-int

Method: opt(ts,calcfc,noeigen) b3lyp/6-31+g(d,p) freq scrf(solvent=Chlorofor
SCF Done: E(RB3LYP) = -1461.76076505 A.U. after 1 cycles
Lowest frequency = -29.3997

Zero-point correction= 0.500226
(Hartree/Particle)
Thermal correction to Energy= 0.527692
Thermal correction to Enthalpy= 0.528637
Thermal correction to Gibbs Free Energy= 0.442409
Sum of electronic and zero-point Energies= -1461.260539
Sum of electronic and thermal Energies= -1461.233073
Sum of electronic and thermal Enthalpies= -1461.232128
Sum of electronic and thermal Free Energies= -1461.318356

Standard orientation:

Center Number	Atomic Number	Atomic Type	Coordinates (Angstroms) X	Y	Z
1	6	0	-0.566958	-2.172266	-1.710893
2	6	0	0.708648	-2.009545	-1.172052
3	6	0	0.966547	-1.164596	-0.062047

4	6	0	-0.161244	-0.869216	0.727200
5	6	0	-1.477565	-1.008025	0.216196
6	6	0	-1.649302	-1.609486	-1.038180
7	8	0	0.041494	-0.436566	2.002140
8	6	0	-1.044998	-0.628148	2.934595
9	6	0	-2.372700	-0.265739	2.338767
10	6	0	-2.594265	-0.490122	1.030549
11	8	0	1.743938	-2.766642	-1.660655
12	6	0	2.555795	-3.251570	-0.551301
13	6	0	3.151452	-2.069144	0.151499
14	6	0	2.419639	-0.930244	0.250244
15	1	0	-0.700524	-2.781923	-2.597855
16	1	0	-2.650276	-1.705788	-1.446478
17	1	0	-1.026994	-1.680558	3.263481
18	1	0	-0.794761	-0.002350	3.793931
19	1	0	-3.141937	0.141205	2.988601
20	1	0	1.921567	-3.886792	0.084988
21	1	0	3.331513	-3.871431	-1.004308
22	1	0	4.209107	-2.092155	0.383212
23	6	0	-3.923204	-0.221737	0.392532
24	6	0	-4.118118	0.983592	-0.364482
25	6	0	-4.961243	-1.139594	0.530586
26	6	0	-3.090232	1.955746	-0.533390
27	6	0	-5.392417	1.237656	-0.971709
28	6	0	-6.216726	-0.864681	-0.082284
29	6	0	-3.307585	3.105700	-1.263884
30	1	0	-2.122068	1.785503	-0.075599
31	6	0	-5.586357	2.433725	-1.717502
32	6	0	-6.431034	0.282677	-0.808752
33	1	0	-7.019143	-1.589370	0.028744
34	6	0	-4.567761	3.350535	-1.864242
35	1	0	-2.506858	3.830490	-1.379654
36	1	0	-6.558230	2.612373	-2.170698
37	1	0	-7.397445	0.469229	-1.270107
38	1	0	-4.727249	4.259958	-2.436099
39	6	0	3.162653	0.375432	0.407797
40	6	0	4.515110	0.421746	-0.167247
41	6	0	2.722547	1.546273	1.050297
42	6	0	4.942542	-0.394374	-1.261075
43	6	0	5.455874	1.411958	0.276076
44	6	0	3.678989	2.525603	1.447807
45	6	0	6.219589	-0.323396	-1.780139
46	1	0	4.235400	-1.065935	-1.729709
47	6	0	6.787390	1.421972	-0.228485
48	6	0	5.014087	2.428875	1.158256
49	1	0	3.316144	3.375626	2.020122
50	6	0	7.176358	0.561088	-1.229040
51	1	0	6.484295	-0.947087	-2.629229
52	1	0	7.481083	2.158479	0.169038
53	1	0	5.720752	3.169895	1.522093
54	1	0	8.188876	0.588065	-1.620856
55	6	0	1.292373	1.945916	1.339134
56	1	0	0.586903	1.508667	0.636091
57	1	0	0.972768	1.680852	2.348844
58	1	0	1.219717	3.034036	1.242742
59	6	0	-4.806564	-2.424297	1.316217
60	1	0	-3.817469	-2.870370	1.182320
61	1	0	-4.938988	-2.251930	2.391286
62	1	0	-5.557571	-3.156916	1.006937

2b-TS2-A-ext-B-fix-up
Method: opt(ts,calcfc,noeigen) b3lyp/6-31+g(d,p) freq scrf(solvent=Chlorofor
SCF Done: E(RB3LYP) = -1461.75091465 A.U. after 2 cycles
Lowest frequency = -68.6439

Zero-point correction= 0.500548
(Hartree/Particle)
Thermal correction to Energy= 0.528023
Thermal correction to Enthalpy= 0.528967
Thermal correction to Gibbs Free Energy= 0.442470
Sum of electronic and zero-point Energies= -1461.250366
Sum of electronic and thermal Energies= -1461.222892
Sum of electronic and thermal Enthalpies= -1461.221947
Sum of electronic and thermal Free Energies= -1461.308445

Standard orientation:

Center Number	Atomic Number	Atomic Type	Coordinates (Angstroms) X	Y	Z
1	6	0	0.597488	2.575994	-1.520720
2	6	0	-0.439291	2.591231	-0.591539
3	6	0	-0.910207	1.388998	-0.011472
4	6	0	-0.175738	0.219807	-0.295574
5	6	0	1.051172	0.221590	-1.016098
6	6	0	1.317786	1.401639	-1.728240
7	8	0	-0.710753	-0.983313	0.069360
8	6	0	-0.494244	-1.940707	-0.997263
9	6	0	1.821640	-1.086170	-1.033698
10	8	0	-1.086319	3.775916	-0.381049
11	6	0	-1.763715	3.876913	0.888669
12	6	0	-2.609175	2.664266	1.138972
13	6	0	-2.194300	1.450165	0.726289
14	1	0	0.844908	3.484773	-2.058726
15	1	0	2.078629	1.417684	-2.492701
16	1	0	-0.992779	-2.856817	-0.677665
17	1	0	-0.991598	-1.563617	-1.903624
18	1	0	-1.004281	4.015457	1.676144
19	1	0	-2.362094	4.787342	0.826641
20	1	0	-3.557831	2.787016	1.653433
21	6	0	3.257270	-1.253679	-0.545966
22	6	0	4.128944	-0.118530	-0.239059
23	6	0	3.751399	-2.513789	-0.128689
24	6	0	4.188934	1.071426	-1.012353
25	6	0	5.098339	-0.210358	0.819984
26	6	0	4.784113	-2.584719	0.849017
27	6	0	4.987270	2.146811	-0.672363
28	1	0	3.666456	1.105353	-1.950220
29	6	0	5.864649	0.927433	1.197996
30	6	0	5.363950	-1.473288	1.401195
31	1	0	5.079448	-3.570982	1.196635
32	6	0	5.796010	2.103473	0.484646
33	1	0	5.009080	3.020197	-1.317925
34	1	0	6.548879	0.828046	2.036765
35	1	0	6.085476	-1.557048	2.209041
36	1	0	6.398557	2.961102	0.768512
37	6	0	-3.064042	0.252348	0.971990
38	6	0	-3.904424	-0.235533	-0.083485
39	6	0	-3.090539	-0.348429	2.226140

40	6	0	-3.927509	0.356095	-1.379996
41	6	0	-4.762261	-1.359455	0.159362
42	6	0	-3.952337	-1.459151	2.444075
43	6	0	-4.743707	-0.137465	-2.377198
44	1	0	-3.295062	1.213953	-1.581520
45	6	0	-5.593618	-1.844215	-0.888915
46	6	0	-4.761752	-1.954937	1.448512
47	1	0	-3.959909	-1.926366	3.425596
48	6	0	-5.586643	-1.250400	-2.132884
49	1	0	-4.743000	0.332195	-3.356788
50	1	0	-6.240225	-2.695004	-0.688295
51	1	0	-5.409093	-2.807530	1.637760
52	1	0	-6.226816	-1.628839	-2.924545
53	6	0	-2.216383	0.129591	3.365420
54	1	0	-1.197493	0.346492	3.031341
55	1	0	-2.608331	1.048889	3.816690
56	1	0	-2.162093	-0.627423	4.152889
57	6	0	3.290487	-3.878377	-0.603702
58	1	0	2.434367	-4.263275	-0.034641
59	1	0	3.032860	-3.892457	-1.665353
60	1	0	4.107285	-4.591023	-0.458595
61	6	0	0.979931	-2.151117	-1.143848
62	1	0	1.304252	-3.173701	-1.142567

2b-TS4-A-fix-down-B-int

Method: opt(ts,calcfc,noeigen) b3lyp/6-31+g(d,p) freq scrf(solvent=Chlorofor
SCF Done: E(RB3LYP) = -1461.76091893 A.U. after 1 cycles
Lowest frequency = -24.5050

Zero-point correction= 0.500289
(Hartree/Particle)
Thermal correction to Energy= 0.527736
Thermal correction to Enthalpy= 0.528680
Thermal correction to Gibbs Free Energy= 0.442413
Sum of electronic and zero-point Energies= -1461.260630
Sum of electronic and thermal Energies= -1461.233183
Sum of electronic and thermal Enthalpies= -1461.232239
Sum of electronic and thermal Free Energies= -1461.318506

Standard orientation:

Center Number	Atomic Number	Atomic Type	Coordinates (Angstroms) X	Y	Z
1	6	0	0.556135	2.229086	-1.225031
2	6	0	-0.704133	2.047317	-0.656686
3	6	0	-1.030908	0.913028	0.130199
4	6	0	0.077187	0.233297	0.670552
5	6	0	1.374447	0.375742	0.113977
6	6	0	1.575275	1.344360	-0.879221
7	8	0	-0.125633	-0.577092	1.745517
8	6	0	1.021141	-0.839653	2.584040
9	6	0	2.250011	-1.144851	1.780177
10	6	0	2.437286	-0.524872	0.600257
11	8	0	-1.640602	3.044124	-0.770988
12	6	0	-2.322854	3.217268	0.505566
13	6	0	-3.050421	1.947373	0.829405
14	6	0	-2.486642	0.763272	0.479390

15	1	0	0.736722	3.085119	-1.865986
16	1	0	2.560503	1.454401	-1.320865
17	1	0	1.173440	0.038506	3.233397
18	1	0	0.723911	-1.678889	3.216377
19	1	0	2.979832	-1.839885	2.185117
20	1	0	-1.575314	3.516689	1.255301
21	1	0	-3.020484	4.043141	0.355869
22	1	0	-4.081741	2.017534	1.152286
23	6	0	3.663622	-0.747836	-0.231361
24	6	0	4.872197	-0.040245	0.084885
25	6	0	3.628385	-1.635056	-1.305570
26	6	0	4.956570	0.898977	1.154825
27	6	0	6.048337	-0.265929	-0.707039
28	6	0	4.806797	-1.841107	-2.073897
29	6	0	6.132861	1.564374	1.430435
30	1	0	4.076445	1.088700	1.759335
31	6	0	7.248082	0.432595	-0.393065
32	6	0	5.980865	-1.182946	-1.787919
33	1	0	4.770550	-2.541373	-2.904242
34	6	0	7.294442	1.328589	0.652605
35	1	0	6.170213	2.276634	2.249785
36	1	0	8.131344	0.246325	-0.998828
37	1	0	6.869777	-1.358157	-2.388522
38	1	0	8.215476	1.856628	0.881534
39	6	0	-3.400778	-0.420090	0.266388
40	6	0	-4.772527	-0.107580	-0.161169
41	6	0	-3.102137	-1.782998	0.441810
42	6	0	-5.131818	1.075752	-0.879366
43	6	0	-5.822846	-1.070277	0.018055
44	6	0	-4.168734	-2.718261	0.582327
45	6	0	-6.429964	1.337307	-1.269036
46	1	0	-4.359747	1.777468	-1.166019
47	6	0	-7.165364	-0.750236	-0.332402
48	6	0	-5.488666	-2.371286	0.468863
49	1	0	-3.904038	-3.748828	0.805035
50	6	0	-7.475590	0.438853	-0.951584
51	1	0	-6.643875	2.238791	-1.836121
52	1	0	-7.937224	-1.489963	-0.135440
53	1	0	-6.275559	-3.102517	0.632806
54	1	0	-8.499642	0.667020	-1.232105
55	6	0	-1.732021	-2.424608	0.447420
56	1	0	-1.003018	-1.862277	-0.131630
57	1	0	-1.332234	-2.561436	1.454201
58	1	0	-1.821490	-3.414609	-0.011498
59	6	0	2.371274	-2.392464	-1.669991
60	1	0	1.992653	-2.972400	-0.821661
61	1	0	1.567581	-1.712370	-1.974140
62	1	0	2.558735	-3.081866	-2.497251

2b-TS3-A-fix-down-B-ext

Method: opt(ts,calcfc,noeigen) b3lyp/6-31+g(d,p) freq scrf(solvent=Chlorofor
SCF Done: E(RB3LYP) = -1461.76050922 A.U. after 1 cycles
Lowest frequency = -31.3555

Zero-point correction= 0.500215
(Hartree/Particle)
Thermal correction to Energy= 0.527713
Thermal correction to Enthalpy= 0.528657
Thermal correction to Gibbs Free Energy= 0.442196

Sum of electronic and zero-point Energies= -1461.260294
Sum of electronic and thermal Energies= -1461.232796
Sum of electronic and thermal Enthalpies= -1461.231852
Sum of electronic and thermal Free Energies= -1461.318313

Standard orientation:

Center Number	Atomic Number	Atomic Type	Coordinates (Angstroms) X	Y	Z
1	6	0	0.585932	2.618711	-1.105778
2	6	0	-0.713707	2.521394	-0.610708
3	6	0	-1.194499	1.364744	0.058999
4	6	0	-0.185790	0.529556	0.578630
5	6	0	1.146001	0.583712	0.091918
6	6	0	1.493828	1.608120	-0.798591
7	8	0	-0.518857	-0.357177	1.560385
8	6	0	0.553009	-0.798847	2.422872
9	6	0	1.784495	-1.158450	1.646342
10	6	0	2.088543	-0.461671	0.535542
11	8	0	-1.535567	3.617629	-0.683710
12	6	0	-2.273854	3.759704	0.564504
13	6	0	-3.139655	2.549382	0.739278
14	6	0	-2.678239	1.344272	0.321609
15	1	0	0.882118	3.502662	-1.659991
16	1	0	2.506335	1.653195	-1.186546
17	1	0	0.755612	0.005637	3.149312
18	1	0	0.143402	-1.651794	2.967160
19	1	0	2.421131	-1.957622	2.014719
20	1	0	-1.547960	3.920135	1.375510
21	1	0	-2.876261	4.662329	0.448547
22	1	0	-4.178994	2.694507	1.008633
23	6	0	3.323683	-0.737784	-0.266503
24	6	0	4.583142	-0.198535	0.163103
25	6	0	3.247108	-1.509944	-1.424281
26	6	0	4.714500	0.620118	1.323526
27	6	0	5.765389	-0.473504	-0.603813
28	6	0	4.432689	-1.768233	-2.165311
29	6	0	5.939298	1.125573	1.706623
30	1	0	3.831577	0.845354	1.911470
31	6	0	7.015111	0.058611	-0.178476
32	6	0	5.653960	-1.270792	-1.772412
33	1	0	4.363094	-2.378444	-3.061951
34	6	0	7.105271	0.840972	0.952056
35	1	0	6.011964	1.748479	2.593612
36	1	0	7.901567	-0.163457	-0.767277
37	1	0	6.547161	-1.483051	-2.354397
38	1	0	8.064366	1.242613	1.265689
39	6	0	-3.734396	0.331421	-0.067494
40	6	0	-3.677308	-1.112836	0.119814
41	6	0	-4.931905	0.849273	-0.610741
42	6	0	-2.471912	-1.860763	0.184589
43	6	0	-4.895868	-1.878398	0.141492
44	6	0	-6.112086	0.051669	-0.621787
45	6	0	-2.460888	-3.228901	0.378973
46	1	0	-1.532902	-1.362740	0.034528
47	6	0	-4.862249	-3.277111	0.395209
48	6	0	-6.123236	-1.243591	-0.178539
49	1	0	-7.029835	0.504523	-0.988469
50	6	0	-3.666840	-3.948612	0.528761

51	1	0	-1.510499	-3.754883	0.400802
52	1	0	-5.806483	-3.813859	0.440178
53	1	0	-7.045638	-1.817457	-0.148557
54	1	0	-3.649994	-5.020184	0.704773
55	6	0	-5.097260	2.196675	-1.300112
56	1	0	-5.668471	2.916019	-0.701633
57	1	0	-4.146143	2.660028	-1.561364
58	1	0	-5.664978	2.039141	-2.224434
59	6	0	1.936494	-2.087114	-1.910002
60	1	0	1.442657	-2.674309	-1.128789
61	1	0	1.235969	-1.297536	-2.205145
62	1	0	2.094031	-2.735844	-2.775523

2b-TS3-A-fix-up-B-ext

Method: opt(ts,calcfc,noeigen) b3lyp/6-31+g(d,p) freq scrf(solvent=Chlorofor
SCF Done: E(RB3LYP) = -1461.76041610 A.U. after 1 cycles
Lowest frequency = -34.2921

Zero-point correction= 0.500253
(Hartree/Particle)
Thermal correction to Energy= 0.527702
Thermal correction to Enthalpy= 0.528646
Thermal correction to Gibbs Free Energy= 0.442799
Sum of electronic and zero-point Energies= -1461.260163
Sum of electronic and thermal Energies= -1461.232715
Sum of electronic and thermal Enthalpies= -1461.231770
Sum of electronic and thermal Free Energies= -1461.317618

Standard orientation:

Center Number	Atomic Number	Atomic Type	Coordinates (Angstroms) X	Y	Z
1	6	0	-0.559892	-2.784175	-1.244822
2	6	0	0.768137	-2.583099	-0.871550
3	6	0	1.175403	-1.507817	-0.037742
4	6	0	0.141042	-0.943770	0.734855
5	6	0	-1.223415	-1.110313	0.382434
6	6	0	-1.544631	-1.994933	-0.655551
7	8	0	0.479600	-0.209742	1.833759
8	6	0	-0.522236	-0.091334	2.868127
9	6	0	-1.878599	0.208849	2.303708
10	6	0	-2.230645	-0.319958	1.117296
11	8	0	1.709690	-3.510295	-1.241179
12	6	0	2.612326	-3.762377	-0.125410
13	6	0	3.328414	-2.486590	0.198016
14	6	0	2.665034	-1.309999	0.074562
15	1	0	-0.806396	-3.585304	-1.933037
16	1	0	-2.583458	-2.118117	-0.944519
17	1	0	-0.525752	-1.031513	3.444346
18	1	0	-0.160262	0.705087	3.520991
19	1	0	-2.561119	0.824516	2.882202
20	1	0	2.022142	-4.175514	0.706124
21	1	0	3.307606	-4.526823	-0.476539
22	1	0	4.403501	-2.518588	0.328435
23	6	0	-3.594129	-0.117317	0.530289
24	6	0	-3.781949	0.856048	-0.509796
25	6	0	-4.671243	-0.871448	0.988716
26	6	0	-2.714453	1.655021	-1.012112

27	6	0	-5.088846	1.049645	-1.067842
28	6	0	-5.958863	-0.660385	0.418492
29	6	0	-2.925553	2.583669	-2.010295
30	1	0	-1.719901	1.528860	-0.598879
31	6	0	-5.275239	2.016390	-2.094944
32	6	0	-6.166675	0.266882	-0.574954
33	1	0	-6.791972	-1.256812	0.781325
34	6	0	-4.218390	2.768743	-2.560450
35	1	0	-2.094278	3.178674	-2.377431
36	1	0	-6.272096	2.151888	-2.506924
37	1	0	-7.158311	0.407962	-0.997322
38	1	0	-4.372472	3.503602	-3.345164
39	6	0	3.522872	-0.095119	-0.210046
40	6	0	3.302781	1.265834	0.262675
41	6	0	4.699123	-0.318478	-0.960977
42	6	0	2.028307	1.797993	0.592772
43	6	0	4.404098	2.191021	0.312884
44	6	0	5.754739	0.637487	-0.931801
45	6	0	1.863438	3.090881	1.051999
46	1	0	1.151368	1.197653	0.442208
47	6	0	4.220823	3.499529	0.838478
48	6	0	5.653272	1.818025	-0.245920
49	1	0	6.669772	0.402807	-1.469782
50	6	0	2.974642	3.946500	1.219296
51	1	0	0.863055	3.453653	1.271140
52	1	0	5.085058	4.156603	0.893904
53	1	0	6.489585	2.510191	-0.195562
54	1	0	2.840703	4.953669	1.602965
55	6	0	4.949047	-1.477838	-1.915695
56	1	0	5.678918	-2.199500	-1.530535
57	1	0	4.040244	-2.023854	-2.167689
58	1	0	5.370703	-1.071541	-2.842309
59	6	0	-4.526846	-1.911504	2.079136
60	1	0	-3.581291	-2.455090	2.005151
61	1	0	-4.556903	-1.451842	3.074550
62	1	0	-5.344117	-2.636752	2.028742

2b-TS2-A-ext-B-fix-down

Method: opt(ts,calcfc,noeigen) b3lyp/6-31+g(d,p) freq scrf(solvent=Chlorofor
SCF Done: E(RB3LYP) = -1461.75043526 A.U. after 1 cycles
Lowest frequency = -67.9539

Zero-point correction= 0.500441
(Hartree/Particle)
Thermal correction to Energy= 0.527952
Thermal correction to Enthalpy= 0.528896
Thermal correction to Gibbs Free Energy= 0.442260
Sum of electronic and zero-point Energies= -1461.249995
Sum of electronic and thermal Energies= -1461.222483
Sum of electronic and thermal Enthalpies= -1461.221539
Sum of electronic and thermal Free Energies= -1461.308175

Standard orientation:

Center Number	Atomic Number	Atomic Type	Coordinates (Angstroms) X	Y	Z
1	6	0	0.847962	3.086076	-0.795700
2	6	0	-0.350289	2.855109	-0.125457

3	6	0	-0.940849	1.568908	-0.120364
4	6	0	-0.178071	0.519786	-0.672144
5	6	0	1.167555	0.676538	-1.108162
6	6	0	1.581693	2.006676	-1.283069
7	8	0	-0.787930	-0.687127	-0.866216
8	6	0	-0.365516	-1.236552	-2.138370
9	6	0	1.916830	-0.599130	-1.446393
10	8	0	-1.009903	3.934177	0.390831
11	6	0	-1.912239	3.625214	1.473582
12	6	0	-2.809997	2.481642	1.106877
13	6	0	-2.343564	1.470921	0.346664
14	1	0	1.207599	4.102889	-0.911071
15	1	0	2.479022	2.230211	-1.837952
16	1	0	-0.925387	-2.164706	-2.260058
17	1	0	-0.663523	-0.535009	-2.932752
18	1	0	-1.311961	3.403436	2.371631
19	1	0	-2.473014	4.543538	1.655206
20	1	0	-3.837574	2.490429	1.458673
21	6	0	3.227905	-1.031605	-0.797364
22	6	0	4.029607	-0.141077	0.043226
23	6	0	3.622713	-2.391273	-0.768582
24	6	0	4.257461	1.231559	-0.241929
25	6	0	4.764151	-0.664525	1.163860
26	6	0	4.436791	-2.872788	0.295831
27	6	0	4.979948	2.060530	0.594943
28	1	0	3.938474	1.622234	-1.190595
29	6	0	5.447437	0.212533	2.051766
30	6	0	4.898119	-2.066890	1.302919
31	1	0	4.650873	-3.937816	0.321585
32	6	0	5.535402	1.562681	1.794215
33	1	0	5.141723	3.096343	0.310422
34	1	0	5.946796	-0.219791	2.915076
35	1	0	5.440372	-2.478174	2.149670
36	1	0	6.075303	2.222250	2.467003
37	6	0	-3.271927	0.361796	-0.053472
38	6	0	-3.549988	-0.700733	0.868466
39	6	0	-3.902036	0.399865	-1.294276
40	6	0	-2.921114	-0.795361	2.144260
41	6	0	-4.483575	-1.726802	0.500469
42	6	0	-4.820897	-0.629488	-1.636954
43	6	0	-3.207201	-1.832184	3.007823
44	1	0	-2.202846	-0.036732	2.435112
45	6	0	-4.762070	-2.780272	1.416193
46	6	0	-5.107224	-1.661557	-0.772891
47	1	0	-5.310443	-0.588094	-2.606713
48	6	0	-4.140821	-2.835400	2.644942
49	1	0	-2.711202	-1.882893	3.972986
50	1	0	-5.476960	-3.546049	1.125277
51	1	0	-5.816877	-2.435319	-1.054836
52	1	0	-4.360734	-3.645252	3.334564
53	6	0	-3.633587	1.506361	-2.290227
54	1	0	-3.798767	2.494793	-1.848760
55	1	0	-2.595793	1.488195	-2.643304
56	1	0	-4.284639	1.410328	-3.163284
57	6	0	3.263020	-3.468126	-1.774205
58	1	0	2.315350	-3.971455	-1.542866
59	1	0	3.212549	-3.091284	-2.798419
60	1	0	4.038905	-4.238622	-1.749587
61	6	0	1.106050	-1.497039	-2.071680

62	1	0	1.415335	-2.482201	-2.362717

2b-TS1-A-int-B-fix-up-Chloroform-yoda-NM-M

Method: opt(ts,calcfc,noeigen) b3lyp/6-31+g(d,p) freq scrf(solvent=Chlorofor
SCF Done: E(RB3LYP) = -1461.76441419 A.U. after 1 cycles
Lowest frequency = -28.9342

Zero-point correction= 0.500750
(Hartree/Particle)
Thermal correction to Energy= 0.528046
Thermal correction to Enthalpy= 0.528991
Thermal correction to Gibbs Free Energy= 0.443462
Sum of electronic and zero-point Energies= -1461.263664
Sum of electronic and thermal Energies= -1461.236368
Sum of electronic and thermal Enthalpies= -1461.235424
Sum of electronic and thermal Free Energies= -1461.320952

Standard orientation:

Center Number	Atomic Number	Atomic Type	Coordinates (Angstroms) X	Y	Z
1	6	0	-0.202264	3.331918	-0.805523
2	6	0	-1.339134	2.910890	-0.119699
3	6	0	-1.491245	1.562239	0.286176
4	6	0	-0.382036	0.715424	0.092860
5	6	0	0.868661	1.175226	-0.391742
6	6	0	0.878149	2.462865	-0.945109
7	8	0	-0.529405	-0.625481	0.329874
8	6	0	0.089253	-1.385734	-0.732744
9	6	0	1.989188	0.173071	-0.447603
10	8	0	-2.362104	3.807196	0.021501
11	6	0	-3.241412	3.550896	1.135406
12	6	0	-3.677967	2.116521	1.151624
13	6	0	-2.830278	1.143241	0.764011
14	1	0	-0.166967	4.338335	-1.208880
15	1	0	1.738522	2.802037	-1.501745
16	1	0	-0.072559	-2.434926	-0.476742
17	1	0	-0.443378	-1.173674	-1.673855
18	1	0	-2.715223	3.828128	2.064259
19	1	0	-4.083209	4.232667	1.003480
20	1	0	-4.687463	1.882883	1.477313
21	6	0	3.459466	0.384875	-0.198921
22	6	0	4.270318	-0.806793	0.094288
23	6	0	4.169619	1.600786	-0.258153
24	6	0	3.764907	-1.962895	0.764376
25	6	0	5.681655	-0.803166	-0.167415
26	6	0	5.577115	1.572799	-0.487508
27	6	0	4.552520	-3.067468	1.025478
28	1	0	2.743913	-1.962866	1.123462
29	6	0	6.457754	-1.976543	0.046694
30	6	0	6.303639	0.413696	-0.547548
31	1	0	6.078367	2.527092	-0.630230
32	6	0	5.905343	-3.101853	0.616545
33	1	0	4.127213	-3.909762	1.563605
34	1	0	7.514047	-1.947277	-0.208348
35	1	0	7.364904	0.427703	-0.779801
36	1	0	6.510344	-3.985980	0.794584
37	6	0	-3.294073	-0.283760	0.769149

38	6	0	-3.722301	-0.888943	-0.459264
39	6	0	-3.353797	-0.996881	1.961927
40	6	0	-3.700649	-0.194686	-1.703988
41	6	0	-4.197627	-2.242522	-0.451456
42	6	0	-3.832558	-2.336395	1.945095
43	6	0	-4.115830	-0.803969	-2.870419
44	1	0	-3.357058	0.833786	-1.730687
45	6	0	-4.620043	-2.842651	-1.670635
46	6	0	-4.240047	-2.945976	0.781287
47	1	0	-3.867584	-2.887601	2.881406
48	6	0	-4.579789	-2.143127	-2.857697
49	1	0	-4.089997	-0.252351	-3.805926
50	1	0	-4.979948	-3.868355	-1.647585
51	1	0	-4.598340	-3.972258	0.792772
52	1	0	-4.906204	-2.611833	-3.781542
53	6	0	-2.904975	-0.401370	3.278954
54	1	0	-3.649891	0.294663	3.682681
55	1	0	-2.750392	-1.187161	4.023767
56	1	0	-1.969863	0.156572	3.173789
57	6	0	3.637300	2.999803	-0.005421
58	1	0	3.513488	3.590955	-0.920029
59	1	0	2.700862	3.011088	0.546412
60	1	0	4.380419	3.526476	0.602937
61	6	0	1.549905	-1.068159	-0.786094
62	1	0	2.225473	-1.880221	-1.017698
